# Optimizing MS-Based Multi-Omics: Comparative Analysis of Protein, Metabolite, and Lipid Extraction Techniques

**DOI:** 10.3390/metabo14010034

**Published:** 2024-01-03

**Authors:** Jeong-Hun Mok, Minjoong Joo, Seonghyeon Cho, Van-An Duong, Haneul Song, Jong-Moon Park, Hookeun Lee

**Affiliations:** 1 Department of Medical Device Management and Research, SAIHST, Sungkyunkwan University, 115, Irwon-ro, Gangnam-gu, Seoul 06355, Republic of Korea; jeonghunmok@naver.com; 2 Basilbiotech, 157-20, Sinsong-ro, Yeonsu-gu, Incheon 22002, Republic of Korea; mjjoo@basilbiotech.com (M.J.); shcho@basilbiotech.com (S.C.); 3 College of Pharmacy, Gachon University, 191, Hambangmoe-ro, Yeonsu-gu, Incheon 21936, Republic of Korea; anduong@gachon.ac.kr (V.-A.D.); 202240509@gachon.ac.kr (H.S.)

**Keywords:** sample preparation, proteomics, metabolomics, LC-MS

## Abstract

Multi-omics integrates diverse types of biological information from genomic, proteomic, and metabolomics experiments to achieve a comprehensive understanding of complex cellular mechanisms. However, this approach is also challenging due to technical issues such as limited sample quantities, the complexity of data pre-processing, and reproducibility concerns. Furthermore, existing studies have primarily focused on technical performance assessment and the presentation of modified protocols through quantitative comparisons of the identified protein counts. Nevertheless, the specific differences in these comparisons have been minimally investigated. Here, findings obtained from various omics approaches were profiled using various extraction methods (methanol extraction, the Folch method, and Matyash methods for metabolites and lipids) and two digestion methods (filter-aided sample preparation (FASP) and suspension traps (S-Trap)) for resuspended proteins. FASP was found to be more effective for the identification of membrane-related proteins, whereas S-Trap excelled in isolating nuclear-related and RNA-processing proteins. Thus, FASP may be suitable for investigating the immune response and bacterial infection pathways, whereas S-Trap may be more effective for studies focused on the mechanisms of neurodegenerative diseases. Moreover, regarding the choice of extraction method, the single-phase method identified organic compounds and compounds related to fatty acids, whereas the two-phase extraction method identified more hydrophilic compounds such as nucleotides. Lipids with strong hydrophobicity, such as ChE and TG, were identified in the two-phase extraction results. These findings highlight that significant differences among small molecules are primarily identified due to the varying polarities of extraction solvents. These results, obtained by considering variables such as human error and batch effects in the sample preparation step, offer comprehensive and detailed results not previously provided by existing studies, thereby aiding in the selection of the most suitable pre-processing approach.

## 1. Introduction

Omics research is based on the analysis of a comprehensive spectrum of biomolecules in biological samples [1,2,3,4]. Rapid advancements in analytics and information processing have made high-throughput omics technology a powerful tool in systems biology research [5,6,7,8]. Compared to single-omics, multi-omics can provide a more intricate picture of biological mechanisms by integrating information from independent layers of biomolecules [8,9,10]. This integration enhances data accuracy and comparability due to the close correlation among different molecules from the same sample. Mass spectrometry (MS)-based studies uniquely enable the concurrent and comprehensive profiling of both the proteome and metabolome, offering a holistic view of cellular processes [11,12]. However, the limited sample size in multi-omics analysis poses a challenge for extracting the desired proteome, metabolome, and lipidome [9,13,14]. To this end, the pre-processing stage is crucial for obtaining quality results. In particular, the simultaneous extraction of various biomolecules should be achieved to increase the concentration of analytes and minimize sample variability and, thereby, to ensure data consistency and reliability [9,15]. Recent studies explored variations of the Folch method using chloroform–methanol (MeOH) and the Matyash method using methyl tertiary–butyl ether (MTBE) as solvents for the isolation of proteins, metabolites, and lipids and also evaluated methods for the sequential extraction of proteins, metabolites, and lipids [4,16,17,18,19,20]. While the use of chloroform/MeOH/MTBE mixtures for metabolomic and lipidomic sample preparation is well-established, the reproducibility of proteomics data is not ideal when proteins are simultaneously extracted [21,22,23,24]. In contrast to existing studies that have predominantly focused on quantitative investigations such as the number of proteins identified to evaluate technical performance or demonstrated improvements in modified protocols, our research examined a more comprehensive analysis of various sample preparation methods. We not only assessed the effectiveness of these methods but also provide detailed results that support selecting the most suitable pre-processing approach, considering variables in actual experimental steps, such as human error and batch effects. For this, we applied and examined various methods including the commonly used 80% methanol (MeOH)-based technique, the chloroform–methanol-water extraction method, and, additionally, an MTBE-based method. These diverse approaches were investigated for their phase-specific differences. Additionally, we evaluated the impact of the metabolite–lipid extraction process on resuspended proteins, contrasting the commonly used FASP and S-Trap methods. This comparative analysis not only highlighted the biological complexities in various experimental approaches but also presented specific insights into different protein pathways and cellular components. Our findings may guide researchers in selecting the appropriate pre-processing method based on the type of multi-omics experiment, in addition to serving as a basis for an optimized protocol suitable for multi-omics experiments, simplifying the analysis of complex biological mechanisms and enhancing research depth and precision.

## 2. Materials and Methods

To determine the proteomic characteristics according to the extraction method, the protein samples obtained after extraction using the three methods (MeOH extraction, chloroform/MeOH-based method, and MTBE-based method) were divided into equal amounts. For proteomics, 18 samples were digested using FASP and S-Trap methods. In addition, nine samples were processed to measure the characteristics of metabolites and lipids according to the extraction method, and 36 raw files were obtained.

### 2.1. Materials and Chemicals

High-performance liquid chromatography (HPLC)-grade MeOH, H_2_O, ACN, and 2-propanol were purchased from JT Baker (Philipsburg, NJ, USA). Ammonium bicarbonate (ABC), formic acid (FA), phosphoric acid, dithiothreitol (DTT), sodium dodecyl sulfate (SDS), triethylammonium bicarbonate (TEAB), urea, iodoacetamide (IAA), chloroform, and ammonium formate (AF) were purchased from Sigma-Aldrich (St. Louis, MO, USA). MTBE was purchased from Merck (Milan, Italy), and tris (2-carboxyethyl) phosphine (TCEP) was obtained from Thermo Fisher Scientific (Rockford, IL, USA). Sequencing-grade modified trypsin was obtained from Promega (Madison, WI, USA). The S-Trap devices were purchased from Protifi (Huntington, NY, USA). A 30 kDa Microcon filtration device (Millipore, Ultracel YM-30, Billerica, MA, USA) was used for protein digestion following FASP. Microspin C18 columns were purchased from Harvard Apparatus (Holliston, MA, USA).

### 2.2. Cell Lysis

**Figure 1:**
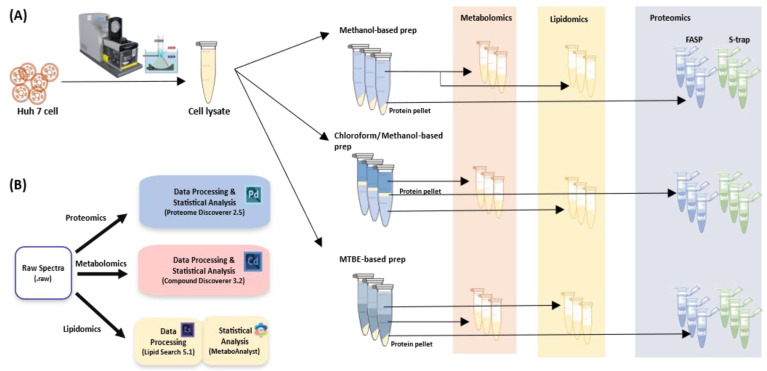
Experimental workflow of comparison of MS-based multi-omics method for cell lysate. (**A**) Schematic picture of the overall experimental design. (**B**) Data processing and statistical analysis of each omics dataset.

Huh7 cells were obtained from American Type Cell Culture (Manassas, VA, USA). Cultures were maintained at 37 °C in a humidified atmosphere containing 5% CO_2_. The cells were cultured in Dulbecco’s modified Eagle’s medium containing 10% fetal bovine serum, 2 mM L-glutamine, and 100 U/mL penicillin/streptomycin. The Huh-7 cell (human) pellets were mixed with 1 mL of lysis solution (8M Urea, 0.1 M Tris-HCl buffer, pH 8.5) and 40 µL of protease inhibitor cocktail (stock solution, 25X) in glass tubes. Cells were lysed using a Covaris S2 focused ultrasonicator (Covaris, Woburn, MA, USA) for 20 min. The overall experimental workflow is illustrated in Figure 1.

### 2.3. Procedure—Proteomics

Protein pellets obtained from the three metabolite/lipid extraction methods were dried and stored at −80 °C. The protein pellets were reconstituted by adding 2% SDS and 60 mM Tris pH = 6.8. To completely dissolve the pellets, sonication was performed in an ultrasound bath (Branson 3510; Branson Ultrasonic Corporation, Brookfield, CT, USA), followed by vortexing several times. The total protein concentration of all samples was determined using bicinchoninic acid (BCA) assay with a Pierce BCA protein assay kit (Thermo Fisher Scientific, Rockford, IL, USA).

#### 2.3.1. FASP Method

First, 8M urea was added to the sample to adjust the volume to 100 µL. Then, 500 mM of TCEP (final concentration: 5 mM) was added to the tubes. After mixing for 1 min at 600 rpm and 37 °C in a thermomixer and incubating at 30 min, 300 rpm, and 37 °C, the sample was transferred to a YM-30 filter and centrifuged at 14,000× *g* for 15 min at 20 °C. A total of 100 µL of 50 mM IAA solution was added, mixed for 1 min at 600 rpm in a thermal mixer, and incubated at 25 °C for 1 h without mixing (dark condition). The YM-30 filter was centrifuged at 14,000× *g* for 15 min at 20 °C. Then, 100 µL of UA was added to the filter unit and centrifuged at 14,000× *g* for 15 min and at 20 °C. Next, 200 µL of 50 mM ABC was added to the sample with trypsin (an enzyme-to-protein ratio of 1:50). The tube was sealed with parafilm, and the filter unit was incubated at 37 °C at 18 h. The filter unit was then transferred to a new collection tube and centrifuged at 14,000× *g* for 10 min. Then, 40 µL of 50 mM ABC centrifuge was added to the filter unit for 10 min at 14,000 g in duplicate at 20 °C. The eluate was acidified with 10–20 µL of formic acid (to make pH 2–3). The collection tubes were dried and desalted using a Micro C18 spin column.

#### 2.3.2. S-Trap Method

The reconstituted protein solution was added to 25 µL SDS (5% SDS, 50 mM TEAB pH 7.55). Samples were clarified by centrifugation for 8 min at 13,000× *g*. Proteins were reduced with TCEP (37 °C, 30 min) and alkylated with IAA (25 °C, 30 min, in the dark). Each tube added 5 µL 12% phosphoric acid to the lysate, and 350 µL of S-Trap binding buffer (90% MeOH, 100 mM final TEAB, pH 7.1) was added to the acidified lysate. The acidified lysate/S-Trap buffer mix was loaded onto an S-Trap filter. The sample tube was centrifuged at 4000× *g* until all solutions had passed through. The tubes were washed 3 times by centrifugation with 400 µL S-Trap binding buffer. Then, 125 μL (50 mM ABC, pH 8) of trypsin (an enzyme-to-protein ratio of 1:50) was added to the protein trap. The spin column was sealed, closed, and incubated at 37 °C overnight. Peptides were eluted by centrifugation at 4000× *g* by adding 80 µL of 50 mM ABC and 0.2% aqueous formic acid solution. Hydrophobic peptides were eluted with 80 µL 50% ACN and 0.2% formic acid. The peptides were dried and desalted using a Micro C18 spin column.

### 2.4. Procedure—Metabolomics/Lipidomics

Through each of the methods below, dried extract for metabolomics was reconstituted in 100 μL of 0.1% formic acid in H_2_O. Others for lipidomics analysis were dissolved in 100 μL of solvent A/solvent B (2:1 *v*/*v*). Solvent A consisted of ACN/MeOH/H_2_O (19:19:2, *v*/*v*/*v*) + 20 mmol/L AF + 0.1% (*v*/*v*) formic acid, whereas solvent B consisted of 2-propanol + 20 mmol/L AF + 0.1% (*v*/*v*) formic acid.

#### 2.4.1. 80% MeOH Method

Cell lysis samples were thawed on ice for 10 min, and 80% MeOH was added. The resulting mixture was vortexed and centrifuged at 2000 rpm for 10 min to pellet the protein and cellular contents. The supernatant was transferred to a new tube, and the contents were dried for metabolomic and lipidomic analyses. Protein pellets were collected for proteomic analysis.

#### 2.4.2. Chloroform/MeOH-Based Method

Chloroform: MeOH (2:1, *v*/*v*) was added to each sample in a CHCl3: MeOH:H_2_O ratio of 8:4:3 (*v*/*v*/*v*). Ice-cold methanol and chloroform were directly added to the samples. The suspension was occasionally vortexed for physical mixing, and the sample was incubated on ice for 30 min. After the addition of H_2_O, which was used to separate the aqueous and organic layers, the suspension was incubated on ice for 10 min. Samples were centrifuged at 2000 rpm for 5 min at 4 °C. The lower-phase (organic) layer was transferred to a new tube, centrifuged at 2000 rpm for 5 min at 4 °C, and collected for lipid analysis. The extract was then dried. The upper (aqueous) layer was centrifuged at 2000 rpm for 5 min at 4 °C and collected for metabolite analyses. The extract was then dried. The middle layer (protein pellet) was collected for proteomic analysis.

#### 2.4.3. MTBE-Based Method

MeOH and MTBE (3:10 *v*/*v*) were added to each sample. The samples were incubated on ice and subjected to orbital shaking for 1 h. After the addition of H_2_O (MTBE/MeOH/H_2_O, 10:3:2.5, *v*/*v*/*v*), the mixture was incubated on ice for 10 min and centrifuged at 2000 rpm for 10 min. The aqueous layer was centrifuged at 2000 rpm for 5 min at 4 °C and collected for metabolite analysis. The lower layer (protein pellet) was collected for proteomic analysis. The organic (upper phase) layer was collected, centrifuged at 2000 rpm for 5 min at 4 °C, and collected for lipid analysis. The extract was then dried.

### 2.5. LC-MS Analysis

Proteomics samples were analyzed using a nano-LC/MS system. Peptide mixtures were analyzed using a Dionex Ultimate 3000 HPLC system coupled with a Q-Exactive Hybrid Quadrupole-Orbitrap MS (Thermo Fisher Scientific, Waltham, MA, USA). Metabolite-/lipid-containing solutions were processed using UHPLC/Q-Exactive. Metabolomics and lipidomics were analyzed using an Agilent Technologies 1290 UHPLC system (Agilent Technologies, Santa Clara, CA, USA) coupled to a Q-Exactive (Thermo Fisher Scientific, Waltham, MA, USA).

#### 2.5.1. Chromatographic/Mass Spec Conditions—Proteomics

Acclaim PepMap 100 C18 nano-trap column (75 μm × 2 cm, 3 μm particles, 100 Å pores, Thermo Fisher Scientific) and Acclaim PepMap C18 100A RSLC nano-column (75 μm × 50 cm, 2 μm particles, 100 Å pores, Thermo Fisher Scientific) were used to separate the peptide mixtures. The solvent consisted of solvent A (0.1% FA in H_2_O) and solvent B (0.1% FA in 80% ACN). The flow rate was maintained at 300 nL/min. The gradient set up for solvent B was as follows (%B): 0–14 min, 4%; 14–75 min, 4–20%; 75–145 min, 20–50%; 145–147 min, 50–96%; 147-160 min, 96%; 160–161 min, 96–4%; and 161–185 min, 4%.The nano-electrospray ionization source was operated in the positive mode with a spray voltage of 2.0 kV. The capillary temperature was 320 °C. The isolation width was ± 2 *m*/*z*, and the scan range was 400 to 2000 *m*/*z*. The resolutions of the full-MS and MS/MS scans at 400 *m*/*z* were 70,000 and 17,500, respectively.

#### 2.5.2. Chromatographic/Mass Spec Conditions—Metabolomics

Chromatographic separation of all the extracts was carried out on Agilent ZORBAX rapid-resolution high-definition stablebond SB-Aq (2.1 × 150 mm, 1.8 μm) column using the 1290 HPLC system. The mobile phases were made up of 0.1% formic acid in H_2_O (solvent A) and 0.1% formic acid in 80% ACN (solvent B). The flow rate was 200μL/min. The gradient elution was 0–5 min, 2.5% B (linear); 5–34 min, 2.5–12.5% B; 34–45 min, 12.5–25% B; 45–56 min, 25–37.5% B; 56–56.1 min, 37.5–80% B; 56.1–70 min, 80% B; 70–70.1 min, 80–2.5% B; 70.1–90 min, 2.5% B. Mass spectrometric analyses were performed in positive electrospray ionization mode. The resolution of the full-MS and MS/MS scans was 70,000 and 17,500 (at 400 *m*/*z*). The scan range was 150–2000 *m*/*z* and 2 × 10^5^ of AGC target, and maximum IT was 250 ms, with ±2 *m*/*z* of isolation width and NCE for dd-MS2 of 30%.

#### 2.5.3. Chromatographic/Mass Spec Conditions—Lipidomics

The used column was a Hypersil GOLD column (2.1 × 100 mm ID; 1.9 μm, Thermo Science). Mobile phases consisted of sol A (ACN/MeOH/H_2_O, 19:19:2, *v*/*v*/*v*) + 20 mmol/L AF + 0.1% (*v*/*v*) formic acid) and sol B (2-propanol + 20 mmol/L AF +0.1% (*v*/*v*) formic acid). The gradient set up for solvent B was as follows (%B): 0–5 min, 5%; 5–15 min, 5–30%; 15–22 min, 30–90%; 22–25 min, 90%; 25–26 min, 90–5%; and 26–30 min, 5%. The flow rate was 150 μL/min, and the total run time was 30 min. The volume of sample injection was 5 μL for each run. The parameters of the operating conditions were as follows: scan range, 150–2000 *m*/*z*. The resolution of the full MS and MS/MS scans were 70,000 and 35,000, respectively.

### 2.6. Data Processing/Statistical Analysis

Xcalibur 3.1 software (Thermo Fisher Scientific, Waltham, MA, USA) was used for data acquisition, raw file generation, inspection of the chromatographic profile, and sample confirmation.

#### 2.6.1. Proteomics

Protein identification and label-free quantification were performed using Proteome Discoverer 2.5.0.400 software [25] (Thermo Fisher Scientific, Waltham, MA, USA), with a maximum of two missed cleavages by trypsin, and 10 ppm and 0.02 Da tolerances of precursor ion masses and fragment ion mass, respectively. The modification options were carbamidomethylation of cysteine (+57.012 Da), methionine oxidation (+15.995 Da), and carbamylation of the protein in the N-terminus (+43.0006 Da). Peak lists were generated using a precursor signal-to-noise ratio of 1.5. A decoy database search was performed to determine the peptide false discovery rate (FDR) using the Target Decoy PSM (peptide-spectrum matches) validator module. Protein identification was filtered by unique peptide ≥ 2. Quantification was performed using a Quantification Module, and normalization was performed to determine the total peptide amount. A 1% peptide FDR threshold was used. Supplementary Method S1 shows the entire workflow of Proteome Discoverer. The H. sapiens protein list was obtained from Uniprot (released on february 2015). Also, raw MS/MS data files were converted. mzXML format was converted using MSConvert. Chromatogram data were included in Figure S1. KEGG pathway [26] and Gene Oncology [27] analyses were performed, with their biological function based on the DAVID database (FDR < 0.05) [28]. Significant GO terms and KEGG pathways were identified with a *p*-value of < 0.05. Proteomic data were deposited to the ProteomeXchange Consortium PRIDE partner repository with the identifier PXD047010 [29].

#### 2.6.2. Metabolomics

Compound Discoverer 3.2.0.421 (Thermo Fisher Scientific, Waltham, MA, USA). The workflow for untargeted metabolomics was used for the retention time alignment and compound identification. McCloud was used to annotate compounds at the MS/MS level. The ChemSpider, Human Metabolome Database, and KEGG databases were used to annotate features based on exact mass using the Compound Discoverer internal database. The chemical background was removed using a blank file. Supplementary Method S2 describes the entire workflow of Compound Discoverer. In the obtained dataset, identification was matched with levels 2 and 3 by filtering using annotations implemented in the Metabolomics Standards Initiative of the Metabolomics Society (Figure S2) [30]. Level 2 matched with a mass tolerance of 5 ppm/with a fragmentation score over 80 in the mzCold database, and level 3 matched with a mass tolerance of 5 ppm in an MS 1 database (Chemspider). Metabolomics data was assessed the quality and reproducibility by RSD histograms (Figure S3). And, chromatogram data were included in Figure S4.

#### 2.6.3. Lipidomics

Lipids were identified and quantified using LipidSearch software v5.1.6 (Thermo Fisher Scientific, Waltham, MA, USA). This allowed lipid identification based on an MS/MS match. The mass tolerances of the precursor and fragment were set to 5 ppm. Grades A, B, C, and D were used for the ID quality filter (Figure S5). All lipid classes were used for identification. Chromatogram data were included in Figure S6. In addition, lipids with odd-numbered fatty chains, generally known as bacterial fatty acids, were filtered out to accurately consider animal lipids [31,32]. Principal component analysis (PCA) and partial least squares discriminant analysis (PLS-DA) were performed using MetaboAnalyst (ver. 5.0) [33]. All raw metabolomics and lipidomics MS data and search results were deposited in the MetaboLights repository with the identifier MTBLS3370 [34].

## 3. Results

### 3.1. Proteomics

**Figure 2:**
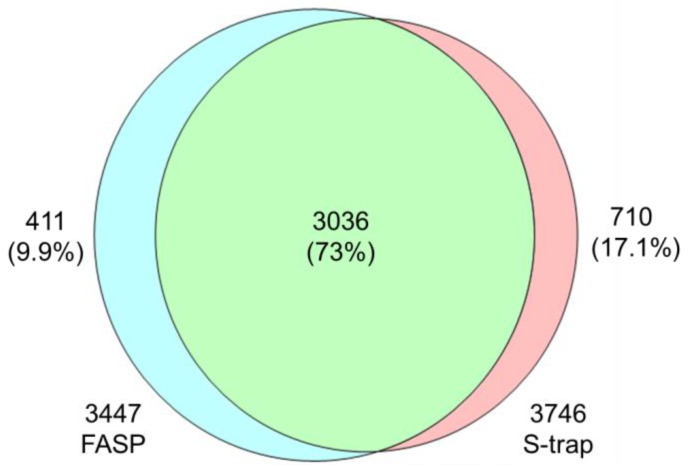
Venn diagram of FASP and S-Trap proteomics analysis.

Qualitative analysis of each digestion method revealed that FASP identified 4462 proteins, whereas S-Trap identified 4719. Following the imputation of missing values to enhance the statistical analysis, the total numbers were adjusted to 3447 and 3746 proteins for FASP and S-Trap, respectively (Figure 2). An evaluation of the effectiveness of the tryptic digestion (Figure S7) revealed 1–2 missing cleavages in approximately 13% and 17% of peptides in FASP and S-Trap, respectively.

#### 3.1.1. Comparison of FASP and S-Trap Digestion Methods Using KEGG Analysis

A KEGG pathway analysis was performed to characterize the unique proteins identified using FASP and S-Trap and to assess the effectiveness of each method in the identification of specific protein types (Table S1). Accordingly, 411 and 710 proteins were specifically identified using FASP and S-Trap, respectively. FASP was found to be more specific in identifying proteins related to cellular infections and internal signal transduction, whereas S-Trap was more specific for proteins related to the structure, function, and neurological diseases (Table 1).

#### 3.1.2. Comparison of Profiling Characteristics of FASP and S-Trap Methods

Cellular components are structures occupying physical spaces within cells and, thereby, exhibit distinctive physical and chemical properties. Therefore, we conducted a comparative Gene Oncology analysis of the cellular components of proteins specifically identified upon the application of each method (Table 2). Accordingly, FASP may be more useful for studies focusing on cellular organelles and membrane-related proteins, whereas S-Trap may assist studies focused on nuclear RNA processing, transcriptional regulation, cell cytoskeleton, and cellular-movement-related proteins.

Differences between the two methods were also observed in identification proteins related to cellular organelles and nuclei. FASP was found to extract proteins associated with cellular organelles (such as endosomes, lysosomes, and the Golgi network) and complexes such as SNARE and HOPS, whereas S-Trap demonstrated a propensity for proteins related to the cell nucleus, cell membrane, nuclear speck, and nuclear envelope. In particular, S-Trap exhibited a more effective identification of RNA processing-related complexes (spliceosomal complexes), likely due to the better preservation of RNA–protein interactions.

We also examined the differences in the identified membrane and cytoskeletal proteins. FASP demonstrated better detection of various membrane proteins, including those from ER and mitochondrial membranes. In contrast, S-Trap demonstrated superior performance in identifying proteins related to the cellular cytoskeleton (actin cytoskeleton, stress fibers, etc.). Notably, there were significant quantitative differences in certain proteins between the two sample preparation methods. Specifically, proteins such as H3C15 (log2(FC) = 2.08), NUDT9 (log2(FC) = −1.00), and NUFIP2 (log2(FC) = −1.08) showed differential expression (Table S2). Although only some proteins exhibited these differences, they suggest that the optimization of quantitative analysis might be achievable by simply altering the method for specific protein types, necessitating further investigation.

### 3.2. Metabolomics

Beyond proteins, the identification of metabolites and lipids is also important to understand the mechanisms of molecular networks mediating cellular responses [35]. Indeed, protein data alone may not comprehensively reflect intracellular protein activity. To this end, other activities related to protein activities, such as metabolite synthesis, may be characterized by combining information on metabolites and lipids [36]. Moreover, concentrations of metabolites can be estimated based on reconstituted protein concentrations [37].

#### 3.2.1. VIP Score upon PLS-DA Classification

**Figure 3:**
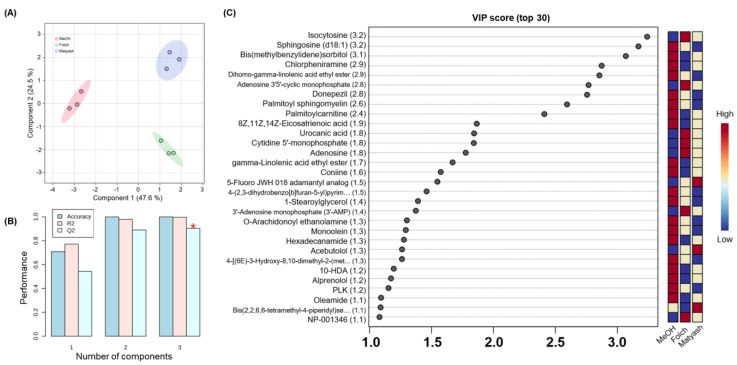
Partial least squares discriminant analysis (PLS-DA) score plots and variable importance of projection (VIP) score plots: (**A**) PLS−DA scores plot of Huh−7 metabolite for three different methods; (**B**) cross−validation data showed cumulative values of R^2^ and Q^2^ (* = the highest value for performance measure); (**C**) the top 30 metabolites with highest VIP scores in tissues for three different methods.

PLS-DA, a supervised clustering method, was applied to identify differences between the results obtained from MeOH extraction and the Folch and Matyash methods (Figure 3A). Thus, the extent to which each variable contributes to group classification was identified. The PLS-DA results demonstrated that all methods displayed significant separation, with a 47.6% contribution for component 1 and 24.5% for component 2. The model also showed varying levels of fit with validated predictability. The cumulative R2 and Q2 values were 0.997 and 0.904 (Figure 3B), respectively. To identify the metabolites that most significantly contributed to the separation of groups, VIP scores were computed (Figure 3C). Identified metabolites included organic compounds (e.g., urocanic acid, iso cytosine, coniine, and bis(methyl benzylidene)sorbitol), nucleotides and related compounds (e.g., cytidine 5′-monophosphate (hydrate), adenosine 3′5′-cyclic monophosphate, adenosine, adenine, and 3′-adenosine monophosphate), fatty acids and derivatives (e.g., sphingosine (d18:1), palmitoylcarnitine, palmitoyl sphingomyelin, oleamide, O-arachidonoyl ethanolamine, monoolein, hexadecanamide, gamma-Linolenic acid ethyl ester, dihomo-gamma-linolenic acid ethyl ester, 8Z,11Z,14Z-eicosatrienoic acid, 1-stearoylglycerol, and 10-HDA), and drugs (e.g., donepezil, chlorpheniramine, alprenolol, and acebutolol), as shown in Figure 3.

#### 3.2.2. ANOVA

The ANOVA results indicated significantly higher concentrations of lipid-related compounds in the MeOH extraction group (Table S3). These compounds included palmitoylcarnitine, oleamide, dihomo-gamma-linolenic acid ethyl ester, 1-stearoylglycerol, 8Z,11Z,14Z-eicosatrienoic acid, monoolein, sphingosine (d18:1), gamma-linolenic acid ethyl ester, hexadecanamide, ethyl myristate, and (±)15-HETE. These findings suggest that MeOH extraction is suitable for extracting a multitude of amphiphilic metabolites with mixed polarities in a single-phase extraction procedure [38]. To this end, MeOH extraction offers a simplified and convenient approach, minimizing the factors to be considered in the experimental steps.

Two-phase extraction via the Folch and Matyash methods yielded significant levels of hydrophilic compounds, particularly those related to nucleotides, such as adenosine-related compounds. These compounds included isocytosine, adenosine 3′5′-cyclic monophosphate, cytidine 5′-diphosphocholine, cytidine 5′-monophosphate (hydrate), adenosine, nicotinamide adenine dinucleotide phosphate (NADP+), cyclic adenosine diphosphate-ribose, adenosine diphosphate, adenosine 5′-monophosphate, adenine, and 3′-adenosine monophosphate.

### 3.3. Lipidomics

**Figure 4:**
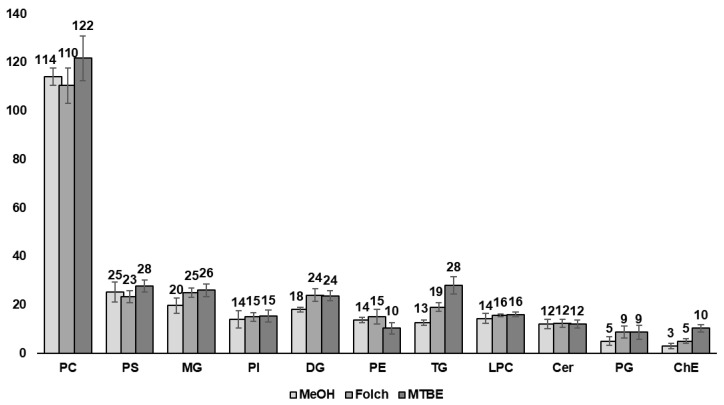
Bar graph for each lipid class according to each sample preparation method.

Finally, a lipidomics analysis was conducted to further understand the features revealed during the metabolomics analysis. The results were based on the commonly used ESI positive mode, which is consistent with other omics analyses. Eleven lipid classes—PC, PS, MG, PI, DG, PE, TG, LPC, Cer, PG, and ChE—were included in the analysis (Figure 4 and Table S4).

The PC class was the most abundant among all groups, accounting for approximately 40% of the identified lipid classes. No significant differences were observed between classes with a high number of identified lipids, suggesting that the lipidomic results only primarily present the distinct molecular characteristics of specific lipid classes. These results implied that the differences in qualitative analysis are more affected by sample pre-processing than by the performance of the analytical instrument. Thus, five specific classes, ChE, TG, PG, DG, and MG, were mainly identified through the two-phase extraction method and exhibited at least a 1.3-fold change in the number of identified lipids compared to the MeOH extraction, presenting the most significant differences in ChE and TG. ChEs, a class of lipids with strong hydrophobic properties, are indeed rarely detected in MeOH extractions. Furthermore, reduced lipid yields were observed in the TG, DG, and MG classes, confirming a gradual increase in the fold-change as lipid hydrophobicity increased.

## 4. Discussion

In our efforts to exclusively consider differences only attributed to the digestion method, we endeavored to control numerous variables. Of them, we verified through existing research that protein loss occurring during the precipitation step throughout the entire experiment does not exert a significant impact on our target aspects [9]. The higher number of proteins identified as S-Trap than FASP may be due to the relatively higher number of missed cleavage peptides. Then, scatter plots using Pearson correlation (Figure S8) were applied to assess the biological reproducibility. The scatter plots confirmed good reproducibility, as evidenced by the high correlation coefficient values among samples within each respective group. Subsequently, we aimed to visualize the distribution histograms of identified proteins based on their isoelectric points and molecular weights for both the FASP and S-Trap digestion methods (Figures S9 and S10). Upon a detailed examination of the distribution data for both digestion methods, similar patterns without any distinct trends were observed in all histograms. Thus, this result supported factors other than digestion efficiency being well-controlled in this study. This result was highlighted by the previous research presented in the comparative study between FASP and S-Trap [39]. The differences between both methods in this study lay in the use of urea and the addition of the SDS detergent. The unique protein identification results, with 9.9% from FASP and 17.1% from S-Trap, were influenced by the variations in these used reagents. Detergents are important factors affecting protein activity, considering their solubilization strength and denaturation severity [40]. Also, the incomplete removal of detergents during the preparation steps can lead to issues such as ion suppression and reduced trypsin efficiency during MS analysis [40,41,42,43]. Therefore, we applied the reagents to minimize errors arising from the preparation steps’ efficiency and other factors based on previous research [39].

The proteins identified through FASP were found to be involved in pathways related to bacterial infections, immune responses, and signal transduction, including Shigellosis, Salmonella infections, Endocytosis, the NOD-like receptor signaling pathway, and SNARE interactions in vesicular transport. S-Trap were also found to be more effective at identifying proteins associated with pathways such as retrograde endocannabinoid signaling, spliceosomes, nucleocytoplasmic transport, basal transcription factors, and pathways of neurodegeneration and multiple diseases. These results indicated that FASP digestion effectively identifies proteins involved in bacterial infection and membrane proteins and, thus, enables a more detailed analysis of the interactions between bacteria and host cells during infection. In contrast, S-Trap did not distinctly identify any pathways associated with bacterial infections. However, the S-Trap method demonstrated a particular strength in identifying RNA-related proteins, enabling effective identification for further analysis of these proteins. In GO analysis, we compared the ability of both methods to reveal proteins related to extracellular and intracellular signaling. Here, FASP was found to be more effective at identifying proteins involved in the export of substances, such as exosomes, to the extracellular space. In contrast, the S-Trap method was effective at identifying proteins related to intracellular signal processing. In conclusion, the physical differences between the two digestion methods, specifically the spatial distribution of proteins and reaction components, may introduce biases in protein identification.

Particularly, of the top nine substances with VIP scores exceeding 2, seven presented high quantitative values during MeOH extraction (Figure 3C). Among these seven compounds, four were fatty-acid-related substances (i.e., sphingosine (d18:1), dihomo-gamma-linolenic acid ethyl ester, palmitoyl sphingomyelin, and palmitoylcarnitine), while two included the strongly hydrophobic drugs donepezil [44] and chlorpheniramine [45], which primarily exhibit hydrophobic interactions. These findings suggest that advantages such as efficiency, rapid analysis, and the analysis of a broad range of substances in a single run may be beneficial for the profiling of hydrophilic metabolites, compared to multi-step processes, including lipid extraction procedures. Furthermore, the significance of considering sample characteristics when arriving at meaningful conclusions from experiments was also emphasized. Our results suggested that the lack of separation of hydrophobic and hydrophilic substances during pre-preparation for single-phase extraction could potentially lead to significant lipid interference. Single-phase extraction may, therefore, result in a limited analysis of relatively hydrophilic or polar metabolites under the same analytical conditions and durations. In addition, extraction with polar solvents, such as chloroform, may be effective for isolating molecules with amphiphilic properties leaning toward weak hydrophilicity. Certain amphiphilic compounds, such as guanine, were only identified at higher level values when the chloroform-based Folch method was used.

In summary, the qualitative analysis showed that MeOH extraction yields were comparable to those obtained from conventional lipid extraction methods, such as the Folch and Matyash methods, except for neutral lipid classes. Therefore, MeOH extraction may offer a balance between time efficiency and the quality of the results, especially when investigating polar lipids such as PC. Furthermore, compared to the two-phase methods such as Folch and MTBE often used in lipidomics, MeOH extraction stands out for its simplicity and high reproducibility. Moreover, the ability of MeOH to disrupt hydrogen bonds and electrostatic networks between lipid and protein molecules underscores its suitability for proteomics research and integrative experiments [24].

In contrast, the extraction of neutral lipids, such as TG, is more effective with hydrophobic organic solvents, like those used in the two-phase extraction methods applied here [46]. Notably, the Matyash method was particularly effective for extracting high amounts of TG and ChE lipids. In untargeted lipidomic analyses, the choice of solvent mixtures is crucial for efficiently extracting a wide range of lipid classes. These mixtures include polar solvents capable of separating lipids from cell membranes and lipoproteins as well as non-polar solvents for isolating neutral lipids. Two-phase extraction allows for the expansion of the range of analytes per sample extraction by separating and analyzing substances based on their polarity, which are distributed into both aqueous and organic layers. Hence, a more comprehensive understanding of complex biological systems can be obtained.

In addition to the chemical characteristics, physical properties can also affect extraction results, as evidenced by the formation of a layer at the boundary between the aqueous and organic phases during the pre-processing step of the Folch method. This boundary layer includes an insoluble matrix containing proteins, which raises concerns about protein recovery in proteomic experiments, the risk of ion suppression during the ionization step, and the potential for clogging issues in LC systems [20]. Furthermore, the high density and viscosity of chloroform limit its effectiveness in impurity removal via centrifugation. Several studies also highlighted the poor reproducibility of protein recovery and the reduced recovery rates for hydrophilic metabolites [47].

In contrast, the Matyash method, which utilizes MTBE, forms a lipid-rich organic phase above the aqueous phase. This method allows for easier protein identification into pellets through centrifugation, offering advantages for proteomic experiments [24]. However, previous research also suggested that the high volatility of MTBE may affect reproducibility [20]. In conclusion, our findings highlighted the need for novel pre-processing methods, including extraction techniques, to optimize non-specific shotgun analysis in profiling experiments performed with limited prior information.

## 5. Conclusions

Our study applies various multi-omics pre-processing methods to a hepatocyte cell line, comparing the results to identify specific differences among sample pre-processing methods. We offer in-depth information by analyzing these differences from various aspects, such as cellular components, and presenting detailed findings not previously investigated in previous studies. These results have the potential to contribute to the development of biomolecular profiling strategies for researchers. Our research, thus, opens a new avenue through which proteomics and metabolomics can contribute to future research in areas ranging from basic biology to modern medicine and drug development.

## Figures and Tables

**Table 1:** KEGG pathway analysis performed to characterize the unique proteins identified using FASP and S-Trap methods.

	Term	*p*-Value	Gene Name
FASP	Shigellosis	3.00 × 10^−6^	BECN1, GSK3B, ROCK1, PXN, UBE2D1, TRAF2, WIPI2, MAPK14, ATG12, NFKB1, MYL12B, SEPTIN8, RPTOR, MAPK9, CYTH2, H3C15, H3-4, RRAGB, PLCG1, H3-3A
	Non-alcoholic fatty liver disease	2.01 × 10^−4^	GSK3B, UQCRB, INSR, NDUFA10, TRAF2, COX6C, SDHA, MAPK14, NFKB1, MAPK9, NDUFAB1, UQCRFS1, UQCRC2
	Thermogenesis	7.91 × 10^−4^	COA3, UQCRB, NDUFA10, ACSL4, COX6C, SDHA, MAPK14, ARID1B, RPTOR, RPS6KA3, CREB1, NDUFAB1, COX11, UQCRFS1, UQCRC2
	Alzheimer’s disease	1.11 × 10^−3^	BECN1, RTN3, GSK3B, FZD2, UQCRB, APAF1, INSR, NDUFA10, TRAF2, WIPI2, COX6C, SDHA, NFKB1, MAPK9, PPP3R1, PSMA4, NDUFAB1, UQCRFS1, CTNNB1, UQCRC2
	RNA degradation	2.02 × 10^−3^	HSPA9, EXOSC5, CNOT7, EXOSC9, CNOT10, PABPC1, EXOSC2, EXOSC1
S-Trap	RNA degradation	7.35 × 10^−4^	HSPA9, CNOT7, EXOSC9, EXOSC8, PARN, PABPC1, NUDT16, ENO3, EXOSC2, EXOSC1, LSM3
	Basal transcription factors	1.36 × 10^−3^	TAF6L, GTF2A2, TAF15, TBP, ERCC2, TAF5, GTF2H3, MNAT1
	Parkinson’s disease	2.58 × 10^−3^	NDUFA8, NDUFB8, MT-ND5, NDUFA6, TUBAL3, APAF1, NDUFB3, HTRA2, ITPR2, SDHC, SDHA, MT-ND2, SLC39A14, MAPK8, PSMA4, NDUFS6, MFN2, PLCG1, UQCRC2, NDUFV2, SLC39A1
	Huntington’s disease	2.80 × 10^−3^	DCTN5, NDUFA8, NDUFB8, MT-ND5, NDUFA6, TBP, TUBAL3, APAF1, NDUFB3, GPX7, SLC1A3, SDHC, SDHA, MT-ND2, AP2A2, MAPK8, PSMA4, NDUFS6, EP300, UQCRC2, NDUFV2, POLR2I, POLR2J
	mRNA surveillance pathway	3.53 × 10^−3^	PPP2CA, CPSF4, FIP1L1, NCBP2, CSTF2T, PPP2R5A, PABPC1, MSI2, BCL2L2-PABPN1, SMG5, SMG6

**Table 2:** GO analysis list performed to characterize the unique proteins identified using FASP and S-Trap methods.

	Term	Count	*p*-Value
FASP	late endosome membrane (GO:0031902)	11	6.02 × 10^−8^
	cytosol (GO:0005829)	68	6.10 × 10^−8^
	Golgi apparatus (GO:0005794)	25	5.77 × 10^−7^
	ubiquitin ligase complex (GO:0000151)	9	8.68 × 10^−7^
	endoplasmic reticulum (GO:0005783)	24	2.52 × 10^−6^
	macromolecular complex (GO:0032991)	18	3.40 × 10^−6^
	intracellular membrane-bounded organelle (GO:0043231)	21	1.16 × 10^−5^
	membrane (GO:0016020)	46	1.61 × 10^−5^
	trans-Golgi network (GO:0005802)	9	4.28 × 10^−5^
	lysosome (GO:0005764)	11	4.87 × 10^−5^
S-Trap	nucleoplasm (GO:0005654)	80	8.39 × 10^−13^
	nuclear speck (GO:0016607)	23	7.65 × 10^−11^
	nuclear membrane (GO:0031965)	18	2.22 × 10^−10^
	centrosome (GO:0005813)	25	3.80 × 10^−10^
	mitochondrial respiratory chain complex I (GO:0005747)	9	1.79 × 10^−8^
	kinetochore (GO:0000776)	13	2.78 × 10^−8^
	cytosol (GO:0005829)	86	5.28 × 10^−8^
	mitochondrial inner membrane (GO:0005743)	19	4.50 × 10^−7^
	nuclear envelope (GO:0005635)	12	2.57 × 10^−6^
	actin cytoskeleton (GO:0015629)	13	5.96 × 10^−6^

## Data Availability

Data are contained within the article or Supplementary Materials.

## Supplementary Materials

The following supporting information can be downloaded at https://www.mdpi.com/article/10.3390/metabo14010034/s1. Table S1: List of identified proteins (label-free quantification) identified by Proteome Discoverer and KEGG–GO analysis; Table S2: Fold change and p-value of Q71DI3, Q9BW91, Q7Z417; Table S3: List of identified VIP score/ANOVAs in each method (metabolomics); Table S4: List of identified lipids in each method; Method S1: Data analysis workflow of Proteome Discoverer; Method S2: Data analysis workflow of Compound Discoverer; Figure S1: Chromatogram of proteomics data of each method.; Figure S2: List of the number of metabolite features, compounds identified as level of confidence 2,3 for each extraction method; Figure S3: Histogram for RSD of each method (metabolite); Figure S4: Chromatogram of metabolomics data of each method; Figure S5: ID-grade graph for each extraction method of lipids identified by lipid search; Figure S6: Chromatogram of lipidomics data of each method; Figure S7: Number of missed cleavages in identified peptides; Figure S8: Correlation scatter plot in proteomics data; Figure S9: Histogram of pI (isoelectric points); Figure S10: Histograms and cumulative distributions of molecular weight (MW).
