# Supplementary material for: Optimizing MS-Based Multi-Omics: Comparative Analysis of Protein, Metabolite, and Lipid Extraction Techniques
Source: Metabolites. 2024 Jan 3;14(1):34. doi: 10.3390/metabo14010034 (PMC10820684; doi:10.3390/metabo14010034)
Supplement: Supplementary file 1 [file metabolites-14-00034-s001.zip › Method S1_Data analysis workflow of Proteome Discoverer.pdf]

Result name: 20201109_Multi_P1-1

Result file: B:₩Data₩multi₩Multi_proteomics₩20201109_Multi_P1-1-(2).pdResult

Description: Result filtered for high confident peptides, with enhanced peptide and protein annotations. Add FASTA file with common contaminants to the Protein Marker node. Quan abunaces are normalized to the same total peptide amount per channel and scaled, so that the average abunce per protein and peptide is 100.

Workflow based on template: CWF_Comprehensive_Enhanced Annotation_LFQ_and_Precursor_Quan

Creation date: 1/17/2021 2:30:35 PM

Created with Discoverer version: 2.5.0.400

The workflow tree:

|-(0) MSF Files

|-(10) Feature Mapper

|-(11) Precursor Ions Quantifier

|-(1) PSM Grouper

|-(2) Peptide Validator

|-(3) Peptide and Protein Filter

|-(4) Protein Scorer

|-(7) Protein FDR Validator

|-(5) Protein Grouping

|-(6) Peptide in Protein Annotation

|-(9) Protein Marker

|-(8) Protein Annotation

Post-processing nodes:

|-(13) Display Settings

|-(14) Data Distributions

Processing node 0: MSF Files

----

1. Storage Settings:

- Spectra to Store: Identified or Quantified

- Feature Traces to Store: All

2. Merging of Identified Peptide and Proteins:

- Merge Mode: Globally by Search Engine Type

3. FASTA Title Line Display:

- Reported FASTA Title Lines: Best match

- Title Line Rule: standard

4. PSM Filters:

- Maximum Delta Cn: 0.05

- Maximum Rank: 0

- Maximum Delta Mass: 0 ppm

Hidden Parameters:

- MSF File(s): C:₩Users₩프로테오믹스₩Desktop₩Data₩multi₩Multi_proteomics₩20201109_Multi_P1-1-(2).msf

----

Processing node 10: Feature Mapper

----

1. Chromatographic Alignment:

- Perform RT Alignment: True

- Maximum RT Shift [min]:   10

- Mass Tolerance: 10 ppm

- Parameter Tuning: Coarse

2. Feature Linking and Mapping:

- RT Tolerance [min]:   0

- Mass Tolerance: 0 ppm

- Min. S/N Threshold: 5

Processing node 11: Precursor Ions Quantifier

----

1. General Quantification Settings:

- Peptides to Use: Unique + Razor

- Consider Protein Groups for Peptide Uniqueness: True

- Use Shared Quan Results: True

- Reject Quan Results with Missing Channels: False

2. Precursor Quantification:

- Precursor Abundance Based On: Intensity
- Min. # Replicate Features [%]: 0

3. Normalization and Scaling:

- Normalization Mode: Total Peptide Amount
- Scaling Mode: On All Average

4. Exclude Peptides from Protein Quantification:

- For Normalization: Use All Peptides
- For Protein Roll-Up: Use All Peptides
- For Pairwise Ratios: Exclude Modified

5. Quan Rollup and Hypothesis Testing:

- Protein Abundance Calculation: Top N Average
- N for Top N: 3
- Protein Ratio Calculation: Pairwise Ratio Based
- Maximum Allowed Fold Change: 100
- Imputation Mode: None
- Hypothesis Test: t-test (Background Based)

6. Quan Ratio Distributions:

- 1st Fold Change Threshold: 2
- 2nd Fold Change Threshold: 4

- 3rd Fold Change Threshold: 6

- 4th Fold Change Threshold: 8

- 5th Fold Change Threshold: 10

Processing node 1: PSM Grouper

----

1. Peptide Group Modifications:

- Site Probability Threshold: 75

Processing node 2: Peptide Validator

----

1. General Validation Settings:

- Validation Mode: Automatic (Control peptide level error rate if possible)

- Target FDR (Strict) for PSMs: 0.01

- Target FDR (Relaxed) for PSMs: 0.05

- Target FDR (Strict) for Peptides: 0.01

- Target FDR (Relaxed) for Peptides: 0.05

2. Specific Validation Settings:

- Validation Based on: q-Value

- Target/Decoy Selection for PSM Level FDR Calculation Based on Score: Automatic

- Reset Confidences for Nodes without Decoy Search (Fixed score thresholds):   False

----

Processing node 3: Peptide and Protein Filter

----

1. Peptide Filters:

- Peptide Confidence At Least: High

- Keep Lower Confident PSMs: False

- Minimum Peptide Length: 6

- Remove Peptides Without Protein Reference: False

2. Protein Filters:

- Minimum Number of Peptide Sequences: 1

- Count Only Rank 1 Peptides: False

- Count Peptides Only for Top Scored Protein: False

Processing node 4: Protein Scorer

No parameters

Processing node 7: Protein FDR Validator

----

1. Confidence Thresholds:

- Target FDR (Strict):   0.01

- Target FDR (Relaxed):   0.05

Processing node 5: Protein Grouping

----

1. Protein Grouping:

- Apply strict parsimony principle: True

Processing node 6: Peptide in Protein Annotation

----

1. Flanking Residues:

- Annotate Flanking Residues of the Peptide: True

- Number Flanking Residues in Connection Tables: 1

2. Modifications in Peptide:

- Protein Modifications Reported: Only for Master Proteins

3. Modifications in Protein:

- Modification Sites Reported: All And Specific

- Minimum PSM Confidence: High

- Report Only PTMs: True

4. Positions in Protein:

- Protein Positions for Peptides: Only for Master Proteins

Processing node 9: Protein Marker

----

1. Contaminant Database:

- Protein Database: HUMAN.fasta

5. Annotate Species:

- As Species Map: False

- As Species Names: False

6. Mark Additional Entities:

- Annotation Groups: False

- Pathway Groups: False

- Modification Sites: True

- Peptide Isoform Groups: True

Processing node 8: Protein Annotation

----

1. Annotation Aspects:

- 1. Aspect: Biological Process

- 2. Aspect: Cellular Component

- 3. Aspect: Molecular Function

- 4. Aspect: None

- 5. Aspect: None

- 6. Aspect: None

2. Annotation/Pathway Groups:

- Protein Database: HUMAN.fasta

Processing node 13: Display Settings

----

1. General:

- Filter Set:

Filter Set MasterProteinFilter.filterset contains the following filters:

Row Filter for TargetProtein:

Master is equal to Master

###

'magellan filter set' 1 'MasterProteinFilter.filterset' FiltersetProperties 1 'LastFileName' 'C:₩Users₩frank.berg₩Desktop₩MasterProteinFilter.filterset' Filter 'TargetProtein' 1 NARY_AND 1 = FilterConditionProperties 1 'NamedComparableFilterCondition/DisplayPropertyHint' 'Master' property 'Thermo.PD.EntityDataFramework.MasterProteinAssessment, Thermo.Magellan.EntityDataFramework' 'IsMasterProtein' constant 'Thermo.PD.EntityDataFramework.MasterProteinAssessment, Thermo.Magellan.EntityDataFramework' 'IsMasterProtein'

Processing node 14: Data Distributions

----

1. ID Distributions (Bottom-up):

- Peptides to Use: Only unique peptides based on protein groups

----

Workflow messages:

01/17/2021 06:51 PM Job Execution: Processing C:₩Users₩프로테오믹스₩Desktop₩Data₩multi₩Multi_proteomics₩20201109_Multi_P1-1-(2).pdResult

01/17/2021 06:51 PM (0) MSF Files: C:₩Users₩프로테오믹스₩Desktop₩Data₩multi₩Multi_proteomics₩20201109_Multi_P1-1-(2).msf

01/17/2021 06:51 PM (0) MSF Files: All 1 files are ready for processing.

01/17/2021 06:51 PM (0) MSF Files: Start transferring results of 1 msf files...

01/17/2021 06:57 PM (0) MSF Files: Transferred 73806 Proteins to result file in 5 min 9 s.

01/17/2021 07:03 PM (0) MSF Files: Transferred 69713 Decoy Proteins to result file in 5 min 58 s.

01/17/2021 07:10 PM (0) MSF Files: Added 875196 Mass Spectra to result file.

01/17/2021 07:17 PM (0) MSF Files: Added 15274543 traces to result file.

01/17/2021 07:17 PM (0) MSF Files: Added 18 Input Files to result file.

01/17/2021 07:24 PM (0) MSF Files: Added 704915 PSMs to result file.

01/17/2021 07:24 PM (0) MSF Files: Added 181305 Decoy PSMs to result file.

01/17/2021 07:25 PM (0) MSF Files: Added 904104 MS/MS Spectrum Info to result file.

01/17/2021 07:26 PM (0) MSF Files: Added 904104 Precursors to result file.

01/17/2021 07:26 PM (0) MSF Files: Added 18 Mass Recalibrations to result file.

01/17/2021 07:26 PM (0) MSF Files: Added 250770 Correction Landmarks to result file.

01/17/2021 07:26 PM (0) MSF Files: Added 54 Specialized Traces to result file.

01/17/2021 07:27 PM (0) MSF Files: Added 4884667 LCMS Features to result file.

01/17/2021 07:30 PM (0) MSF Files: Added 20624556 LCMS Peaks to result file.

01/17/2021 07:59 PM (0) MSF Files: Copying data took 1 h 7 min.

01/17/2021 08:00 PM (0) MSF Files: Finding unique sequences took 28.7 s.

01/17/2021 08:00 PM (0) MSF Files: Filtering proteins took 32.4 s.

01/17/2021 08:03 PM (0) MSF Files: -- Total execution of MSF Files (0) took 1 h 11 min --

01/17/2021 08:03 PM (1) PSM Grouper: Grouping 'PSMs'

01/17/2021 08:06 PM (1) PSM Grouper: Found 157116 Peptide Groups.

01/17/2021 08:06 PM (1) PSM Grouper: Grouping 'Decoy PSMs'

01/17/2021 08:07 PM (1) PSM Grouper: Found 112996 Decoy Peptide Groups.

01/17/2021 08:08 PM (1) PSM Grouper: -- Total execution of PSM Grouper (1) took 4 min 40 s --

01/17/2021 08:08 PM (2) Peptide Validator: Start PSM and Peptide validation in 'Automatic (Control peptide level error rate if possible)' mode...

01/17/2021 08:08 PM (2) Peptide Validator: Updated PSM confidences in 6.2 s.

01/17/2021 08:08 PM (2) Peptide Validator: Use svm score for 'peptide' qvality run.

01/17/2021 08:08 PM (2) Peptide Validator: Updated peptide confidences using qvality in 27.5 s.

01/17/2021 08:08 PM (2) Peptide Validator: Calculating Mascot thresholds.

01/17/2021 08:08 PM (2) Peptide Validator: -- Total execution of Peptide Validator (2) took 34.2 s --

01/17/2021 08:08 PM (3) Peptide and Protein Filter: Filter Peptide Groups

01/17/2021 08:09 PM (3) Peptide and Protein Filter: Filter 43141/157116 Peptide Groups (113975 excluded) and 515656/704915 PSMs (189259 excluded) took 17.4 s

01/17/2021 08:09 PM (3) Peptide and Protein Filter: Filtering Proteins

01/17/2021 08:09 PM (3) Peptide and Protein Filter: Filter 23989/73806 Proteins (49817 excluded) took 26.8 s

01/17/2021 08:09 PM (3) Peptide and Protein Filter: Filter Decoy Peptide Groups

01/17/2021 08:09 PM (3) Peptide and Protein Filter: Filter 428/112996 Decoy Peptide Groups (112568 excluded) and 1811/181305 Decoy PSMs (179494 excluded) took 6.6 s

01/17/2021 08:09 PM (3) Peptide and Protein Filter: Filtering Decoy Proteins

01/17/2021 08:09 PM (3) Peptide and Protein Filter: Filter 1173/69713 Decoy Proteins (68540 excluded) took 3.9 s

01/17/2021 08:12 PM (3) Peptide and Protein Filter: Updating counts took 2 min 3 s.

01/17/2021 08:12 PM (3) Peptide and Protein Filter: -- Total execution of Peptide and Protein Filter (3) took 3 min 10 s --

01/17/2021 08:12 PM (10) Feature Mapper: Extracting features

01/17/2021 08:13 PM (10) Feature Mapper: Extracting LCMS features

01/17/2021 08:15 PM (10) Feature Mapper: Extracting feature groups took 2 min 50 s.

01/17/2021 08:15 PM (10) Feature Mapper: Start aligning 7 files ('F38', 'F41', 'F43', 'F51', 'F53', 'F46', 'F47') to reference file 'F48'...

01/17/2021 08:15 PM (10) Feature Mapper: Start aligning 2 files ('F37', 'F39') to reference file 'F38'...

01/17/2021 08:15 PM (10) Feature Mapper: Start aligning 2 files ('F40', 'F42') to reference file 'F41'...

01/17/2021 08:15 PM (10) Feature Mapper: Start aligning 2 files ('F44', 'F45') to reference file 'F43'...

01/17/2021 08:15 PM (10) Feature Mapper: Start aligning 2 files ('F49', 'F50') to reference file 'F51'...

01/17/2021 08:15 PM (10) Feature Mapper: Start aligning 2 files ('F52', 'F54') to reference file 'F53'...

01/17/2021 08:15 PM (10) Feature Mapper: Calculating RT alignment took 3 min 7 s

01/17/2021 08:15 PM (10) Feature Mapper: Start updating RTs...

01/17/2021 08:16 PM (10) Feature Mapper: Updating RTs of LCMS Features took 37.7 s.

01/17/2021 08:19 PM (10) Feature Mapper: Updating RTs of LCMS Peaks took 2 min 45 s.

01/17/2021 08:19 PM (10) Feature Mapper: Updating RTs of PSMs took 7.1 s.

01/17/2021 08:19 PM (10) Feature Mapper: Updating RTs of MS/MS Spectrum Info took 6.5 s.

01/17/2021 08:19 PM (10) Feature Mapper: -- Performing retention time alignment took 6 min 45 s. --

01/17/2021 08:19 PM (10) Feature Mapper: Extracting feature groups took 0.1 ms.

01/17/2021 08:19 PM (10) Feature Mapper: Start aligning 7 files ('F38', 'F41', 'F43', 'F51', 'F53',

'F46', 'F47') to reference file 'F48'...

01/17/2021 08:19 PM (10) Feature Mapper: Start aligning 2 files ('F37', 'F39') to reference file 'F38'...

01/17/2021 08:19 PM (10) Feature Mapper: Start aligning 2 files ('F40', 'F42') to reference file 'F41'...

01/17/2021 08:19 PM (10) Feature Mapper: Start aligning 2 files ('F44', 'F45') to reference file 'F43'...

01/17/2021 08:19 PM (10) Feature Mapper: Start aligning 2 files ('F49', 'F50') to reference file 'F51'...

01/17/2021 08:19 PM (10) Feature Mapper: Start aligning 2 files ('F52', 'F54') to reference file 'F53'...

01/17/2021 08:20 PM (10) Feature Mapper: Updating m/z values of LCMS Features took 34 s.

01/17/2021 08:24 PM (10) Feature Mapper: Updating m/z values of LCMS Peaks took 3 min 43 s.

01/17/2021 08:24 PM (10) Feature Mapper: Updating m/z values of PSMs took 12 s.

01/17/2021 08:24 PM (10) Feature Mapper: -- Performing mass over charge alignment took 4 min 51 s. --

01/17/2021 08:24 PM (10) Feature Mapper: Extracting feature groups took 0.2 ms.

01/17/2021 08:24 PM (10) Feature Mapper: Start aligning 7 files ('F38', 'F41', 'F43', 'F51', 'F53', 'F46', 'F47') to reference file 'F48'...

01/17/2021 08:24 PM (10) Feature Mapper: Start aligning 2 files ('F37', 'F39') to reference file 'F38'...

01/17/2021 08:24 PM (10) Feature Mapper: Start aligning 2 files ('F40', 'F42') to reference file 'F41'...

01/17/2021 08:24 PM (10) Feature Mapper: Start aligning 2 files ('F44', 'F45') to reference file 'F43'...

01/17/2021 08:24 PM (10) Feature Mapper: Start aligning 2 files ('F49', 'F50') to reference file 'F51'...

01/17/2021 08:24 PM (10) Feature Mapper: Start aligning 2 files ('F52', 'F54') to reference file

'F53'...

01/17/2021 08:25 PM (10) Feature Mapper: Updating area values of LCMS Features took 35.2 s.

01/17/2021 08:28 PM (10) Feature Mapper: Updating area values of LCMS Peaks took 2 min 47 s.

01/17/2021 08:28 PM (10) Feature Mapper: -- Performing abundance alignment took 3 min 44 s. --

01/17/2021 08:28 PM (10) Feature Mapper: Using RT tolerance of 0.56 min and mass tolerance of 7.4 ppm for mapping

01/17/2021 08:28 PM (10) Feature Mapper: Mapping 1 Fraction(s)

01/17/2021 08:29 PM (10) Feature Mapper: Mapping Fraction 1 of 1

01/17/2021 09:09 PM (10) Feature Mapper: Mapping 1 Fraction(s) took 41 min 17 s

01/17/2021 09:09 PM (10) Feature Mapper: -- Performing cross file mapping took 41 min 17 s. --

01/17/2021 09:09 PM (10) Feature Mapper: Storing Top Apex RT for Peptide Groups

01/17/2021 09:10 PM (10) Feature Mapper: Storing Top Apex RT for Peptide Groups took 37 s

01/17/2021 09:10 PM (10) Feature Mapper: -- Total execution of Feature Mapper (10) took 58 min 6 s --

01/17/2021 09:10 PM (4) Protein Scorer: Calculating Coverage and Counts

01/17/2021 09:10 PM (4) Protein Scorer: Calculated counts and coverages in 41.6 s.

01/17/2021 09:10 PM (4) Protein Scorer: Scoring target proteins

01/17/2021 09:12 PM (4) Protein Scorer: Scored 23989 proteins in 2 min 5 s.

01/17/2021 09:12 PM (4) Protein Scorer: Scoring decoy proteins

01/17/2021 09:12 PM (4) Protein Scorer: Scored 1173 decoy proteins in 1.3 s.

01/17/2021 09:12 PM (4) Protein Scorer: -- Total execution of Protein Scorer (4) took 2 min 48 s --

01/17/2021 09:12 PM (7) Protein FDR Validator: Apply confidences based on protein scores of 23989 proteins.

01/17/2021 09:12 PM (7) Protein FDR Validator: Calculating Sum PEP Score for Proteins

01/17/2021 09:13 PM (7) Protein FDR Validator: Calculating Sum PEP Score for Decoy Proteins

01/17/2021 09:13 PM (7) Protein FDR Validator: -- Total execution of Protein FDR Validator (7) took 43.3 s --

01/17/2021 09:14 PM (5) Protein Grouping: Retrieving 5095 protein groups took 3.4 s.

01/17/2021 09:16 PM (5) Protein Grouping: Storing, updating and connecting protein groups, PSMs and peptides took 2 min 6 s.

01/17/2021 09:16 PM (5) Protein Grouping: Check 5095 protein groups.

01/17/2021 09:17 PM (5) Protein Grouping: Applying strict parsimony took 1 min 22 s.

01/17/2021 09:17 PM (5) Protein Grouping: Found 5044 protein groups.

01/17/2021 09:17 PM (5) Protein Grouping: Retrieving 411 decoy protein groups took 6.2 ms.

01/17/2021 09:18 PM (5) Protein Grouping: Storing, updating and connecting protein groups, PSMs and peptides took 14 s.

01/17/2021 09:18 PM (5) Protein Grouping: Found 411 decoy protein groups.

01/17/2021 09:18 PM (5) Protein Grouping: -- Total execution of Protein Grouping (5) took 5 min 7 s --

01/17/2021 09:18 PM (11) Precursor Ions Quantifier: Validating LCMS Features

01/17/2021 09:21 PM (11) Precursor Ions Quantifier: Validating LCMS Features for 8787058 items took 2 min 46 s

01/17/2021 09:21 PM (11) Precursor Ions Quantifier: Validating Consensus Features

01/17/2021 09:23 PM (11) Precursor Ions Quantifier: Validation of quantification results took 4 min 48 s

01/17/2021 09:23 PM (11) Precursor Ions Quantifier: Updating spectrum match areas

01/17/2021 09:23 PM (11) Precursor Ions Quantifier: Classify quan usage of PSMs

01/17/2021 09:23 PM (11) Precursor Ions Quantifier: Classifying Peptide Groups quan usage

01/17/2021 09:24 PM (11) Precursor Ions Quantifier: Classifying Peptide Groups quan usage for 43141 items took 14.9 s

01/17/2021 09:24 PM (11) Precursor Ions Quantifier: Calculating Abundances of Peptide

Groups

01/17/2021 09:24 PM (11) Precursor Ions Quantifier: Classifying Peptide Groups quan usage

01/17/2021 09:24 PM (11) Precursor Ions Quantifier: Counting Razor Peptides

01/17/2021 09:24 PM (11) Precursor Ions Quantifier: Determining TopN Usage

01/17/2021 09:24 PM (11) Precursor Ions Quantifier: Classifying Peptide Groups quan usage for 43141 items took 13.4 s

01/17/2021 09:24 PM (11) Precursor Ions Quantifier: Calculating Abundances of Proteins

01/17/2021 09:24 PM (11) Precursor Ions Quantifier: Start normalization of abundance values...

01/17/2021 09:24 PM (11) Precursor Ions Quantifier: Applying normalization based on 42189 Peptide Groups.

01/17/2021 09:24 PM (11) Precursor Ions Quantifier: Calculating normalization took 23.1 s

01/17/2021 09:24 PM (11) Precursor Ions Quantifier: Calculating normalized Abundances of Peptide Groups

01/17/2021 09:25 PM (11) Precursor Ions Quantifier: Calculating normalized Abundances of Proteins

01/17/2021 09:25 PM (11) Precursor Ions Quantifier: Scale Abundances (Normalized) of Peptide Groups

01/17/2021 09:25 PM (11) Precursor Ions Quantifier: Scale Abundances (Normalized) of Proteins

01/17/2021 09:25 PM (11) Precursor Ions Quantifier: Calculating and applying normalization took 39.1 s

01/17/2021 09:25 PM (11) Precursor Ions Quantifier: Scale Abundances (Grouped) of Peptide Groups

01/17/2021 09:25 PM (11) Precursor Ions Quantifier: Scale Abundances (by Bio. Rep.) of Peptide Groups

01/17/2021 09:25 PM (11) Precursor Ions Quantifier: Scale Abundances (Grouped) of Proteins

01/17/2021 09:25 PM (11) Precursor Ions Quantifier: Scale Abundances (by Bio. Rep.) of Proteins

01/17/2021 09:25 PM (11) Precursor Ions Quantifier: Calculating ratios for Peptide Groups...

01/17/2021 09:25 PM (11) Precursor Ions Quantifier: Calculating ratios for Proteins...

01/17/2021 09:25 PM (11) Precursor Ions Quantifier: Start calculating p-values based on background for Peptide Groups...

01/17/2021 09:58 PM (11) Precursor Ions Quantifier: Calculating p-values based on background took 32 min 58 s

01/17/2021 09:58 PM (11) Precursor Ions Quantifier: Calculating p-values for Peptide Groups took 32 min 59 s.

01/17/2021 09:58 PM (11) Precursor Ions Quantifier: Start calculating p-values based on background for Proteins...

01/17/2021 09:59 PM (11) Precursor Ions Quantifier: Calculating p-values based on background took 1 min 5 s

01/17/2021 09:59 PM (11) Precursor Ions Quantifier: Calculating p-values for Proteins took 1 min 5 s.

01/17/2021 09:59 PM (11) Precursor Ions Quantifier: Quantification took 41 min 9 s.

01/17/2021 10:02 PM (11) Precursor Ions Quantifier: Preparing spectrum visualization took 2 min 11 s.

01/17/2021 10:02 PM (11) Precursor Ions Quantifier: -- Total execution of Precursor Ions Quantifier (11) took 43 min 20 s --

01/17/2021 10:02 PM (6) Peptide in Protein Annotation: Start retrieving flanking residues and positions...

01/17/2021 10:16 PM (6) Peptide in Protein Annotation: Annotated PSMs/peptides for 23989 proteins.

01/17/2021 10:16 PM (6) Peptide in Protein Annotation: Start annotating peptide groups with flanking residues and positions...

01/17/2021 10:16 PM (6) Peptide in Protein Annotation: Annotate Peptide Groups items

01/17/2021 10:17 PM (6) Peptide in Protein Annotation: Start calculating protein coverage...

01/17/2021 10:18 PM (6) Peptide in Protein Annotation: -- Total execution of Peptide in Protein Annotation (6) took 16 min 24 s --

01/17/2021 10:18 PM (8) Protein Annotation: Using annotation server

https://proteinannotationservice.thermofisher.com/.

01/17/2021 10:18 PM (8) Protein Annotation: Protein annotation might take significantly long time if outdated or on-disk FASTA files are used.

01/17/2021 10:18 PM (8) Protein Annotation: Protein database HUMAN.fasta needs update to create annotation groups. This might take a few minutes.

01/17/2021 10:18 PM (8) Protein Annotation: Start annotation of HUMAN.fasta

01/17/2021 10:19 PM (8) Protein Annotation: Annotations for 10000 of 89775 proteins added to the database

01/17/2021 10:20 PM (8) Protein Annotation: Annotations for 20000 of 89775 proteins added to the database

01/17/2021 10:20 PM (8) Protein Annotation: Annotations for 30000 of 89775 proteins added to the database

01/17/2021 10:21 PM (8) Protein Annotation: Annotations for 40000 of 89775 proteins added to the database

01/17/2021 10:22 PM (8) Protein Annotation: Annotations for 50000 of 89775 proteins added to the database

01/17/2021 10:24 PM (8) Protein Annotation: Annotations for 60000 of 89775 proteins added to the database

01/17/2021 10:25 PM (8) Protein Annotation: Annotations for 70000 of 89775 proteins added to the database

01/17/2021 10:26 PM (8) Protein Annotation: Annotations for 80000 of 89775 proteins added to the database

01/17/2021 10:27 PM (8) Protein Annotation: Annotations for 89775 of 89775 proteins added to the database

01/17/2021 10:27 PM (8) Protein Annotation: Save annotations of HUMAN.fasta in result file...

01/17/2021 10:27 PM (8) Protein Annotation: Start saving annotation descriptions in result file

01/17/2021 10:27 PM (8) Protein Annotation: Retrieving annotations from the Proteome Discoverer protein knowledge-base.

01/17/2021 10:29 PM (8) Protein Annotation: No chromosome information available, no

"Chromosome" column created.

01/17/2021 10:29 PM (8) Protein Annotation: Found annotations for 23989 of 23989 proteins.

01/17/2021 10:29 PM (8) Protein Annotation: Found 20241 annotation groups.

01/17/2021 10:29 PM (8) Protein Annotation: Found 2197 pathway groups.

01/17/2021 10:29 PM (8) Protein Annotation: Calculating associated data for annotation and pathway groups.

01/17/2021 10:29 PM (8) Protein Annotation: Preparing the annotation and pathway groups for result file

01/17/2021 10:29 PM (8) Protein Annotation: -- Total execution of Protein Annotation (8) took 11 min 19 s --

01/17/2021 10:29 PM (9) Protein Marker: Start marking proteins...

01/17/2021 10:31 PM (9) Protein Marker: Contaminants Detection took: 1 min 5 s

01/17/2021 10:31 PM (9) Protein Marker: Annotating species names took: 9.9 ms

01/17/2021 10:31 PM (9) Protein Marker: -- Total execution of Protein Marker (9) took 1 min 6 s --

01/17/2021 10:31 PM (13) Display Settings: Applying display filter and layout

01/17/2021 10:31 PM (13) Display Settings: -- Total execution of Display Settings (13) took 28.4 ms --

01/17/2021 10:31 PM (14) Data Distributions: Calculating found in files

01/17/2021 10:32 PM (14) Data Distributions: Calculating found for samples

01/17/2021 10:34 PM (14) Data Distributions: Calculating found for sample groups

01/17/2021 10:36 PM (14) Data Distributions: -- Total execution of Data Distributions (14) took 5 min 15 s --

01/17/2021 10:38 PM Job Execution: Finalizing file took 2 min 12 s.

01/17/2021 10:38 PM Job Execution: Finished C:₩Users₩프로테오믹스₩Desktop₩Data₩multi₩Multi_proteomics₩20201109_Multi_P1-1-(2).pdResult

01/17/2021 10:38 PM Job Execution: ----- Total Job execution took: 3 h 46 min. -----
