# Supplementary material for: Optimizing MS-Based Multi-Omics: Comparative Analysis of Protein, Metabolite, and Lipid Extraction Techniques
Source: Metabolites. 2024 Jan 3;14(1):34. doi: 10.3390/metabo14010034 (PMC10820684; doi:10.3390/metabo14010034)
Supplement: Supplementary file 1 [file metabolites-14-00034-s001.zip › Supplementary material.pdf]

Supplementary material

Optimizing MS -based multi-omics: comparative analysis of protein, metabolite, and lipid extraction techniques

Jeong-Hun Mok, ‡[a] Minjoong Joo,‡[b] Seonghyeon Cho,[b] Van-An Duong,[c] Haneul Song,[c] Jong-Moon Park*[b] and Hookeun Lee*[c]

[a.] *Department of Medical Device Management and Research, SAIHST, Sungkyunkwan University, 115, Irwon-ro, Gangnam-gu, Seoul (06355), Republic of Korea. Email: jeonghunmok@naver.com (J.-H.M.)*

[b.] *Basilbiotech, 157-20, Sinsong-ro, Yeonsu-gu, Incheon 22002, Republic of Korea; mjjoo@basilbiotech.com (M.J.); shcho@basilbiotech.com (S.C.); basil@basilbiotech.com (J.-M.P)*

[c.] *College of Pharmacy, Gachon University, 191, Hambangmoe-ro, Yeonsu-gu, Incheon 21936 , Republic of Korea; Email: anduong@gachon.ac.kr (V.-A.D.); 202240509@gachon.ac.kr (H.S.); hklee@gachon.ac.kr (H.L.)*

‡These authors contributed equally.

Table of Content

Figure S1. Chromatogram of proteomics data of each method.

Figure S2. List of the number of metabolite features, compounds identified as level of confidence 2,3 for each extraction method

Figure S3. Histogram for RSD of each method(metabolite).

Figure S4. Chromatogram of metabolomics data of each method.

Figure S5. ID grade graph for each extraction method of lipids identified by lipid search. (ID Quality filter- Grade A：Lipid of which fatty acid chains and class were identified completely./ Grade B：Lipid of which class and some fatty acid chains were identified./Grade C：Lipid of which class or fatty acid was identified./Grade D：other)

Figure S6. Chromatogram of lipidomics data of each method.

Figure S7. Number of missed cleavages in identified peptides. (A) FASP_80methanol, (B) FASP_Chloroform/methanol, (C) FASP_MTBE, (D) S-trap_80methanol, (E) S-trap_Chloroform/methanol , (F) S-trap_MTBE

Figure S8. Correlation scatter plot in proteomics data

Figure S9. Histogram of pI(isoelectric points)

Figure S10. Histograms and cumulative distributions of molecular weight(MW)

Table S2. Fold change and p-value of Q71DI3, Q9BW91, Q7Z417

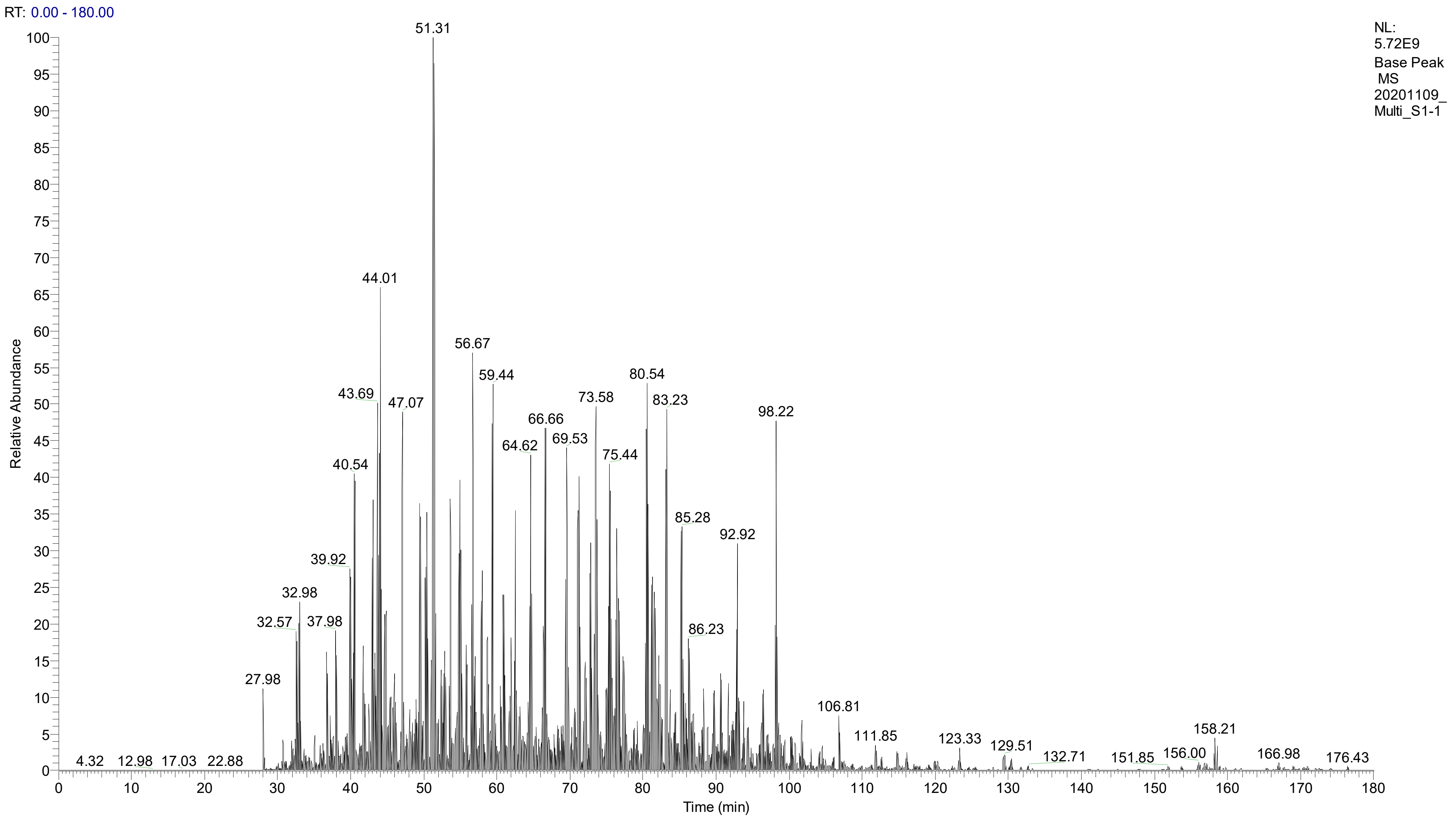

(A)80%methanol + S-trap-2

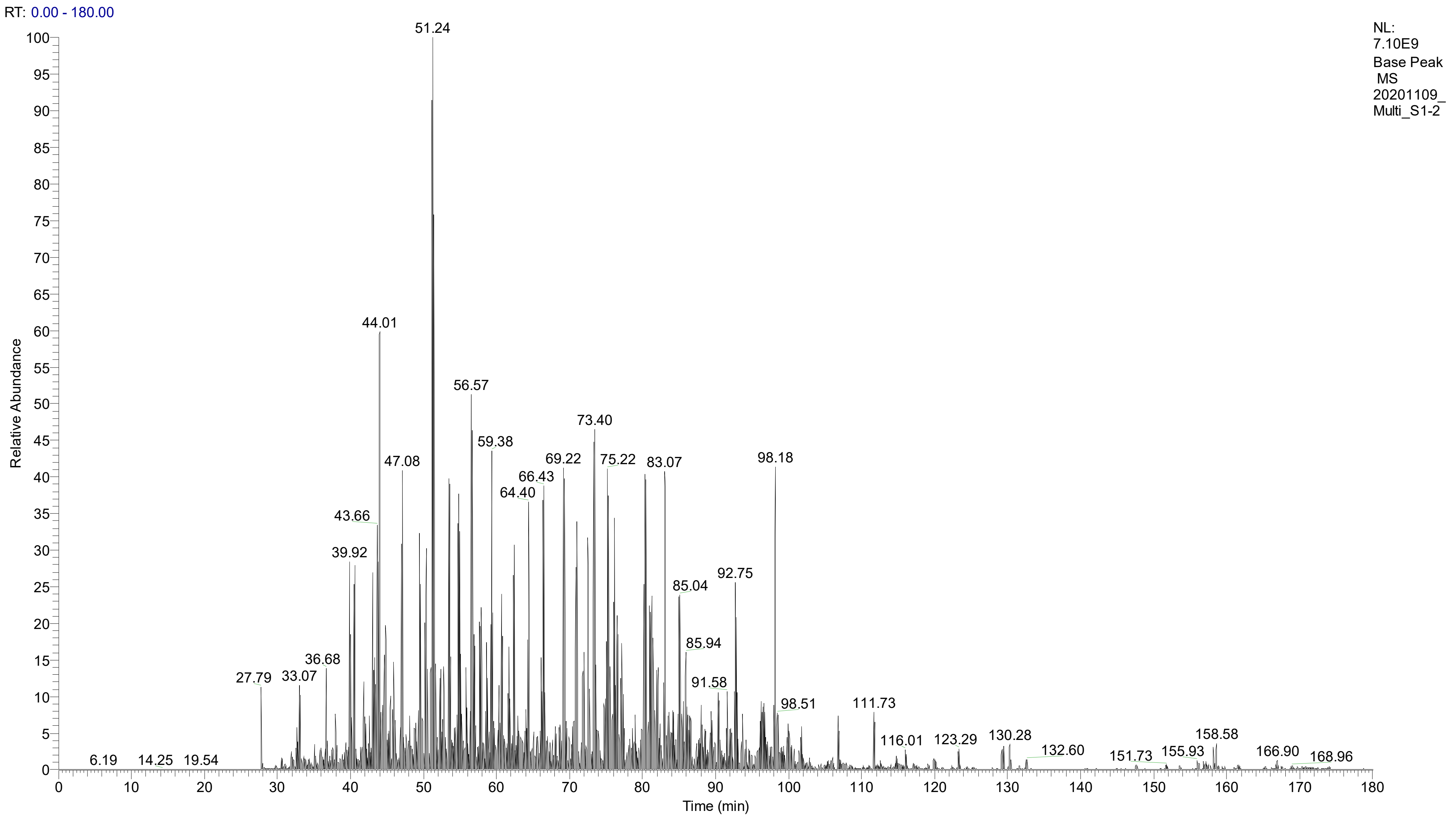

(A)80%methanol + S-trap-3

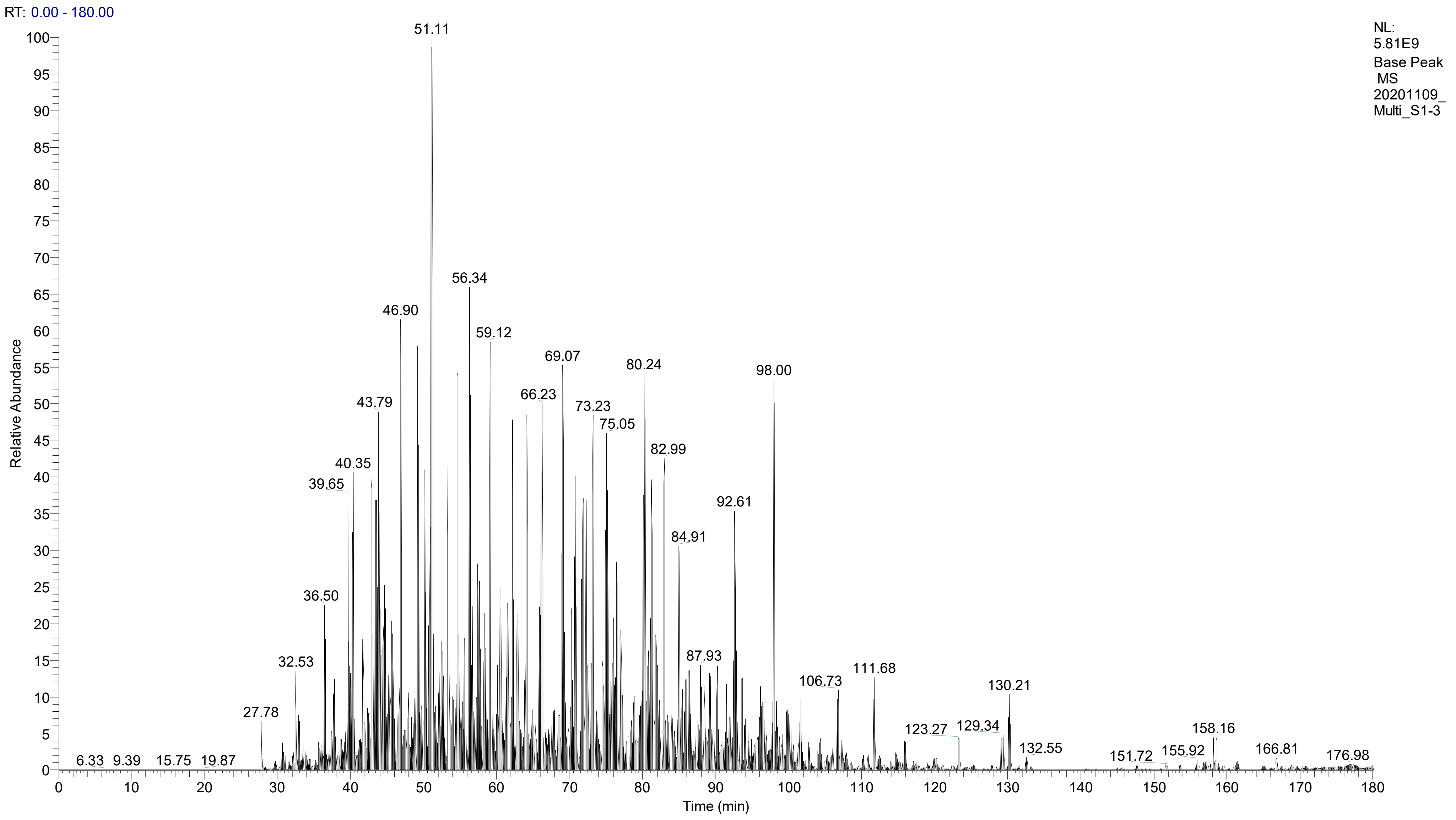

(B)Chloroform/methanol + S-trap-1

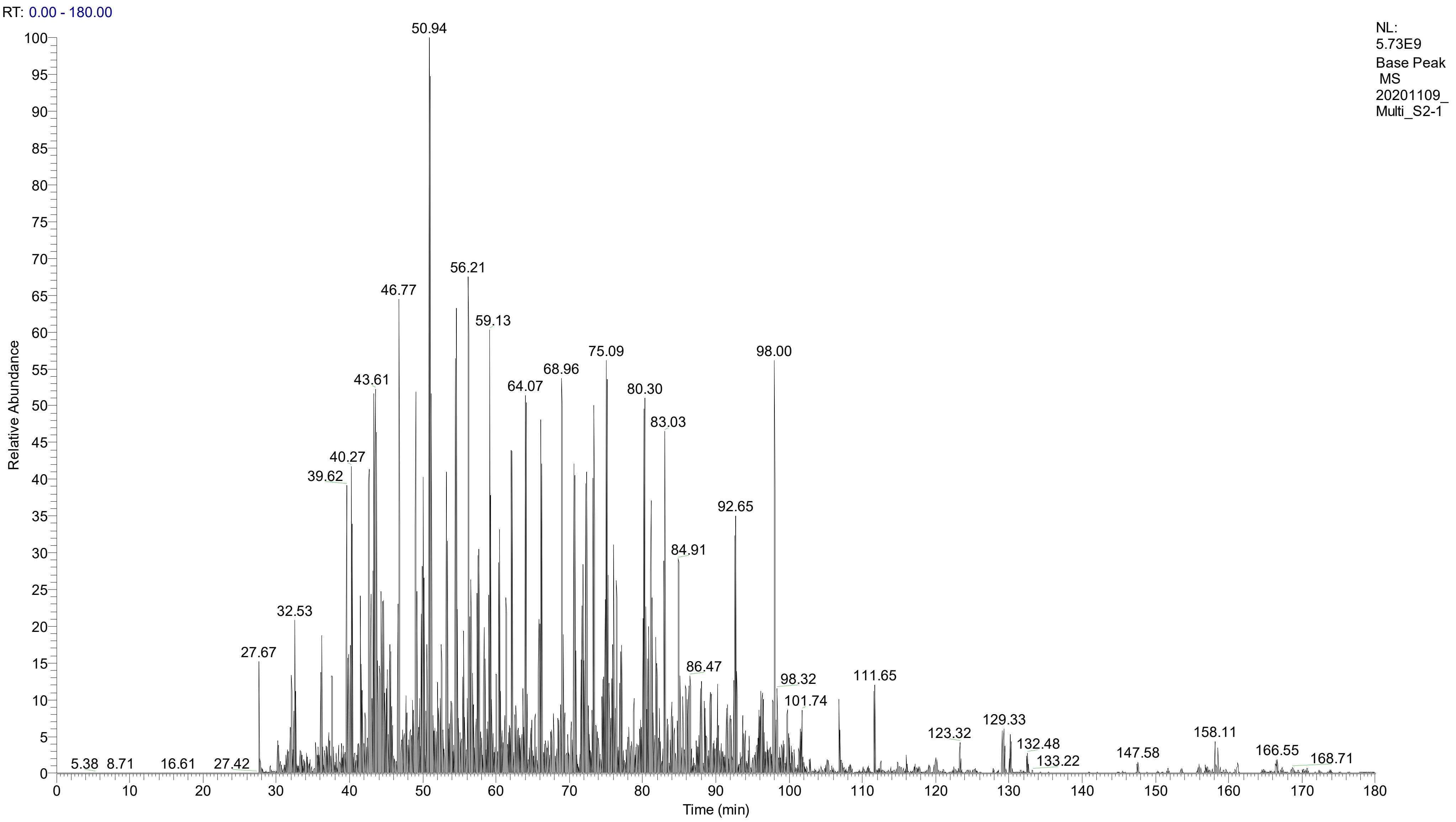

(B)Chloroform/methanol + S-trap-2

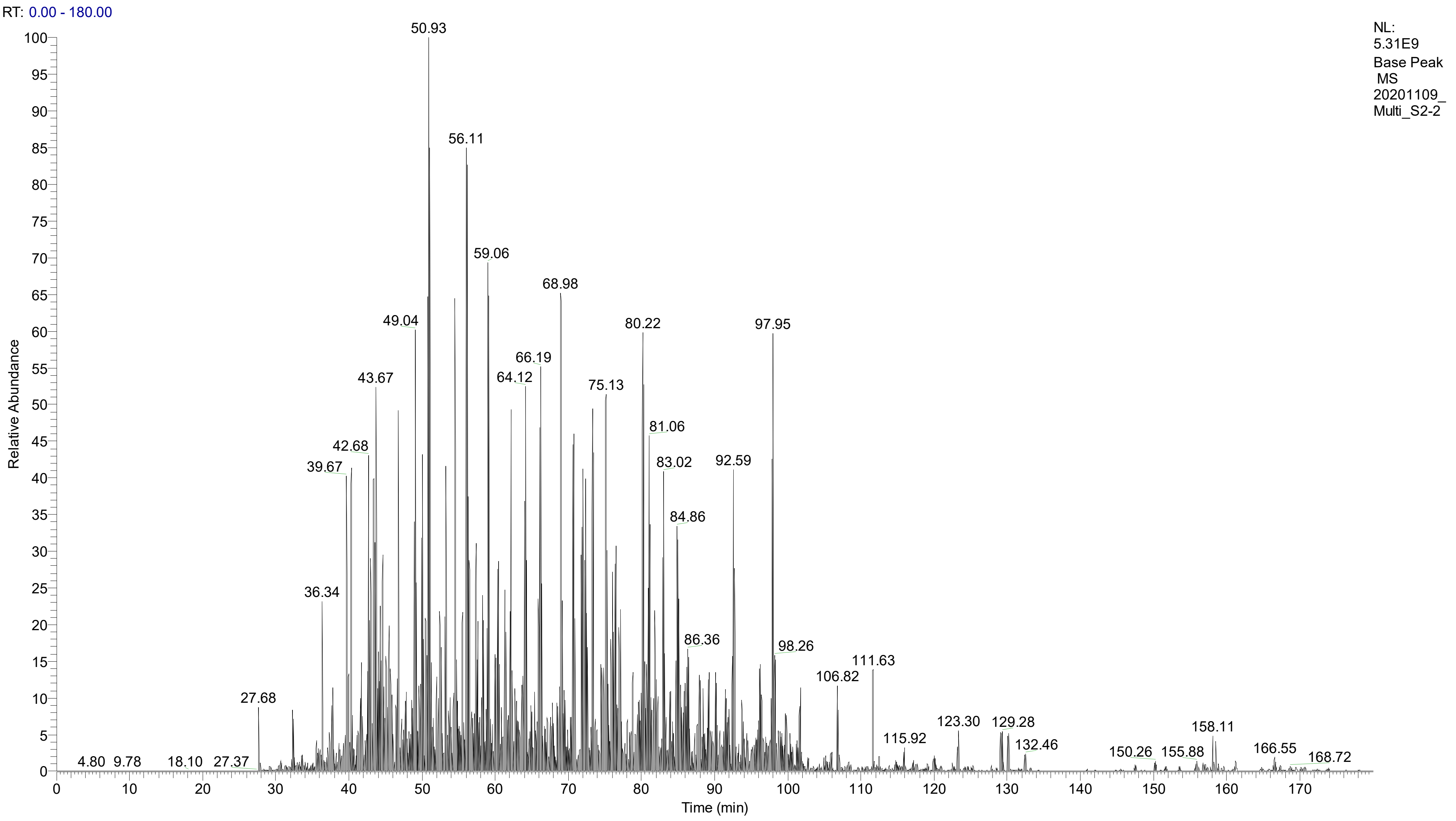

(B)Chloroform/methanol + S-trap-3

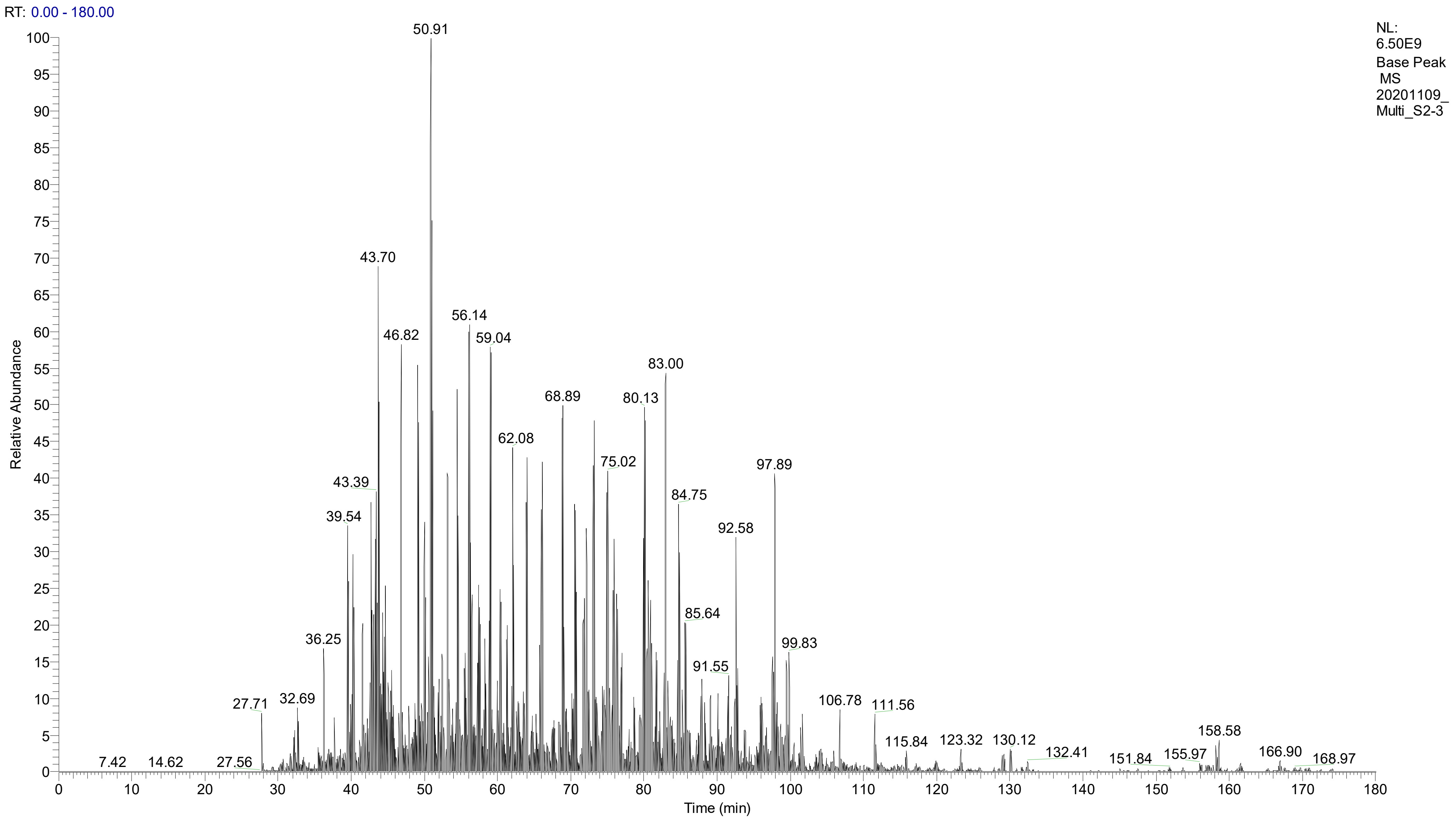

(C)MTBE + S-trap-1

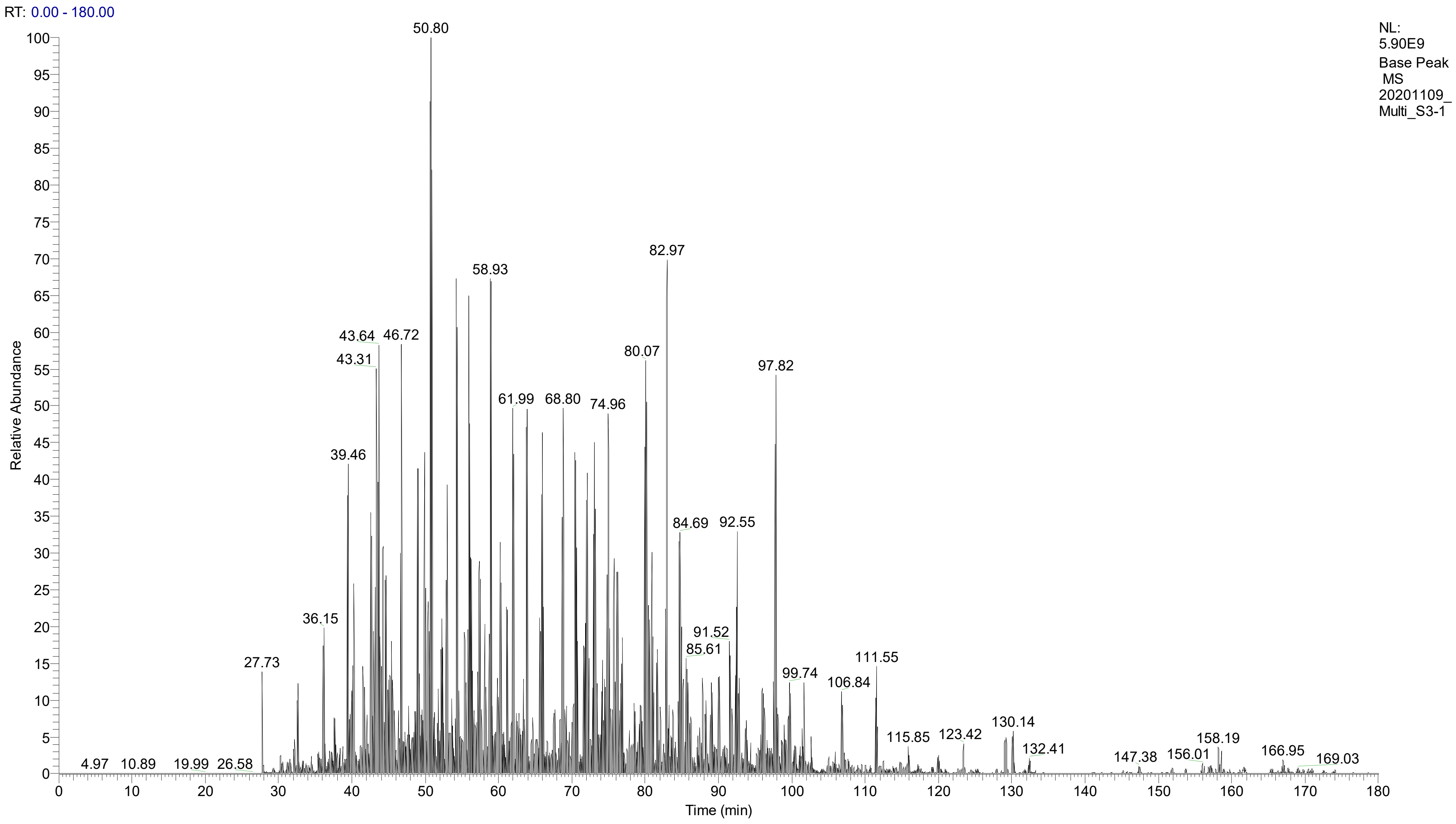

(C)MTBE + S-trap-2

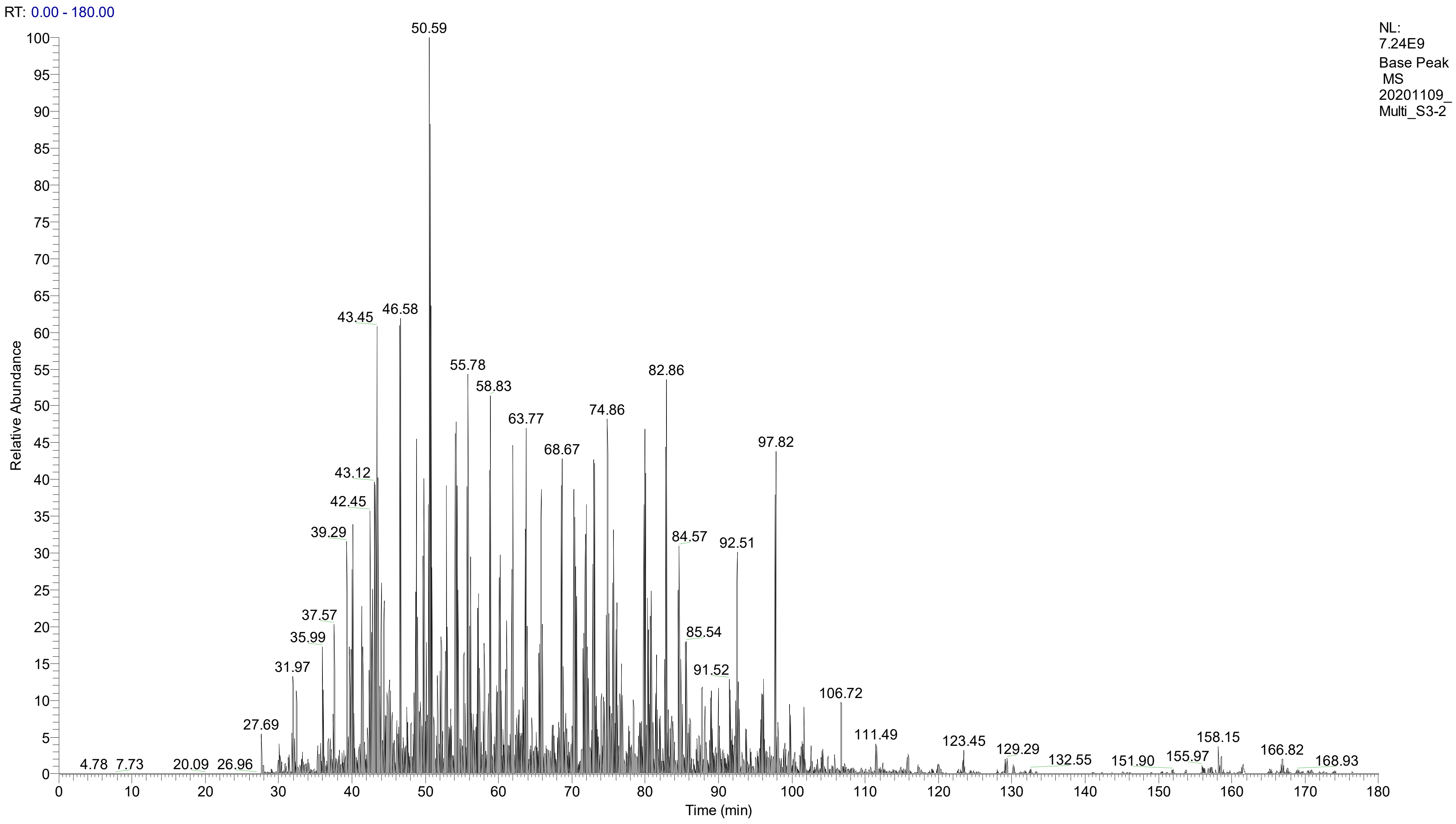

(C)MTBE + S-trap-3

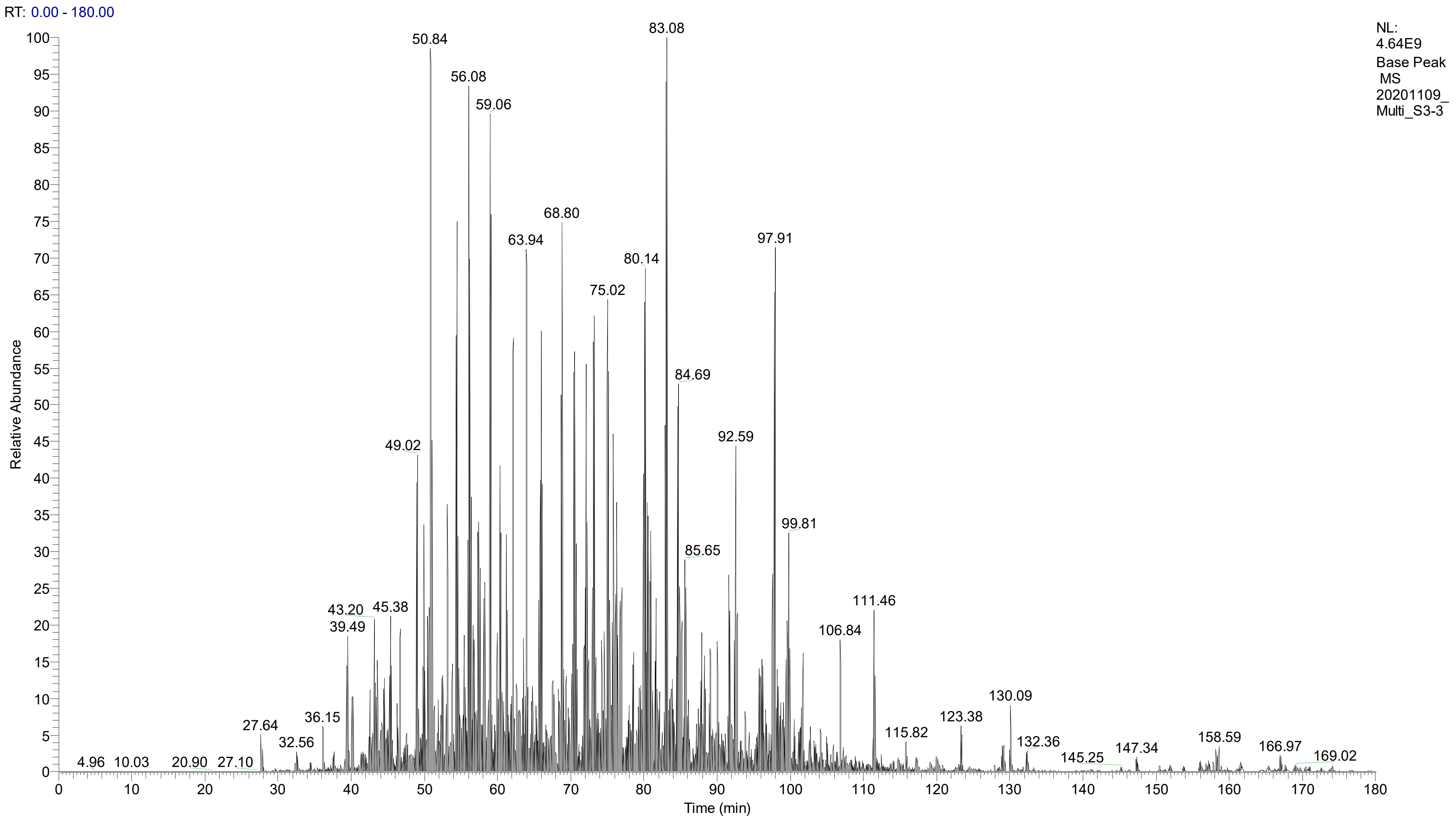

(D)80%methanol + FASP-1

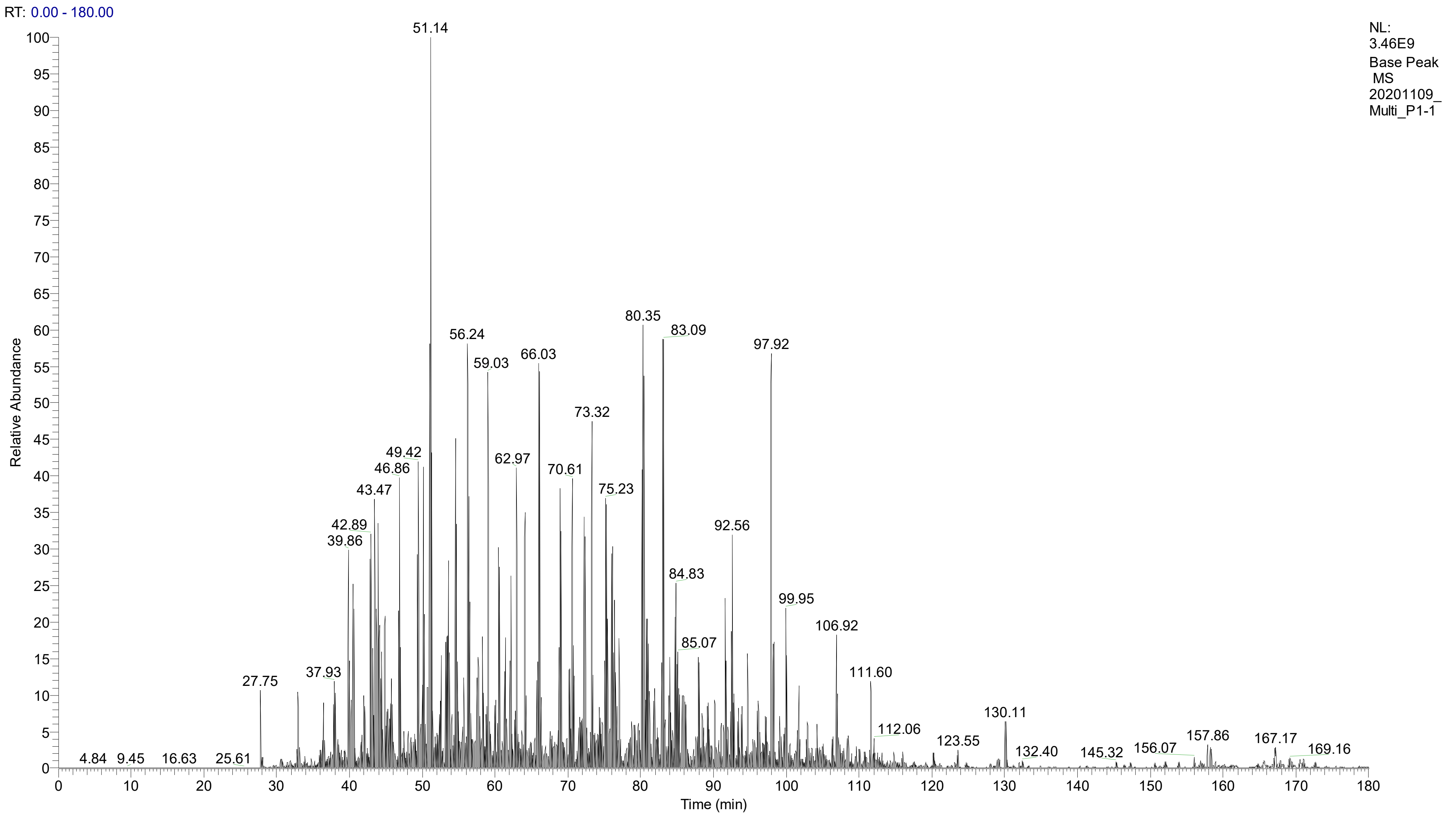

(D)80%methanol + FASP-2

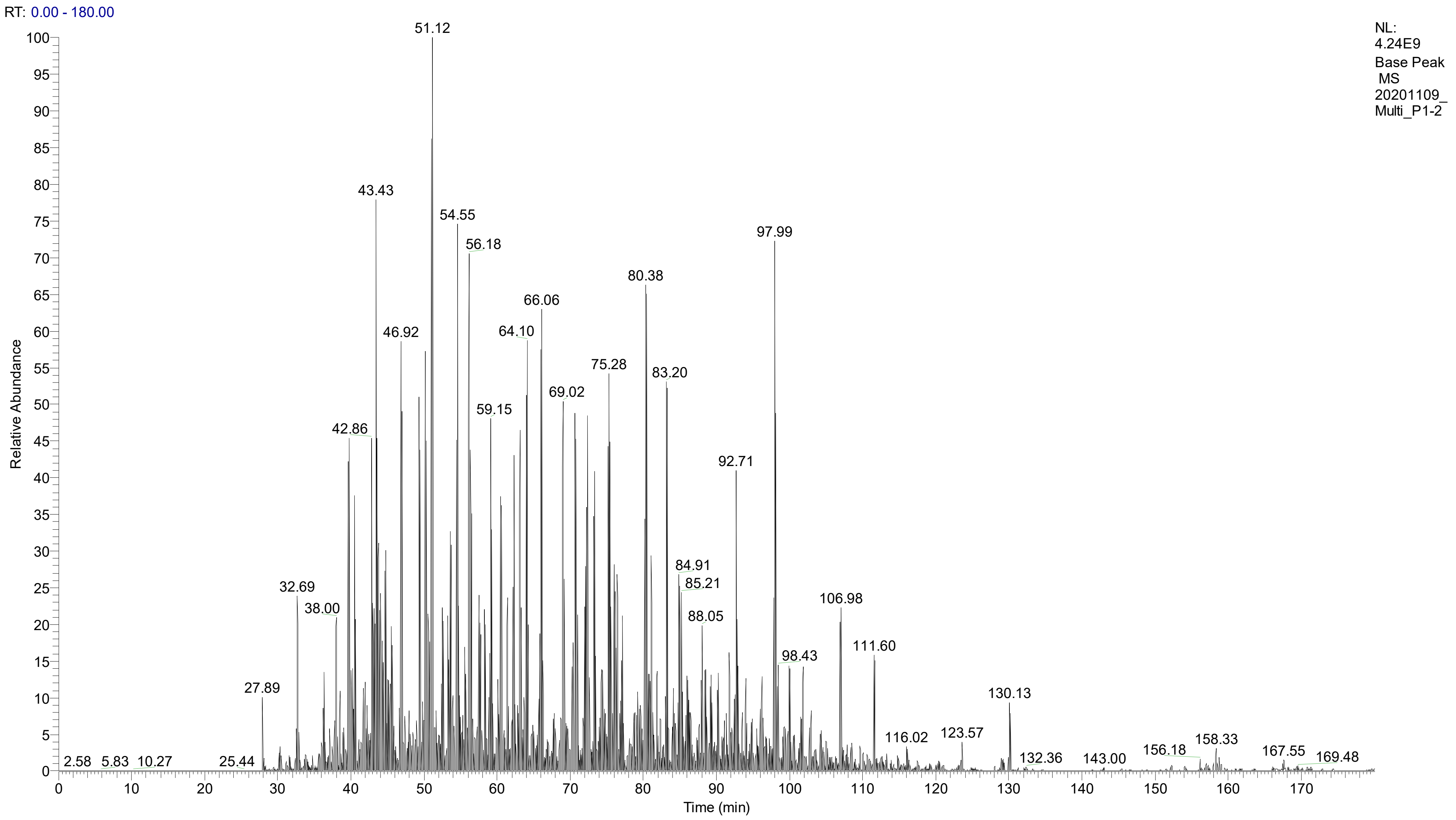

(D)80%methanol + FASP-3

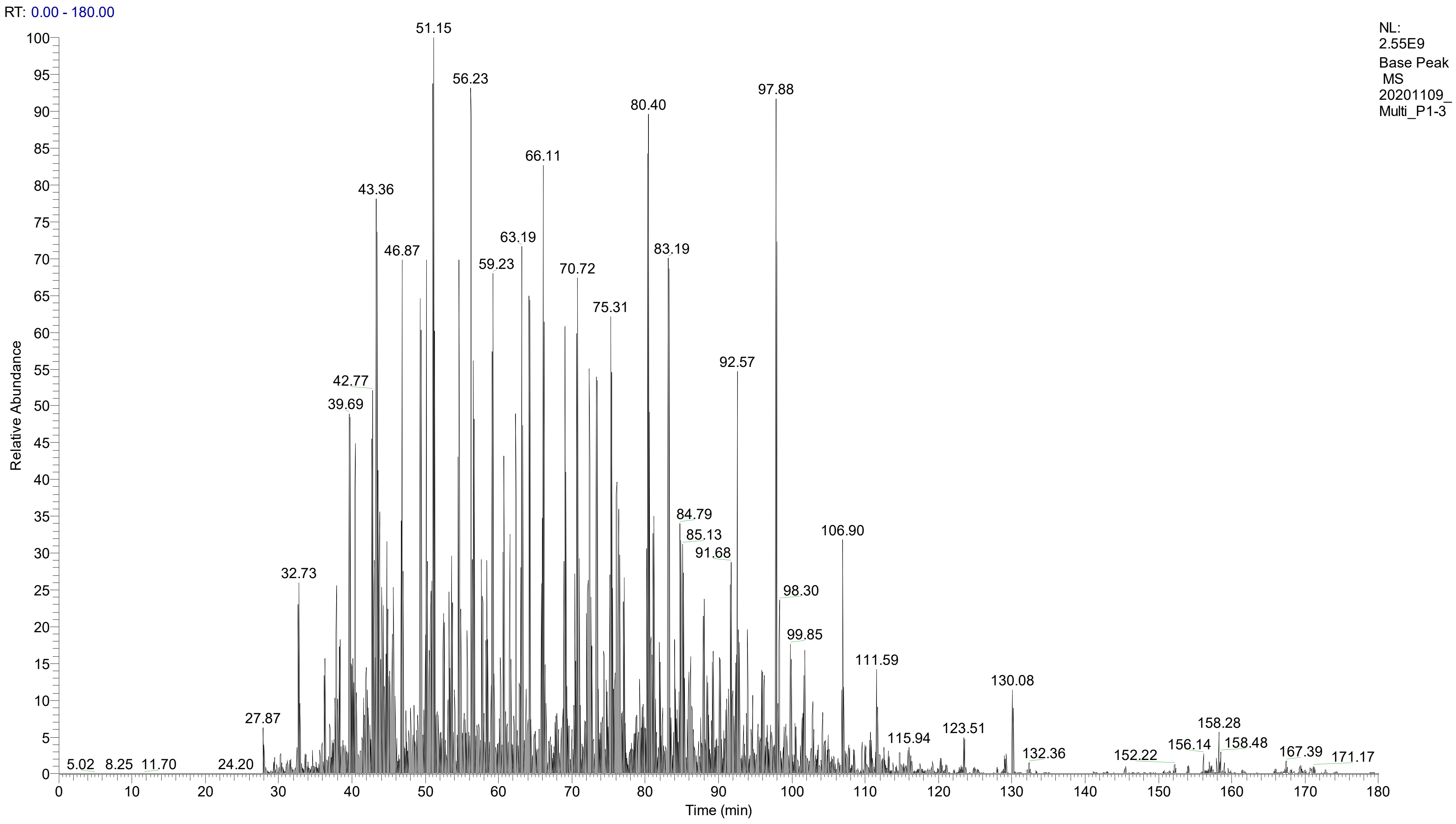

(E)Chloroform/methanol + FASP-1

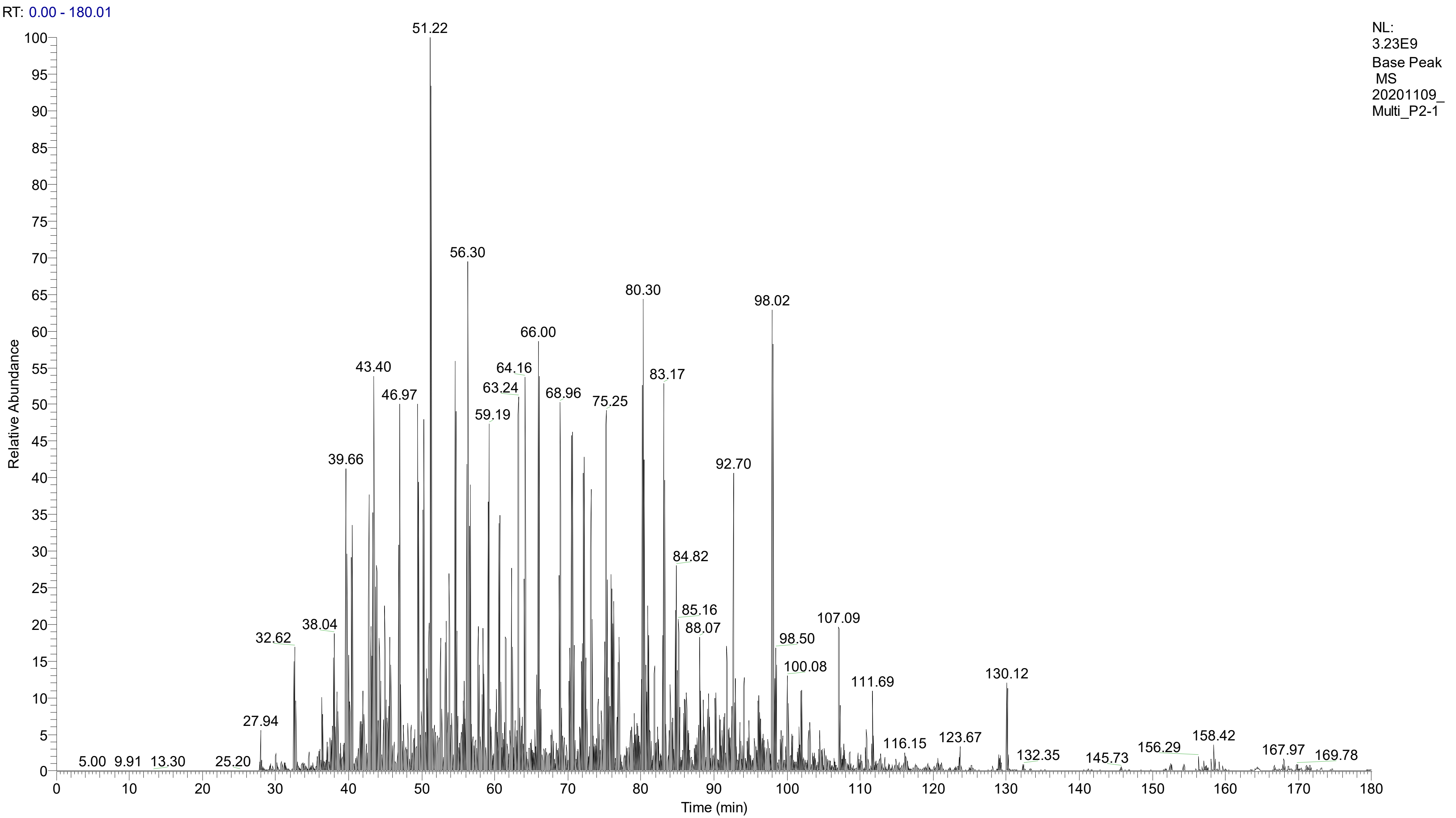

(E)Chloroform/methanol + FASP-2

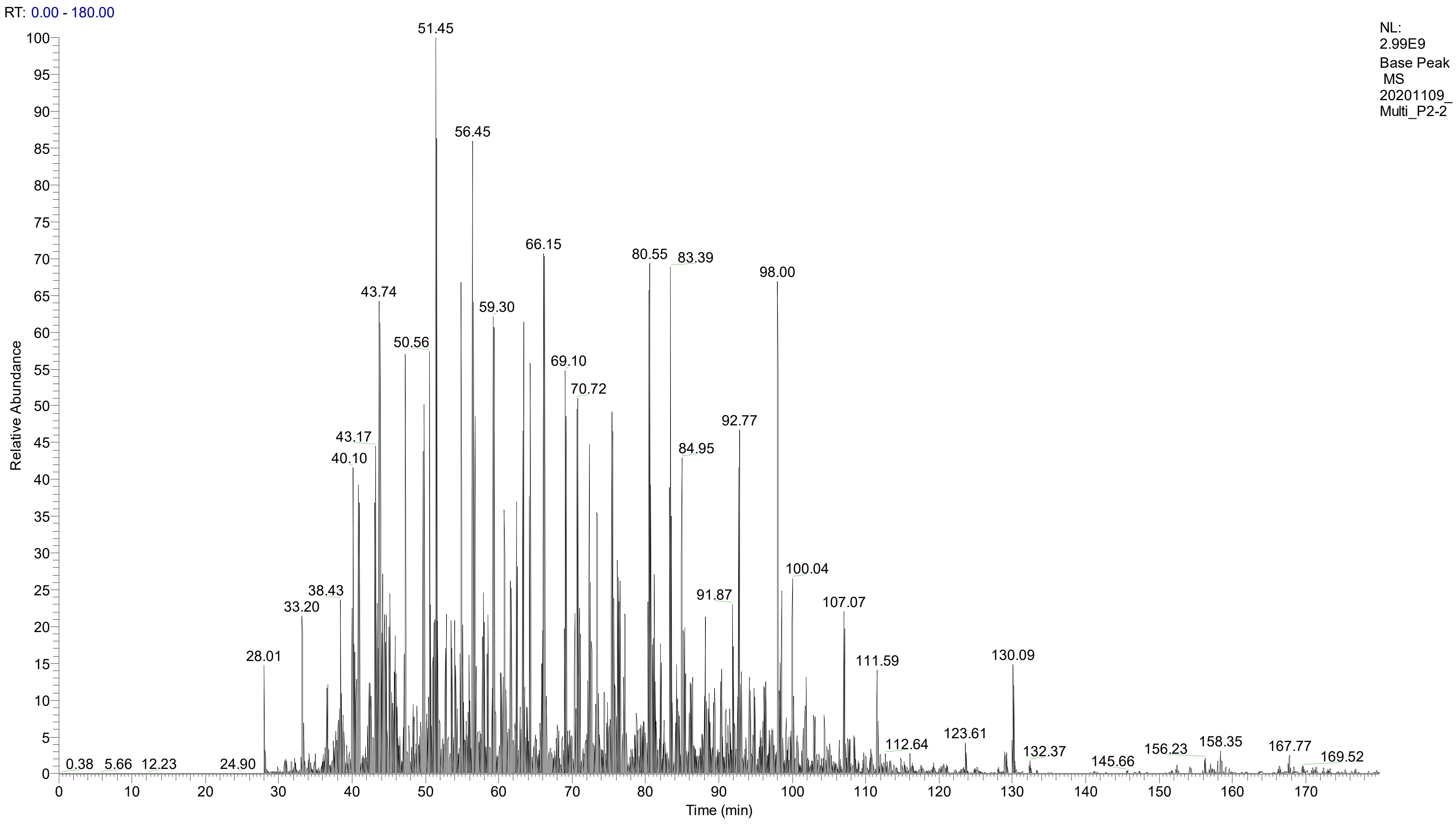

(E)Chloroform/methanol + FASP-3

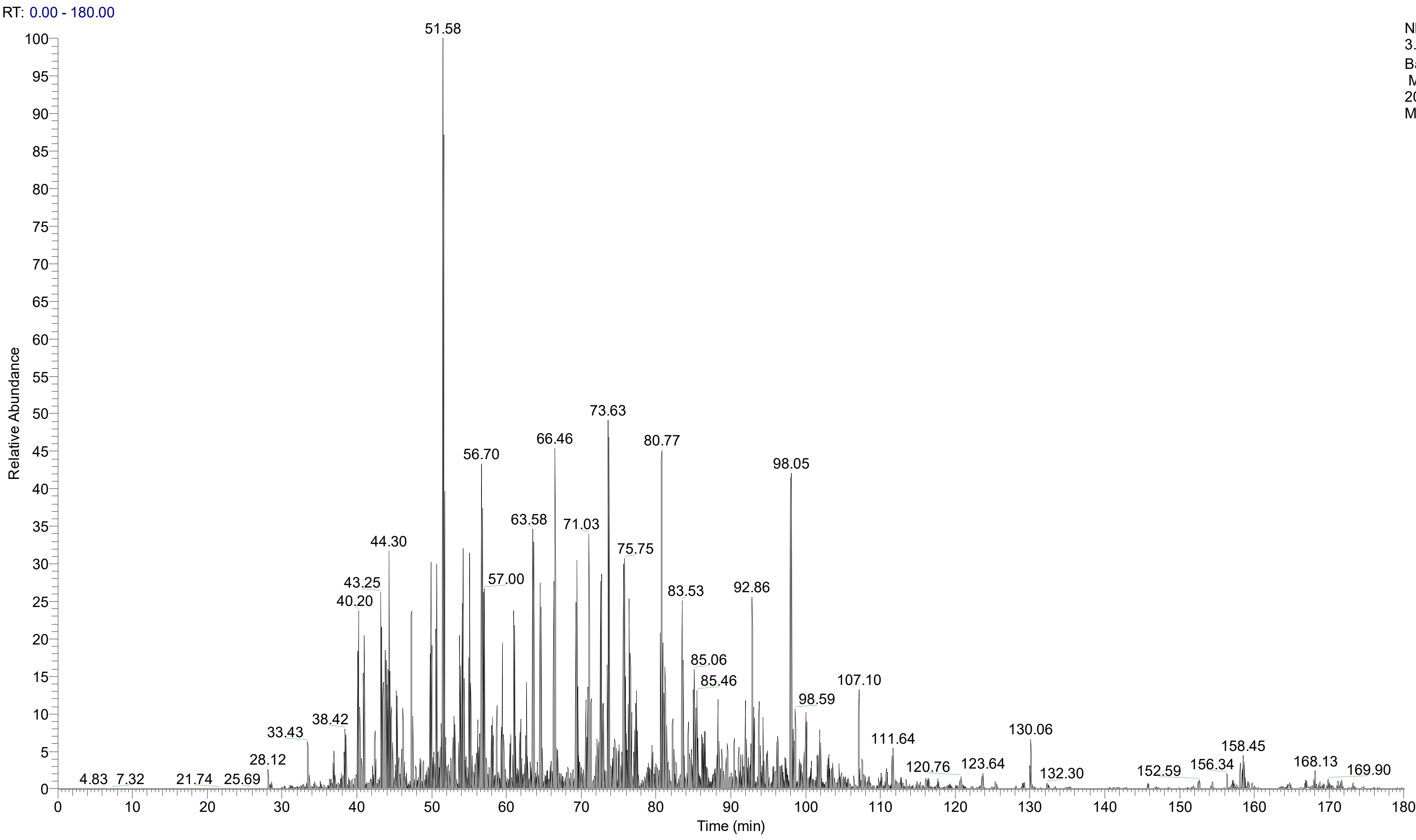
RT: 0.00 - 180.00
NL:
3.23E9
Base Peak
MS
20201109_
Multi_P2-3
Relative Abundance
Time (min)
51.58
73.63
66.46
80.77
56.70
98.05
63.58
71.03
44.30
75.75
43.25
57.00
40.20
83.53
92.86
85.06
85.46
107.10
98.59
38.42
33.43
111.64
130.06
158.45
28.12
120.76
123.64
168.13
169.90
156.34
152.59
132.30
4.83
7.32
21.74
25.69

(F)MTBE + FASP-1

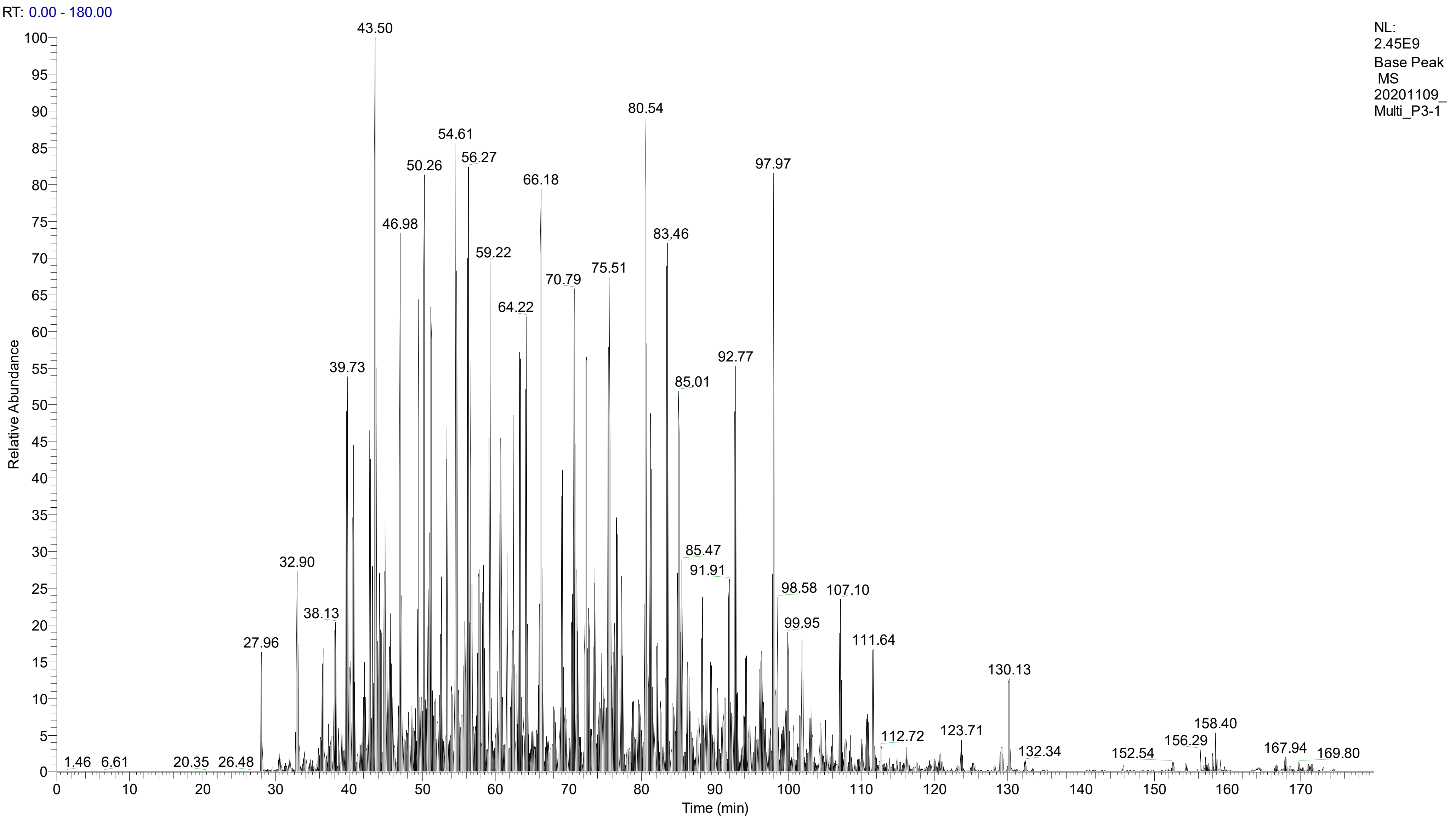
RT: 0.00 - 180.00
NL:
2.45E9
Base Peak
MS
20201109_
Multi_P3-1
Relative Abundance
Time (min)
43.50
80.54
54.61
56.27
50.26
97.97
66.18
46.98
83.46
59.22
75.51
70.79
64.22
39.73
92.77
85.01
32.90
85.47
91.91
98.58
107.10
38.13
99.95
27.96
111.64
130.13
158.40
112.72
123.71
156.29
167.94
132.34
152.54
169.80
1.46
6.61
20.35
26.48

(F)MTBE + FASP-2

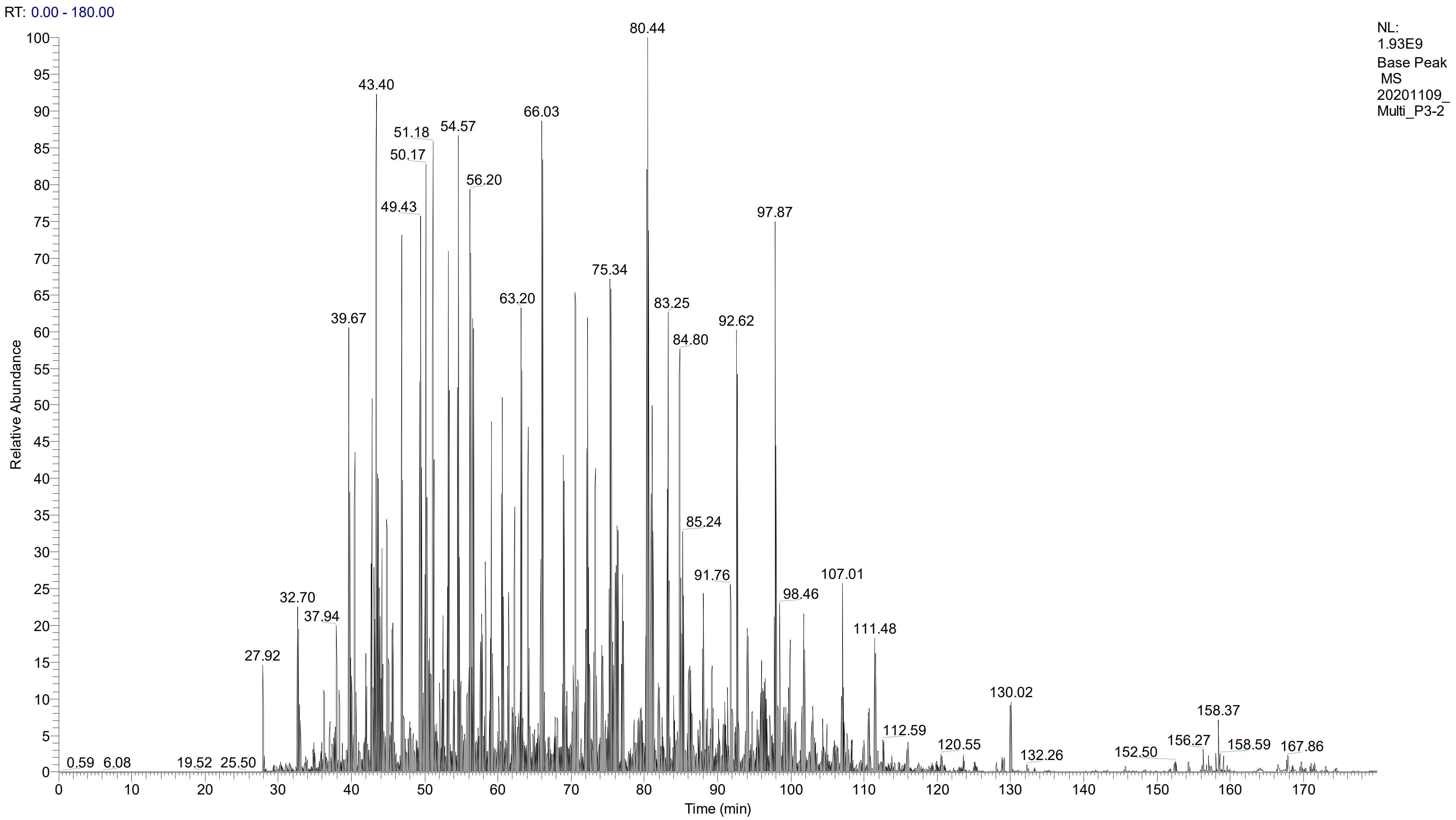
RT: 0.00 - 180.00
NL:
1.93E9
Base Peak
MS
20201109_
Multi_P3-2
Relative Abundance
Time (min)
80.44
43.40
66.03
54.57
51.18
50.17
56.20
49.43
97.87
75.34
63.20
83.25
39.67
92.62
84.80
85.24
91.76
107.01
98.46
32.70
37.94
111.48
27.92
130.02
158.37
112.59
156.27
120.55
158.59
167.86
152.50
132.26
0.59
6.08
19.52
25.50

(F)MTBE + FASP-3

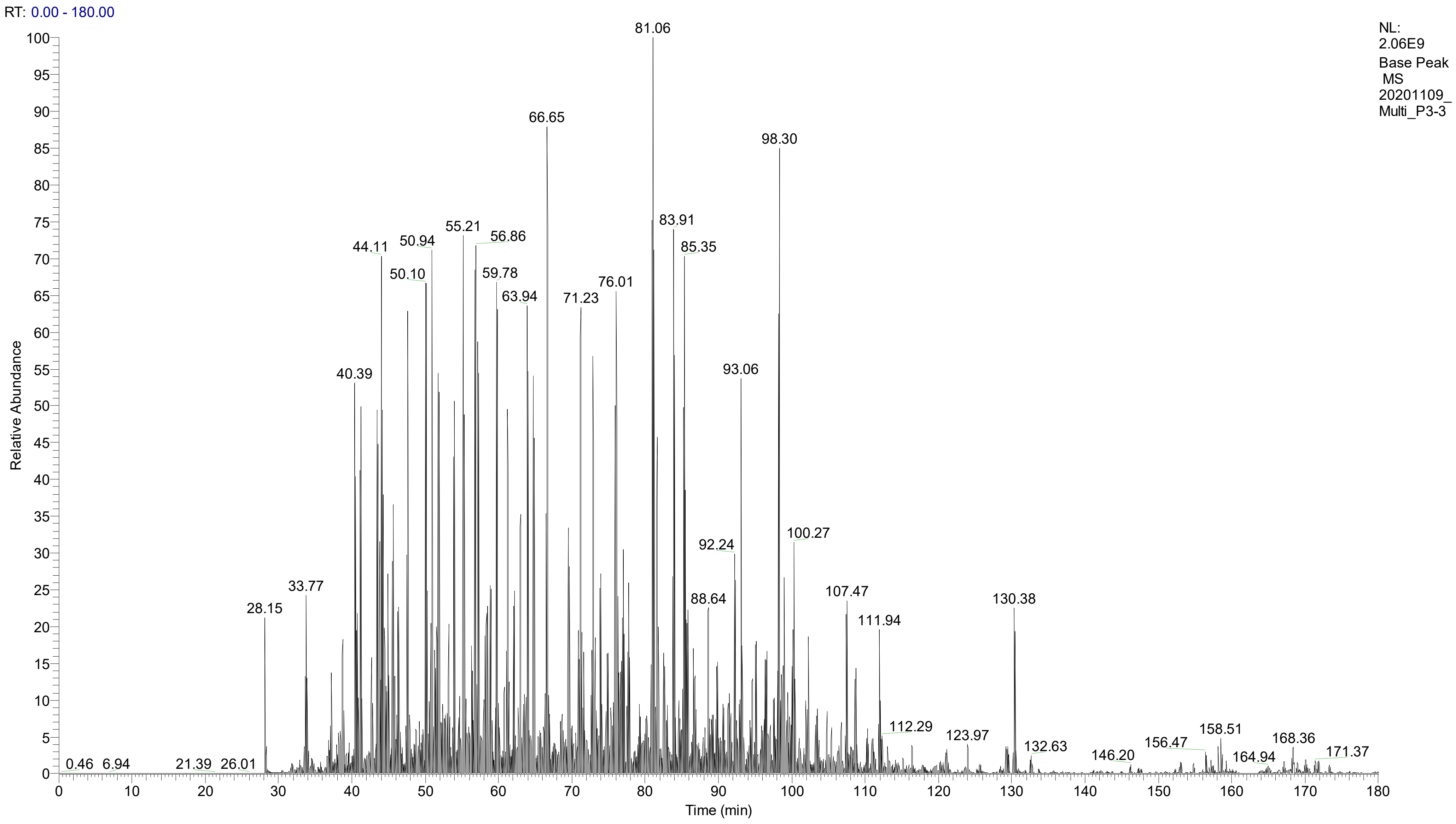

Figure S1 Chromatogram of proteomics data of each method.

	Label	Feature	ID(compound)<br>Level of Confidence : 3	ID(compound)<br>Level of Confidence : 2
80%methanol	1-1	3179	568	61
	1-2	3108	543	63
	1-3	3158	545	86
Chloroform/methanol	2-1	2306	434	64
	2-2	2126	418	69
	2-3	2360	458	61
MTBE	3-1	2680	496	74
	3-2	2549	480	66
	3-3	2510	460	74

Figure S2 List of the number of metabolite features, compounds identified as level of confidence 2,3 for each extraction method

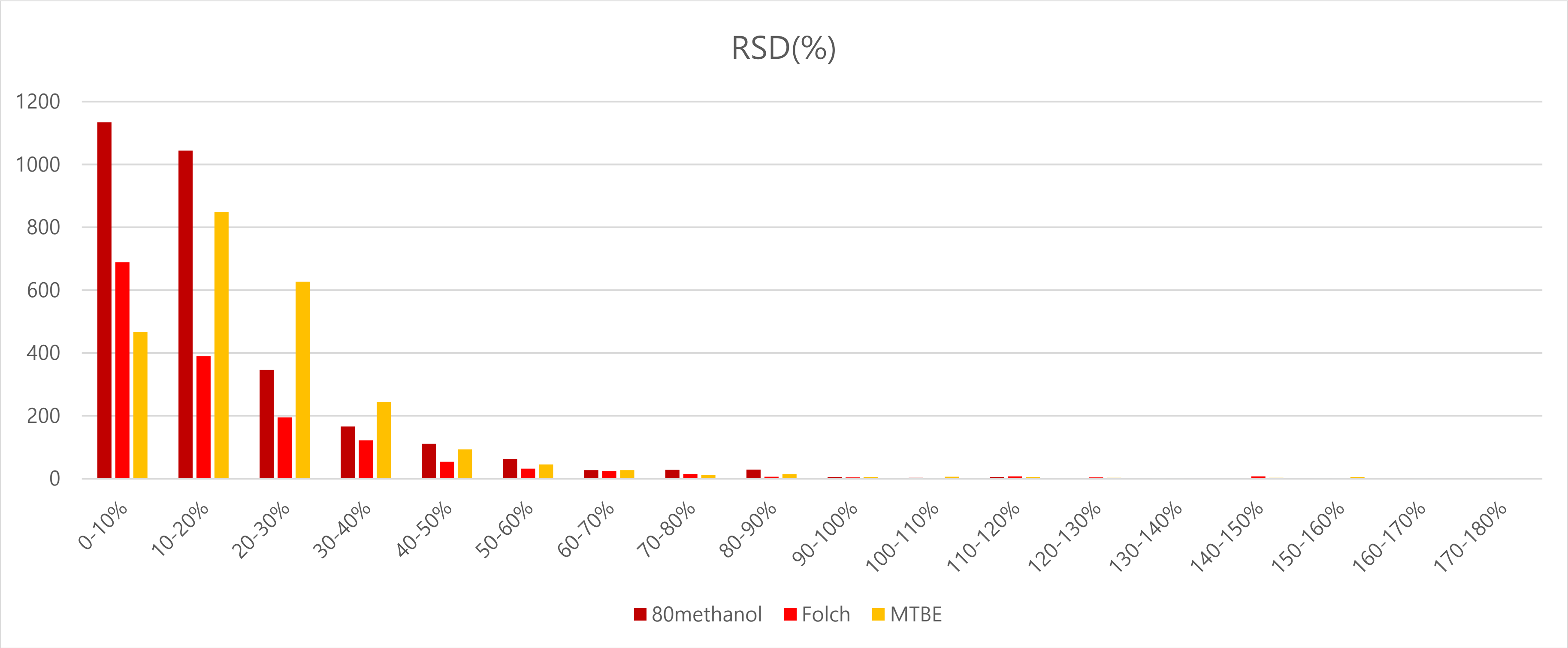

Figure S3 Histogram for RSD of each method(metabolite).

(A)80%methanol + S-trap-1

(A)80%methanol-1

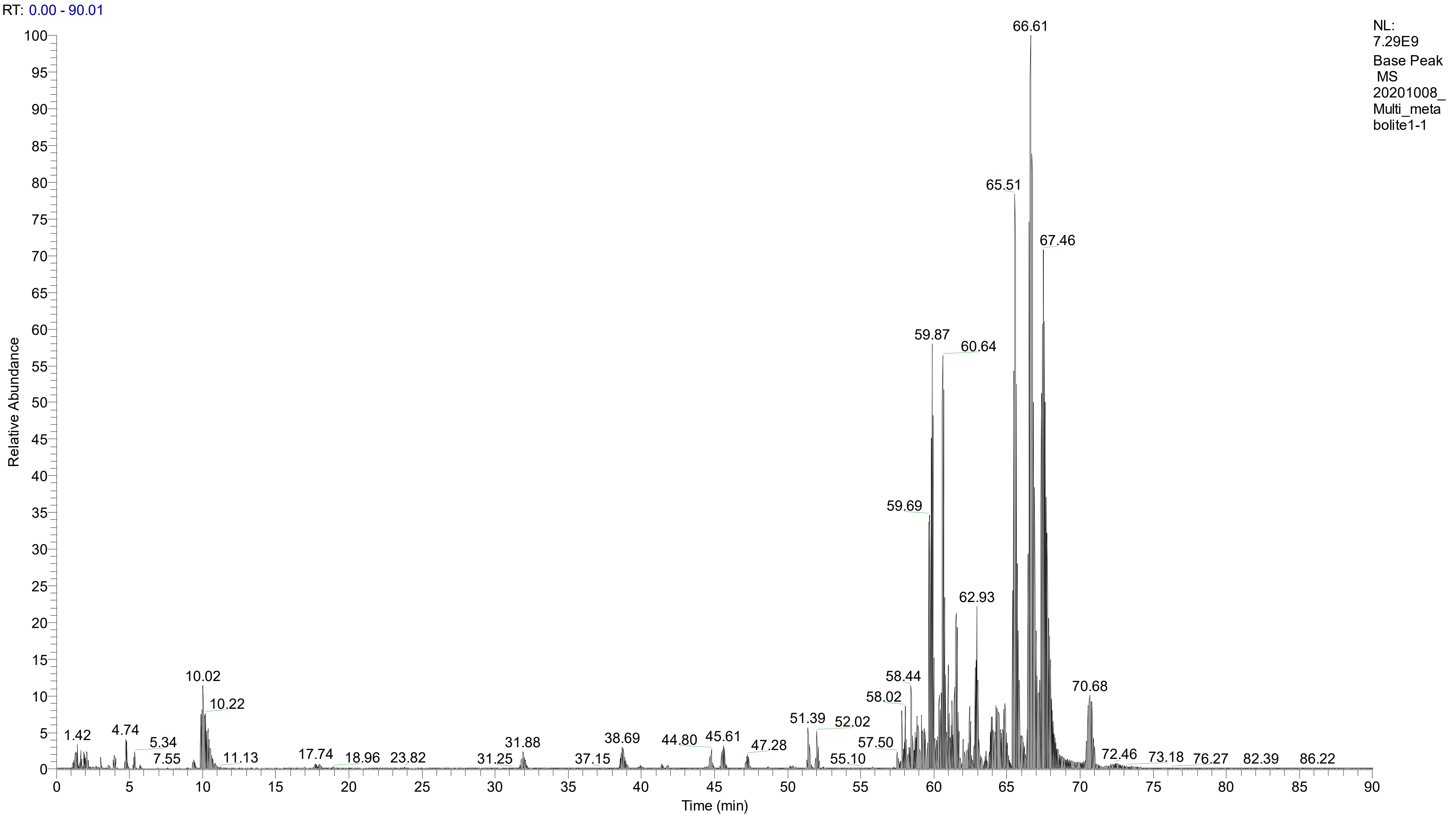

(A)80%methanol-2

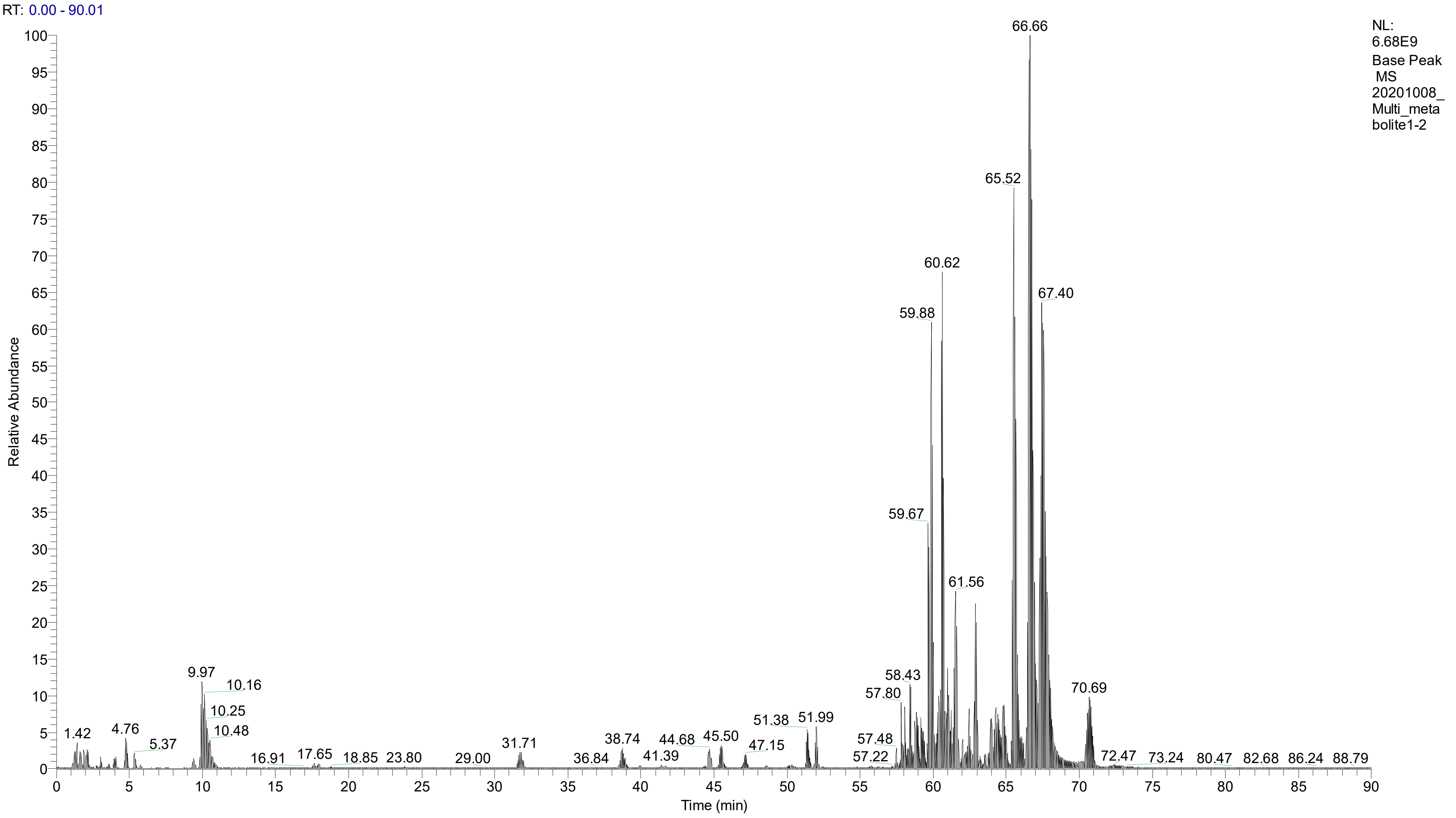

(A)80%methanol-3

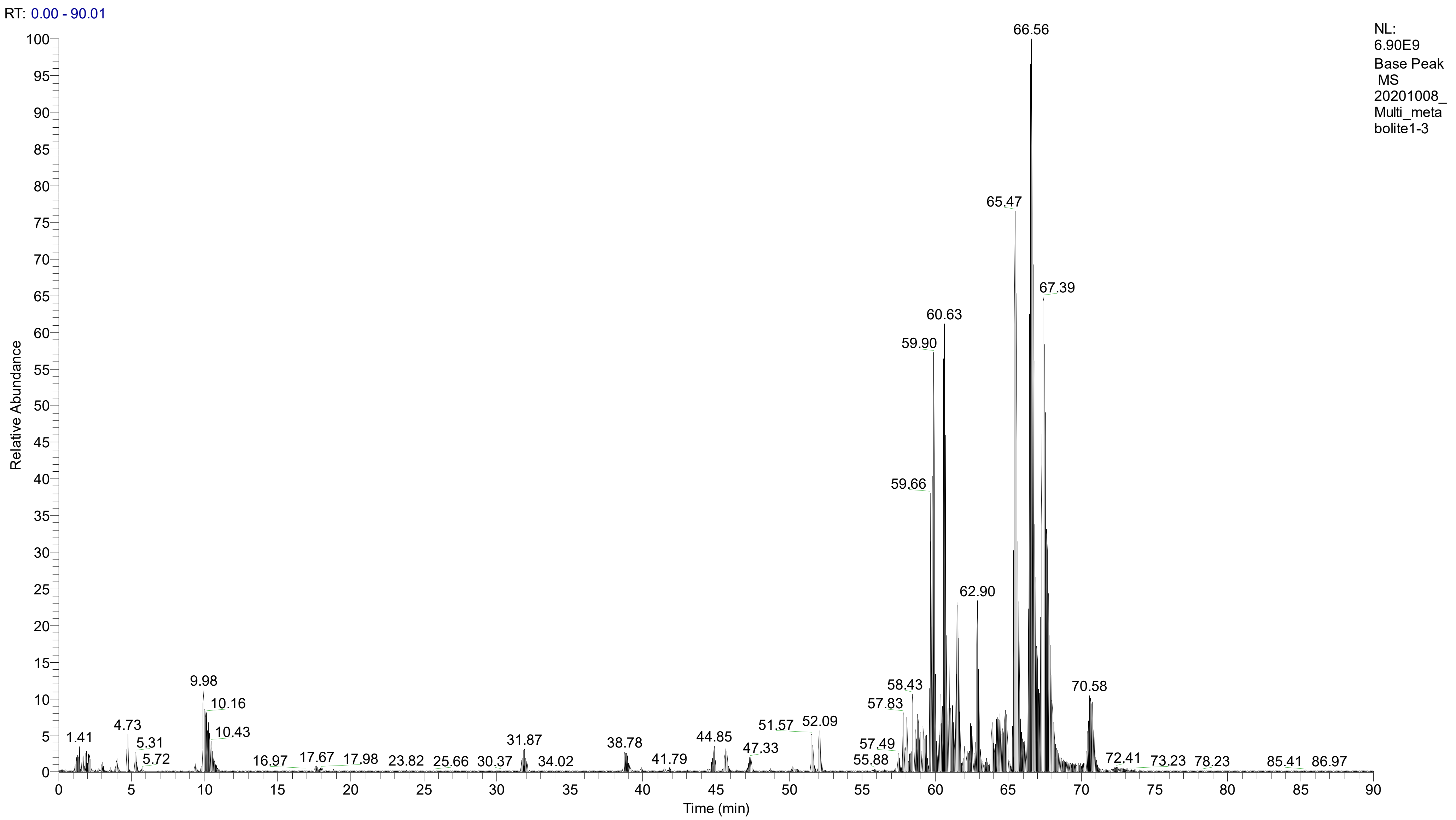

(B)Chloroform/methanol-1

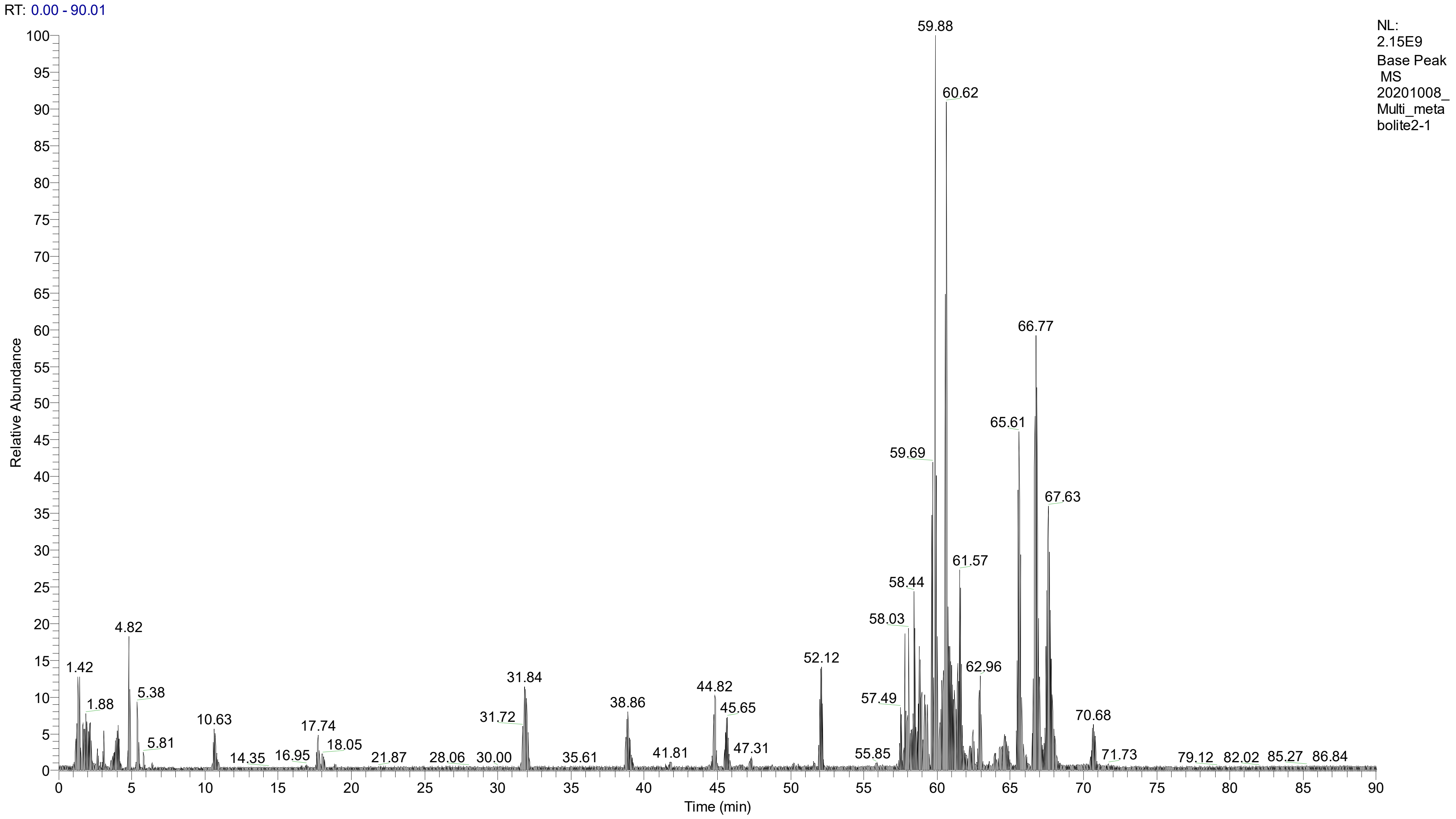

(B)Chloroform/methanol-2

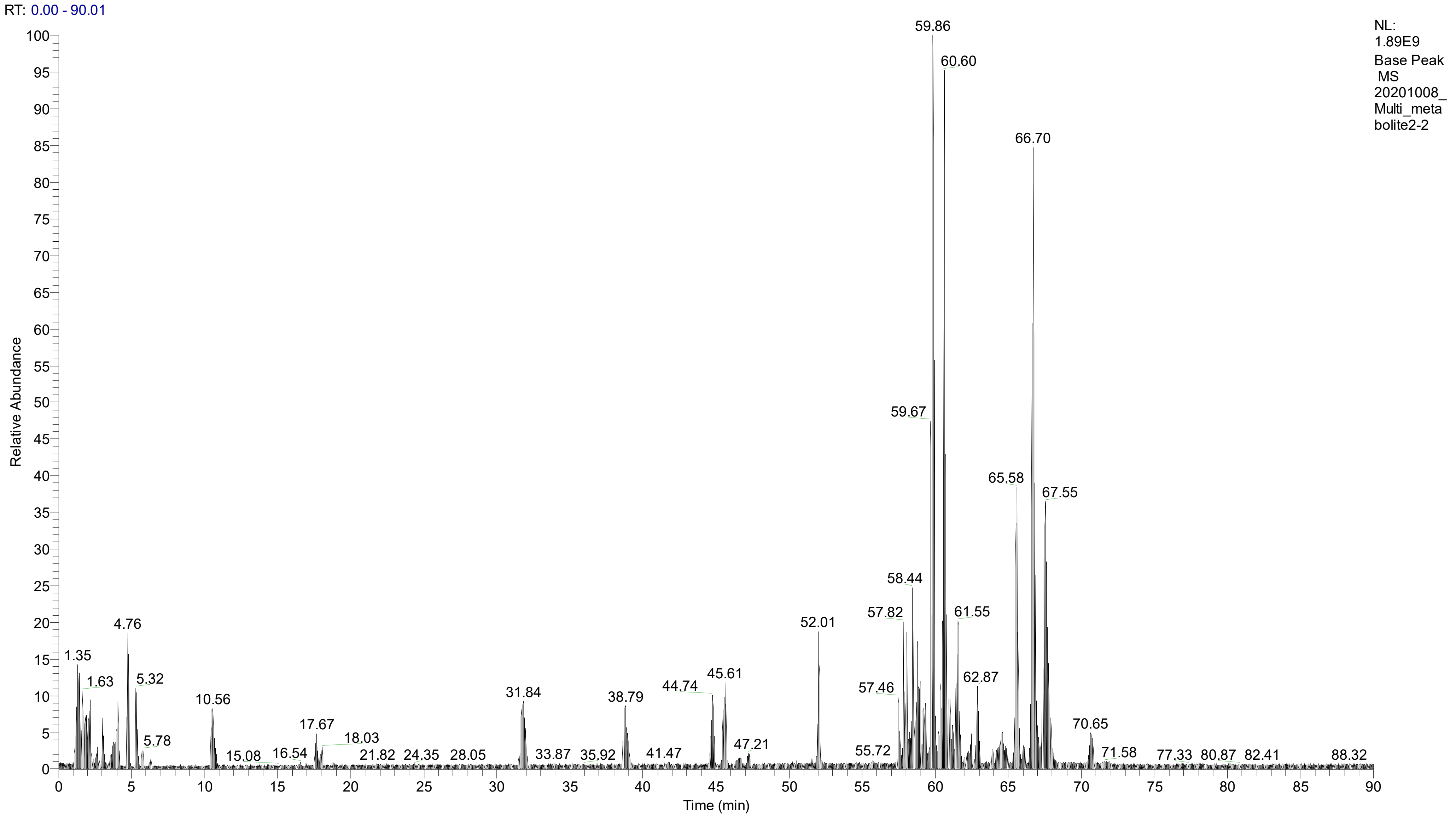

(B)Chloroform/methanol-3

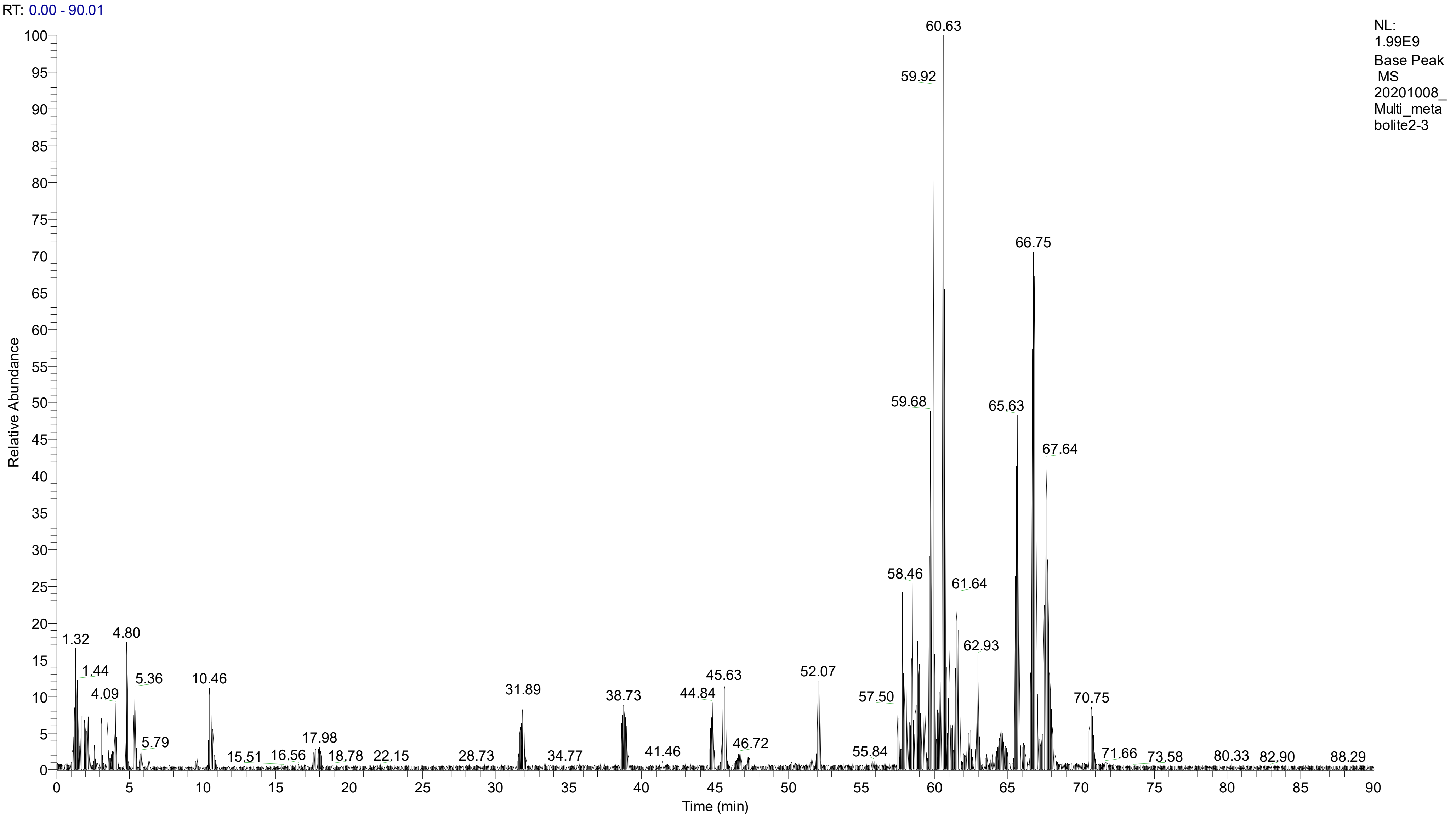

(C)MTBE-1

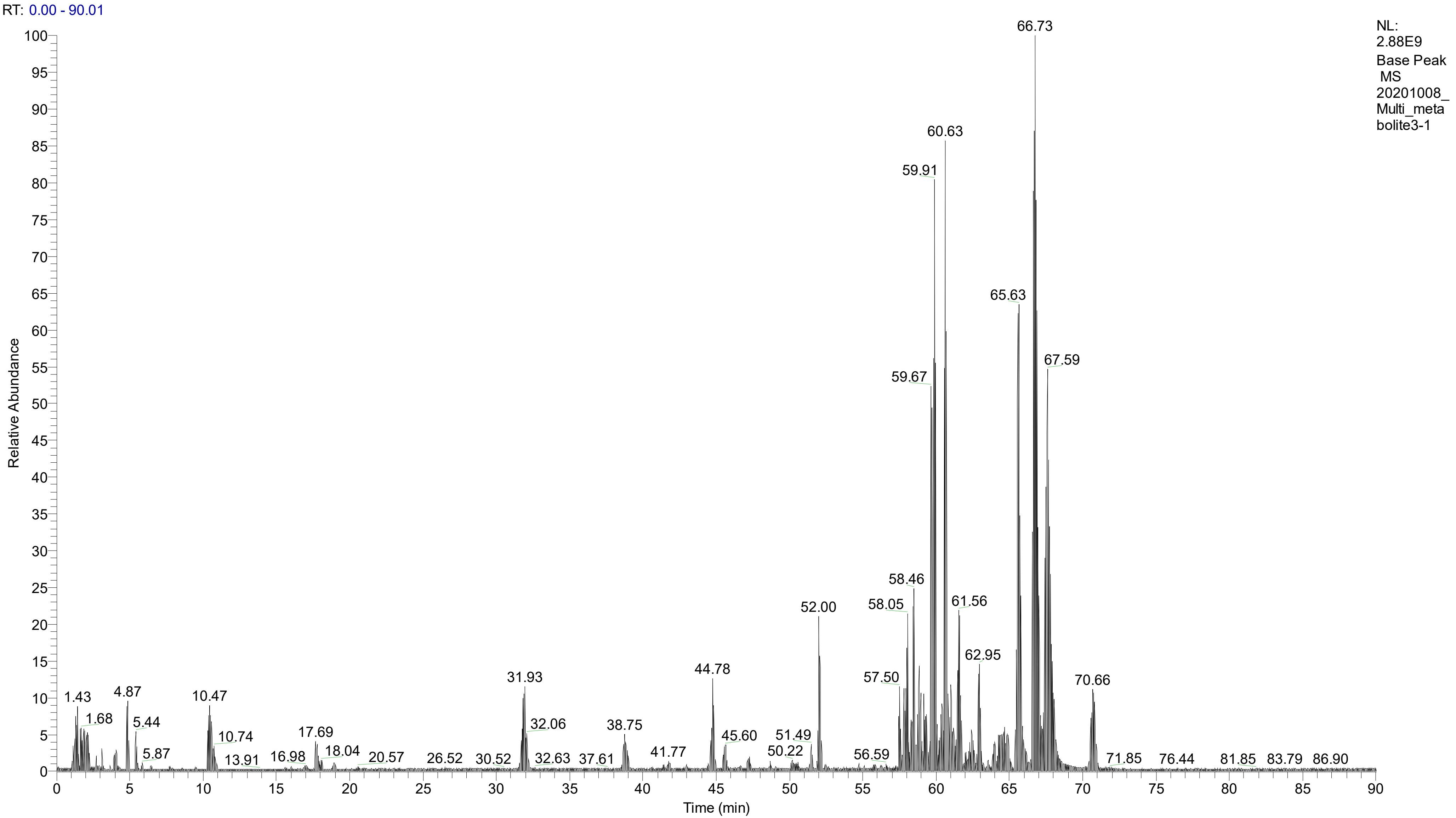
RT: 0.00 - 90.01
NL:
2.88E9
Base Peak
MS
20201008_
Multi_meta
bolite3-1
Relative Abundance
Time (min)
66.73
60.63
59.91
65.63
67.59
59.67
58.46
58.05
61.56
52.00
62.95
57.50
44.78
31.93
70.66
1.43
4.87
10.47
1.68
5.44
10.74
17.69
32.06
38.75
45.60
51.49
5.87
13.91
16.98
18.04
20.57
26.52
30.52
32.63
37.61
41.77
50.22
56.59
71.85
76.44
81.85
83.79
86.90

(C)MTBE-2

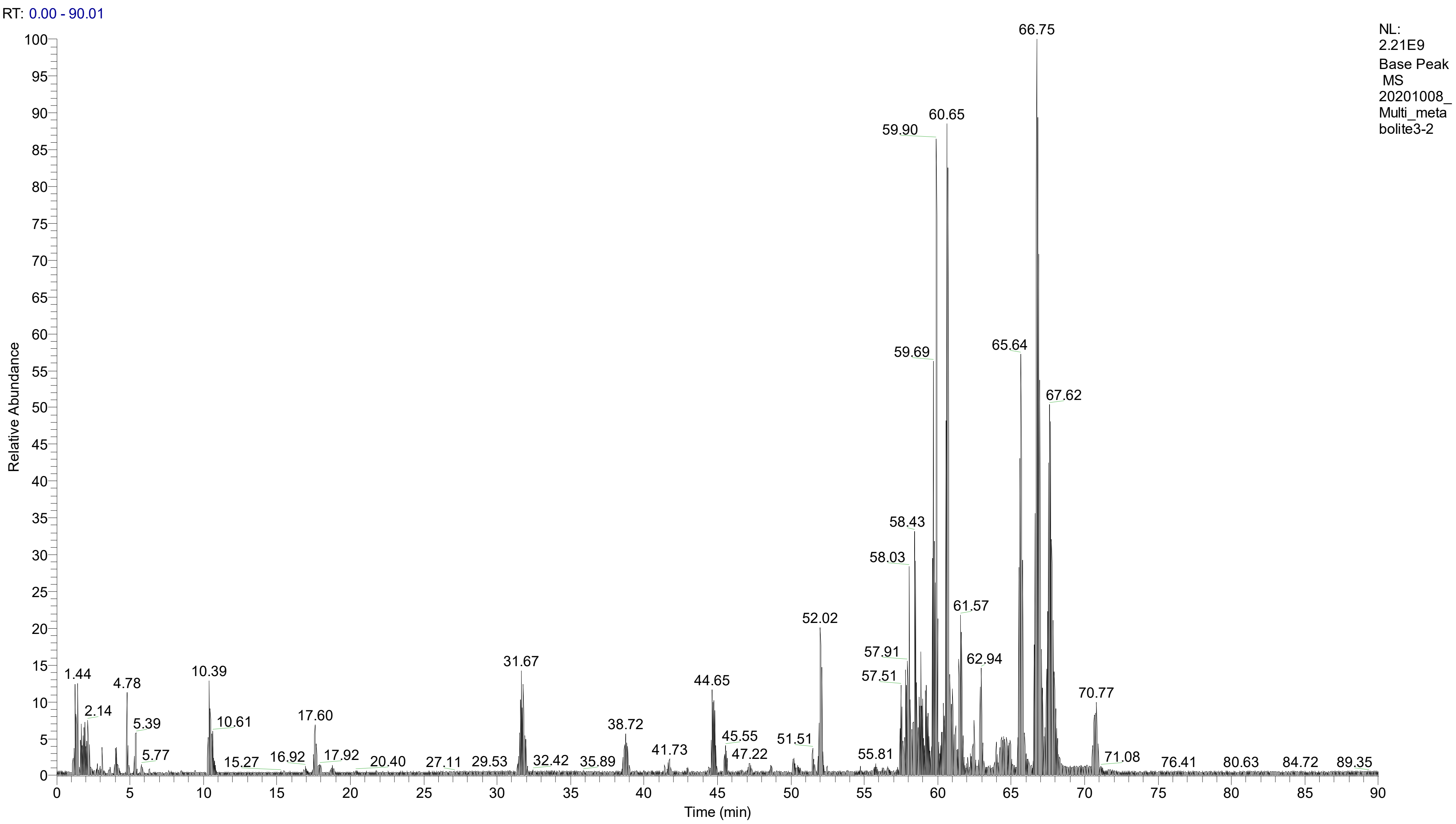

RT: 0.00 - 90.01
NL:
2.21E9
Base Peak
MS
20201008_
Multi_meta
bolite3-2
Relative Abundance
Time (min)
66.75
60.65
59.90
59.69
65.64
67.62
58.43
58.03
61.57
52.02
57.91
62.94
57.51
31.67
1.44
4.78
10.39
44.65
70.77
2.14
5.39
10.61
17.60
38.72
45.55
51.51
5.77
15.27
16.92
17.92
20.40
27.11
29.53
32.42
35.89
41.73
47.22
55.81
71.08
76.41
80.63
84.72
89.35

(C)MTBE-3

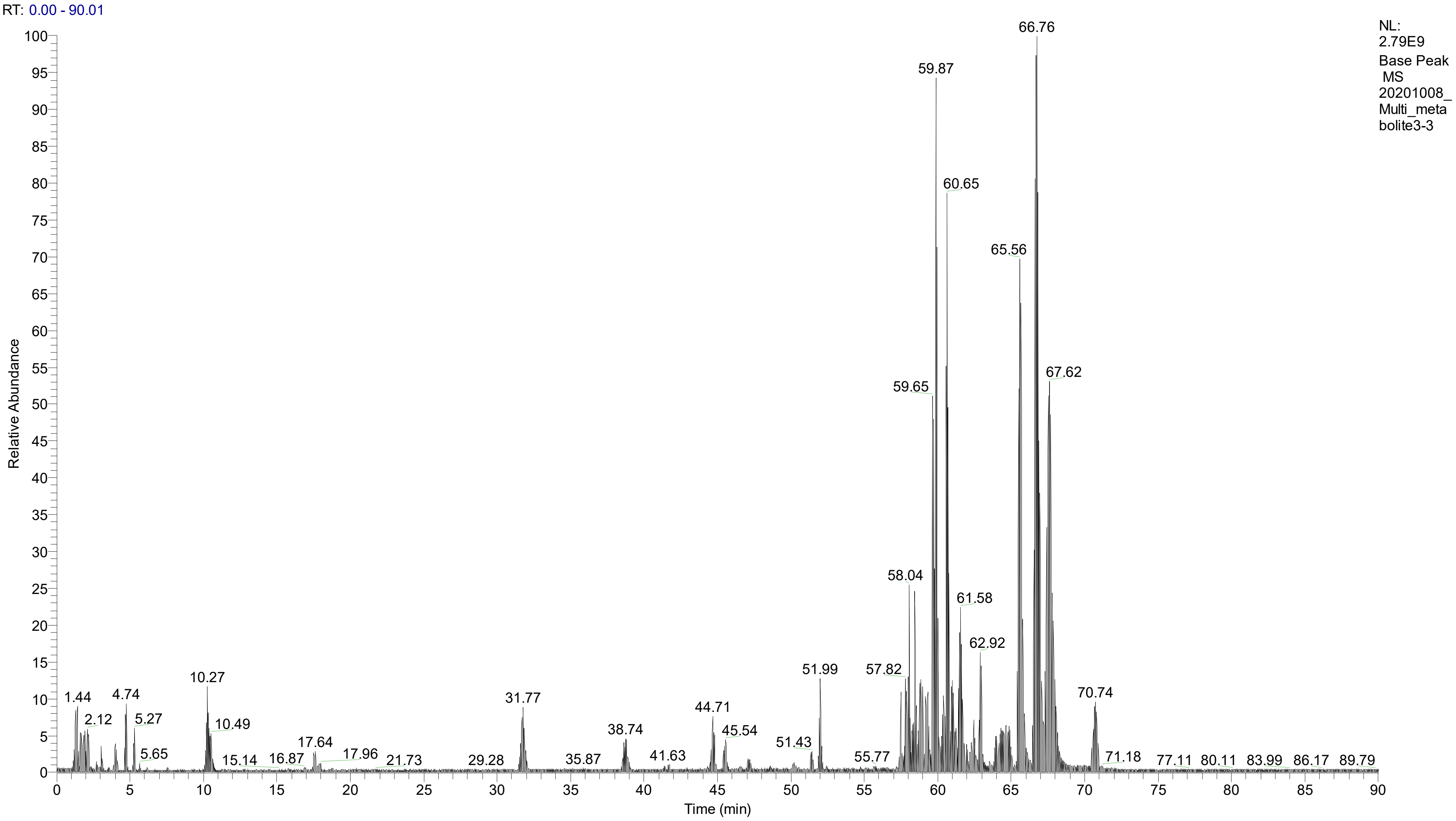

Figure S4 Chromatogram of metabolomics data of each method.

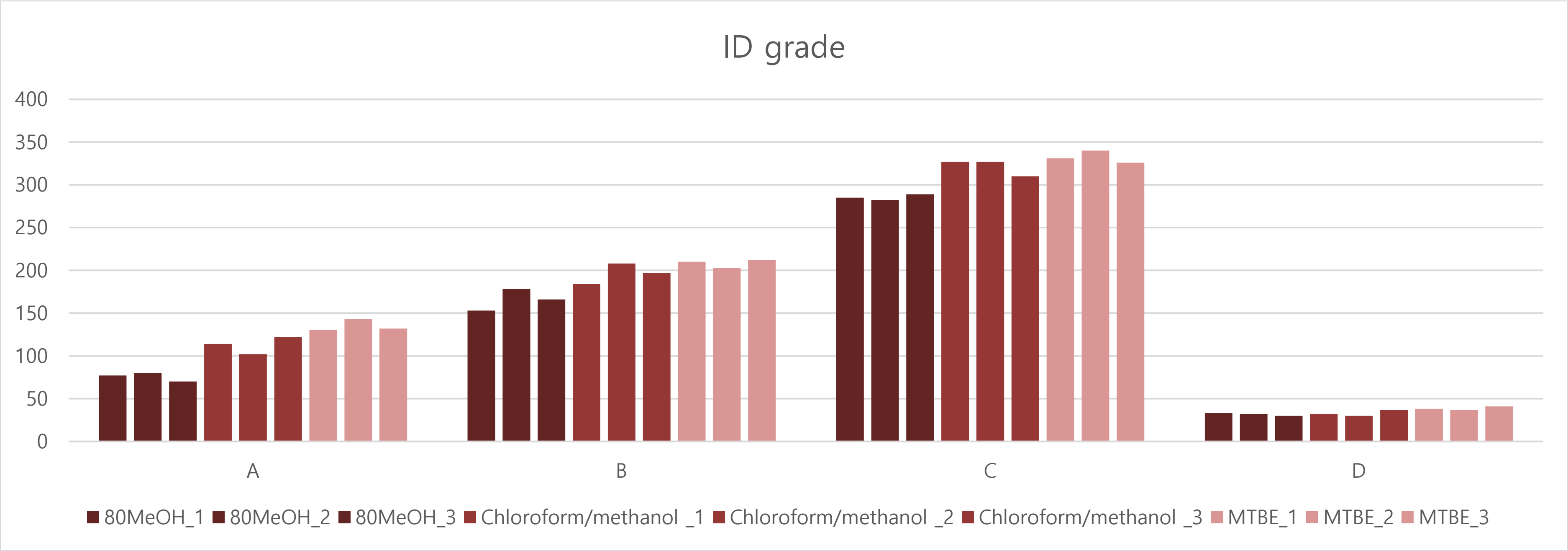

Figure S5. ID grade graph for each extraction method of lipids identified by lipid search. (ID Quality filter- Grade A : Lipid of which fatty acid chains and class were identified completely./ Grade B : Lipid of which class and some fatty acid chains were identified./Grade C : Lipid of which class or fatty acid was identified./Grade D : other)

(A)80%methanol-1

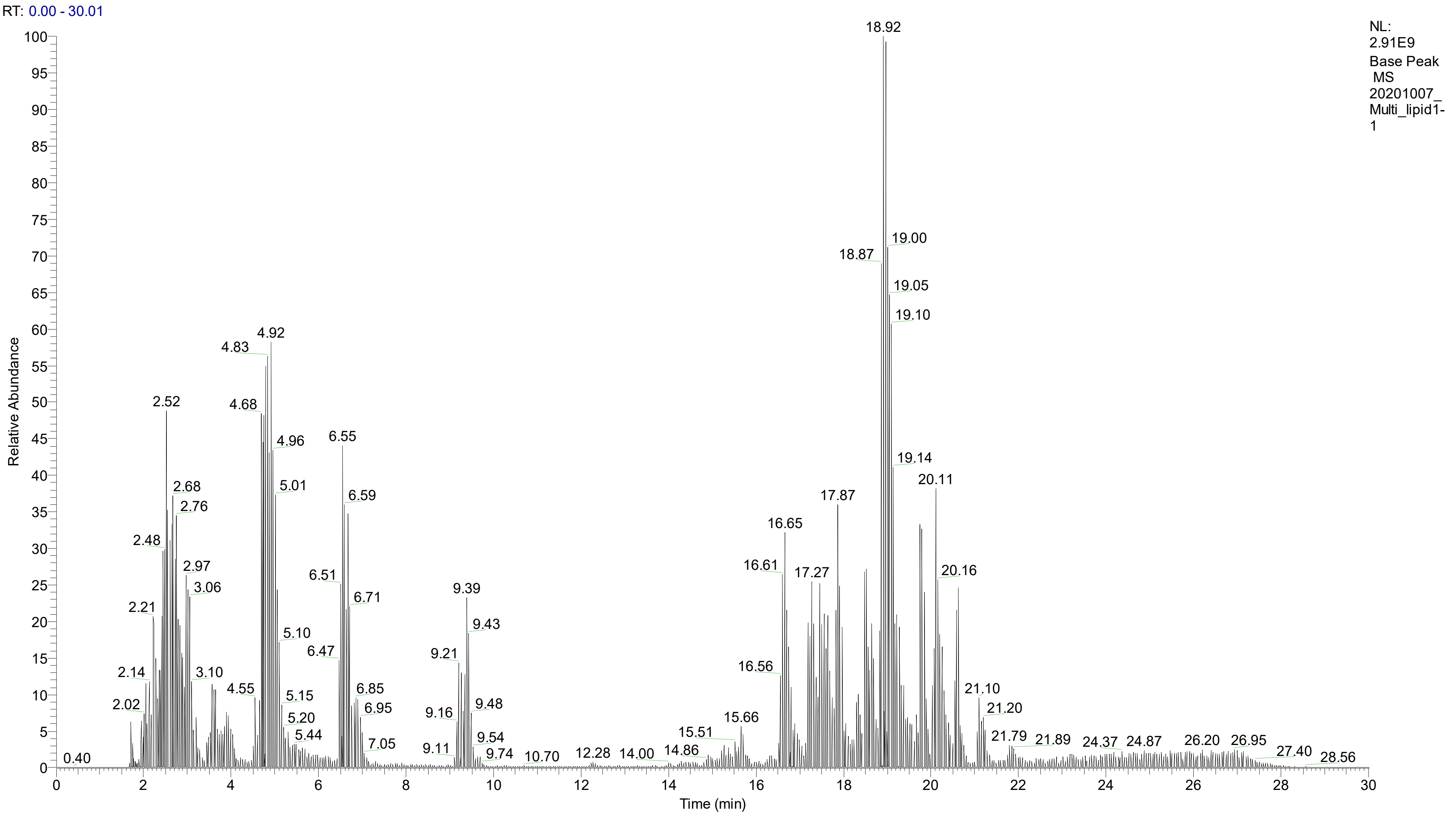

(A)80%methanol-2

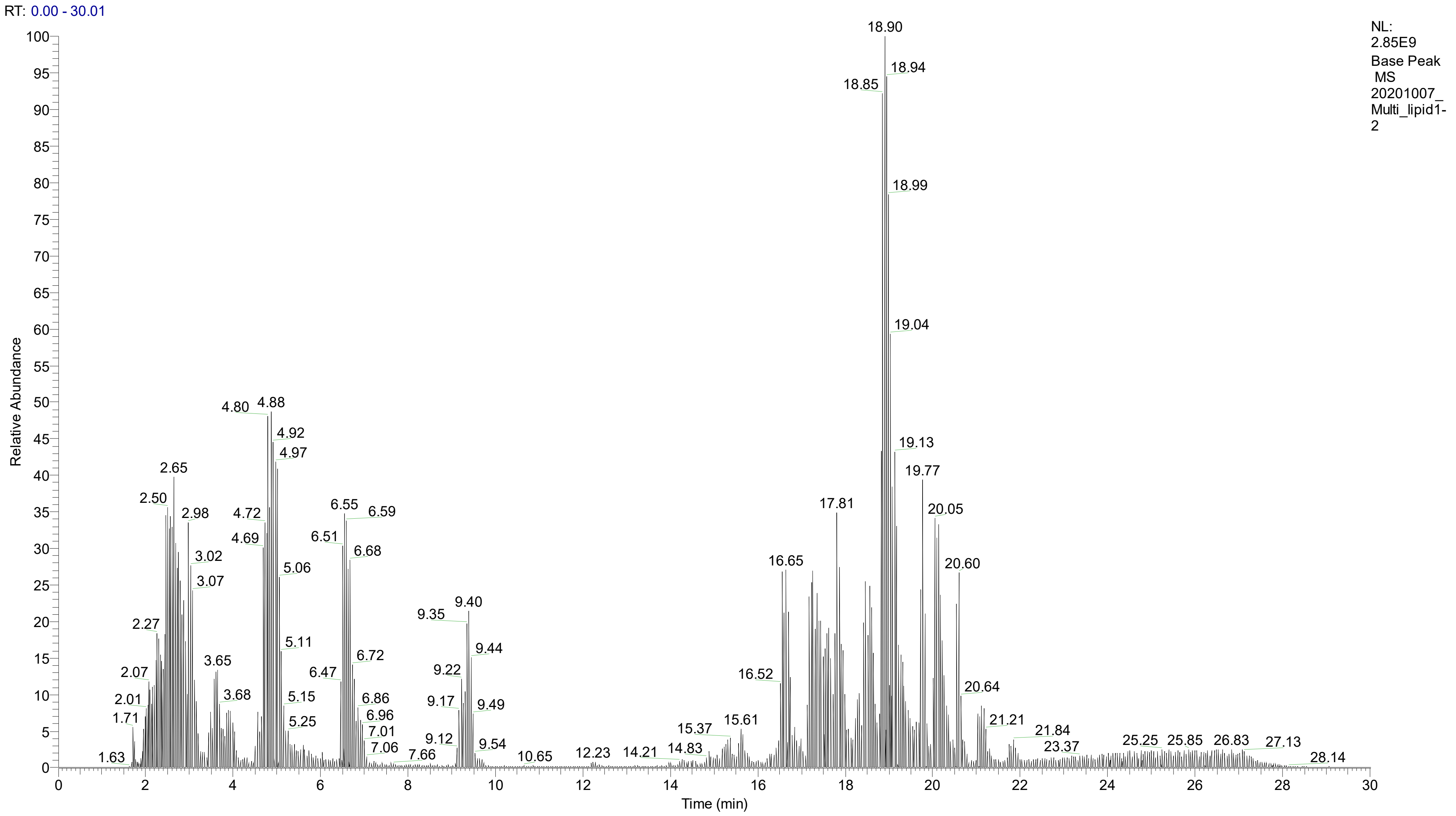

(A)80%methanol-3

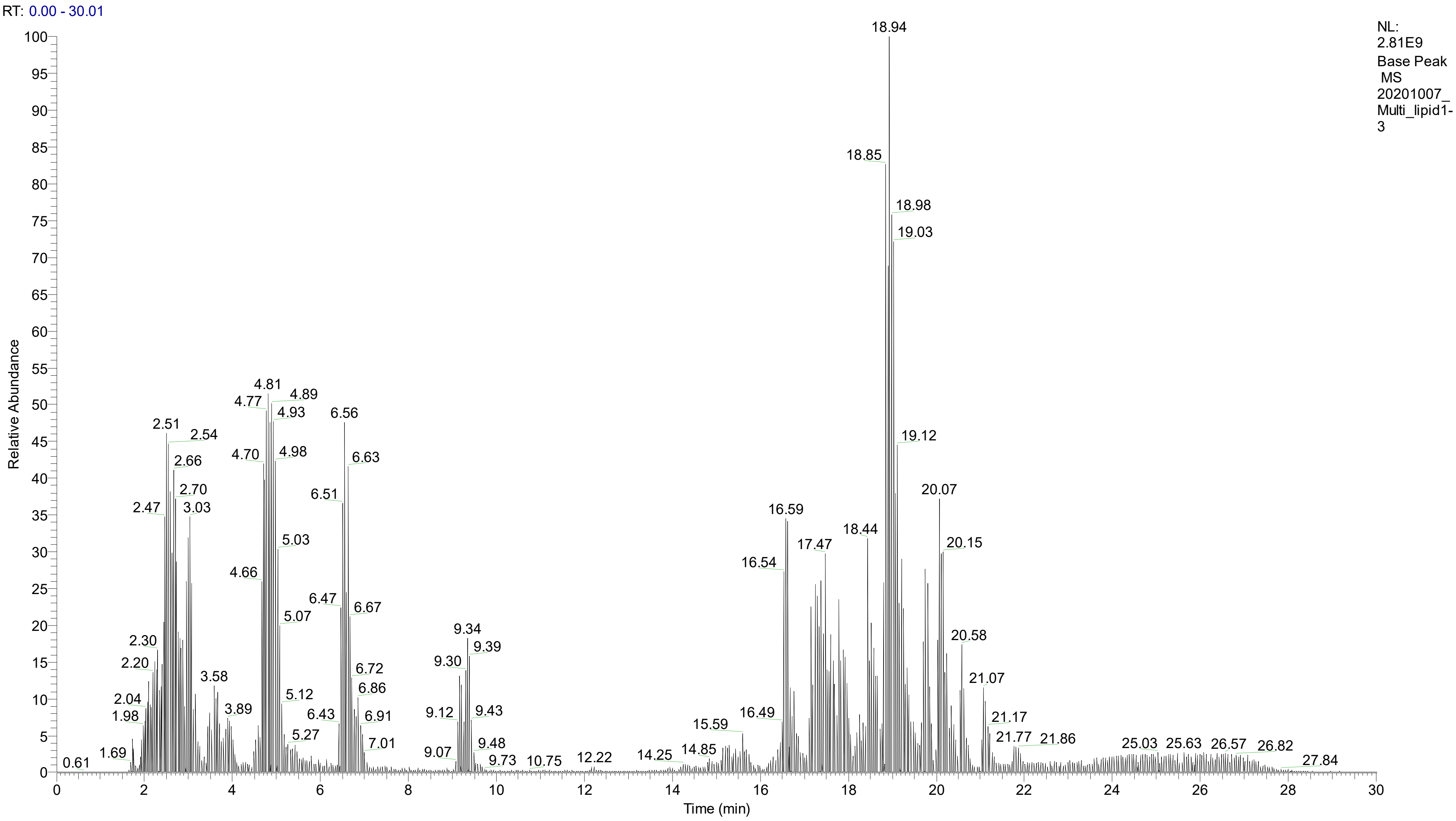

(B)Chloroform/methanol-1

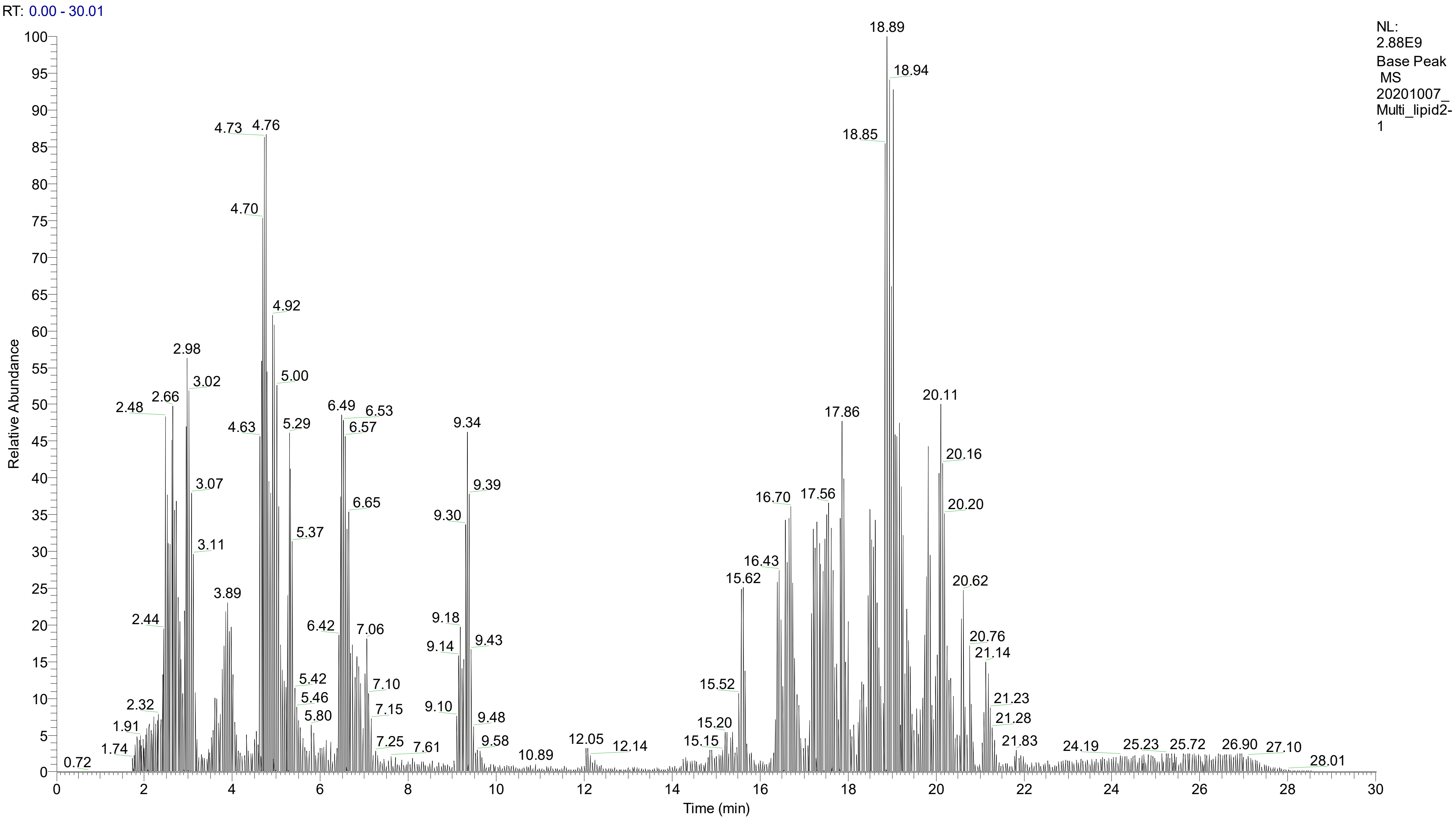

(B)Chloroform/methanol-2

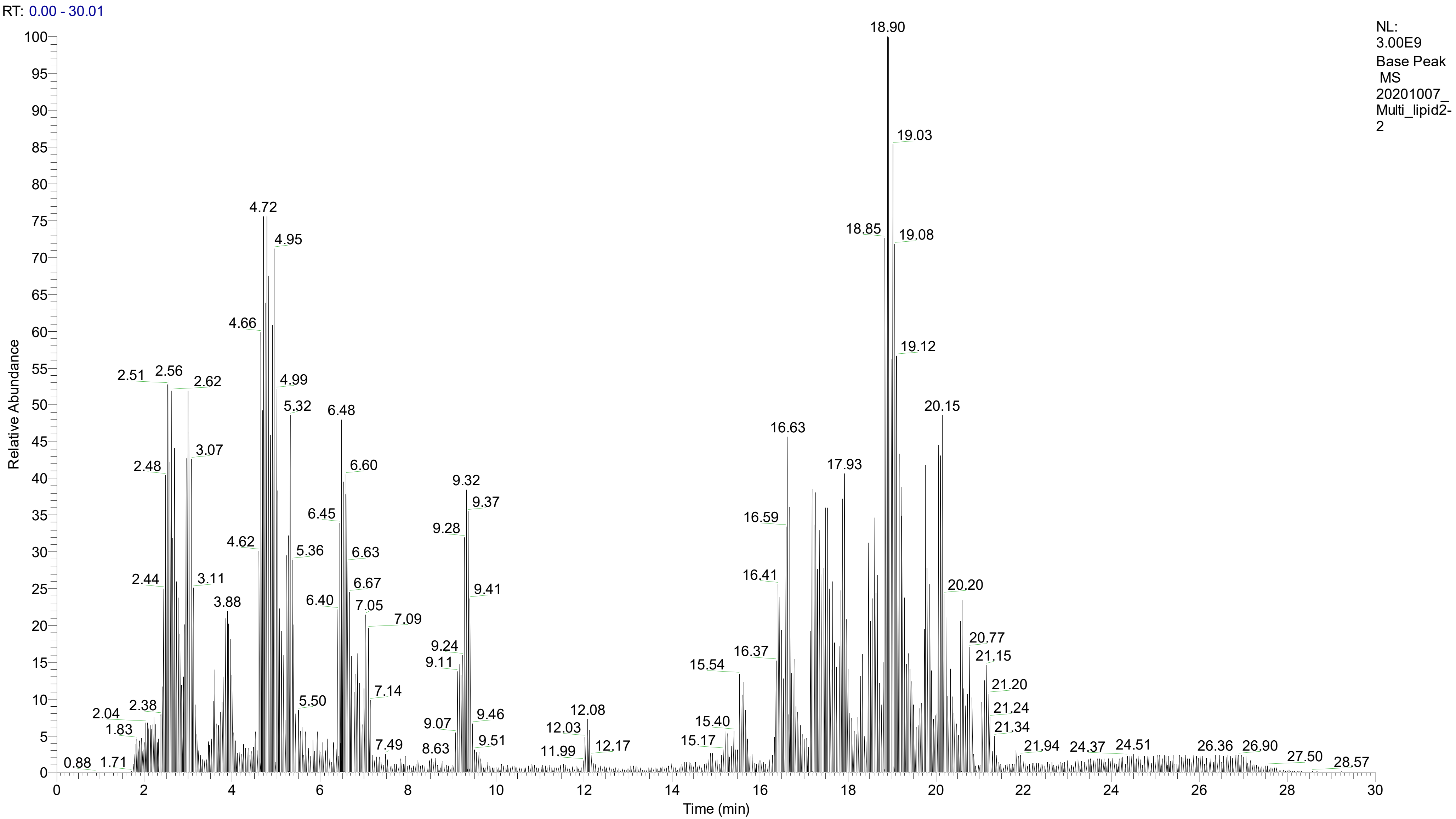

(B)Chloroform/methanol-3

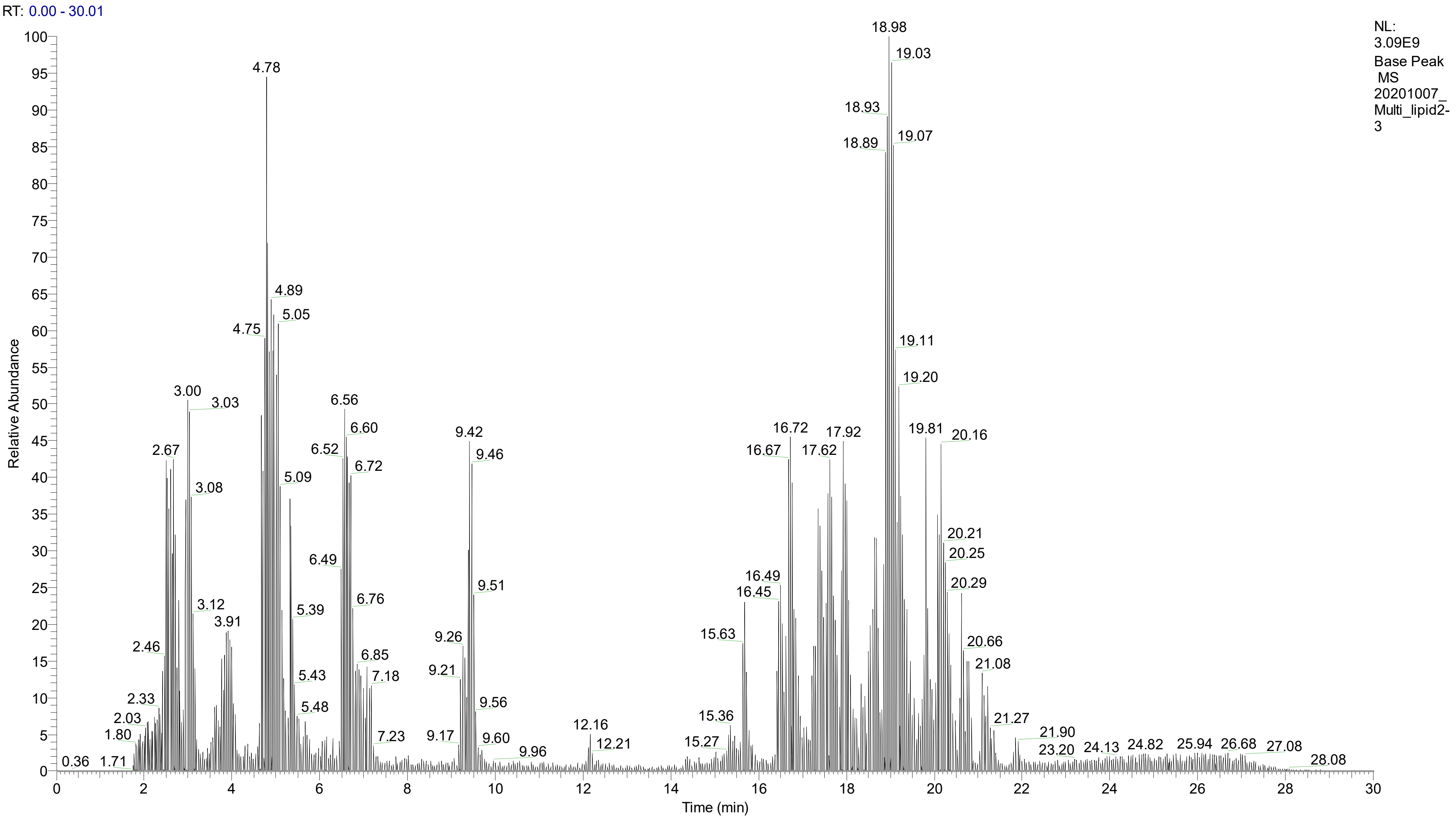

(C)MTBE-1

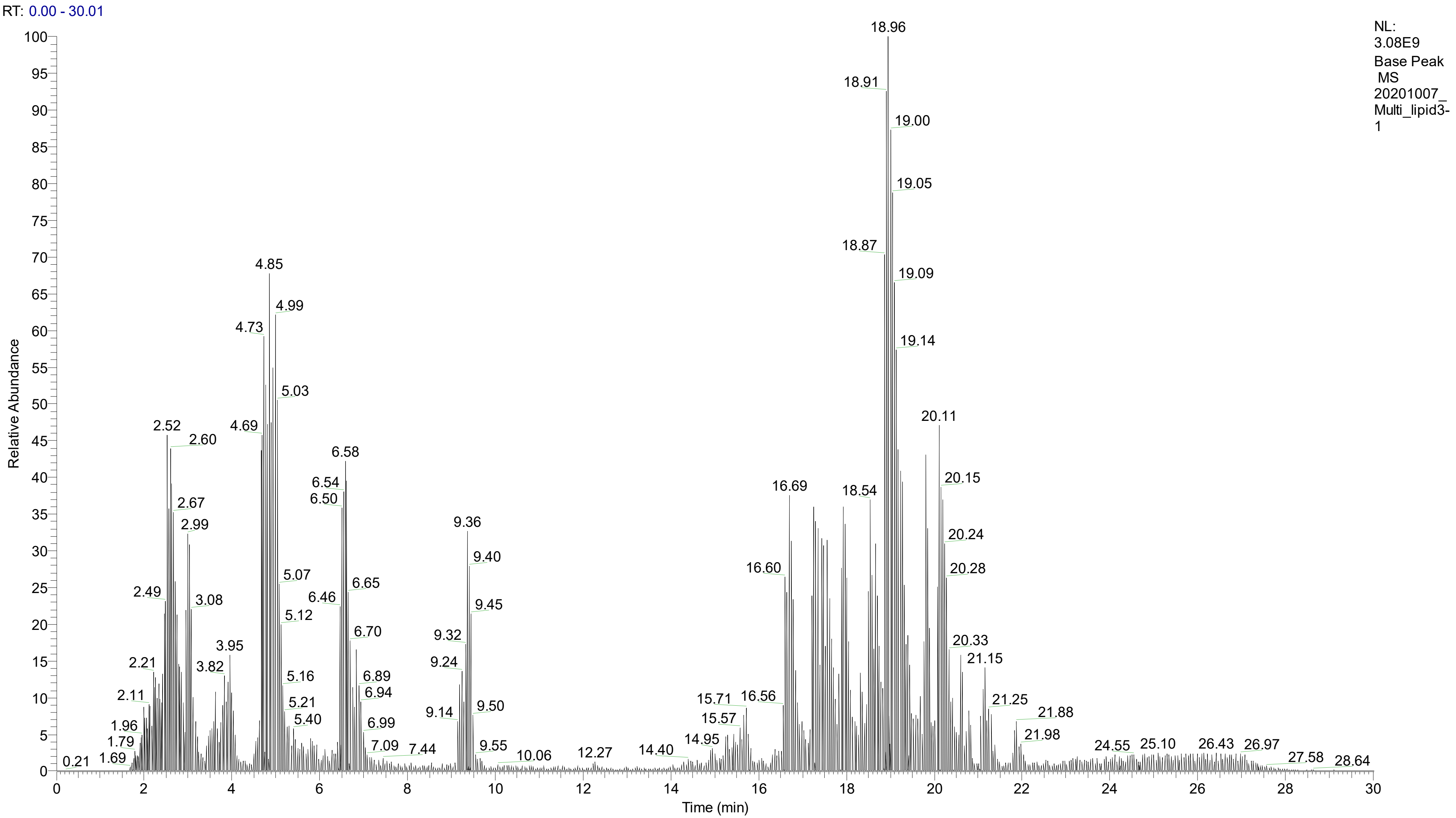

RT: 0.00 - 30.01
NL: 3.08E9 Base Peak MS 20201007_Multi_lipid3-1
Relative Abundance
Time (min)
18.96
18.91
19.00
19.05
18.87
19.09
19.14
20.11
20.15
20.24
20.28
20.33
21.15
21.25
21.88
21.98
16.69
16.60
16.56
18.54
15.71
15.57
14.95
14.40
12.27
10.06
9.36
9.40
9.45
9.32
9.24
9.14
9.50
9.55
4.85
4.99
4.73
5.03
4.69
5.07
5.12
5.16
5.21
5.40
6.58
6.54
6.50
6.46
6.65
6.70
6.89
6.94
6.99
7.09
7.44
2.52
2.60
2.67
2.99
2.49
3.08
3.95
3.82
2.21
2.11
1.96
1.79
1.69
0.21
24.55
25.10
26.43
26.97
27.58
28.64

(C)MTBE-2

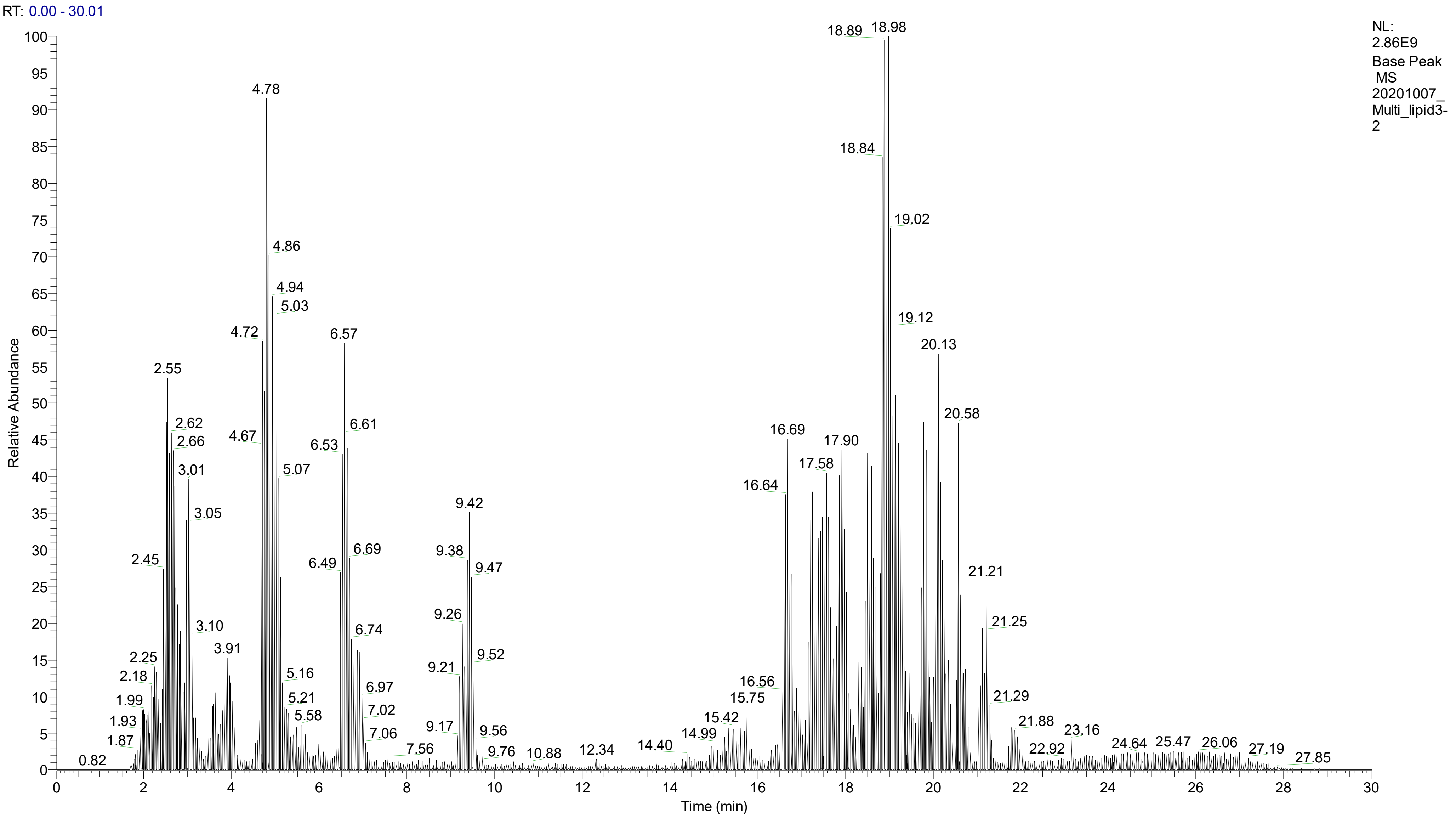
RT: 0.00 - 30.01
NL: 2.86E9 Base Peak MS 20201007_Multi_lipid3-2
Relative Abundance
Time (min)
18.89
18.98
4.78
18.84
19.02
4.86
4.94
5.03
4.72
6.57
19.12
20.13
2.55
20.58
2.62
6.61
4.67
2.66
6.53
16.69
17.90
5.07
3.01
17.58
16.64
9.42
3.05
2.45
9.38
6.69
6.49
9.47
21.21
9.26
21.25
3.10
6.74
3.91
2.25
9.52
2.18
9.21
5.16
16.56
6.97
15.75
1.99
5.21
21.29
15.42
7.02
5.58
1.93
21.88
9.17
23.16
9.56
14.99
1.87
7.06
14.40
24.64
25.47
26.06
12.34
7.56
22.92
9.76
10.88
27.19
0.82
27.85

(C)MTBE-3

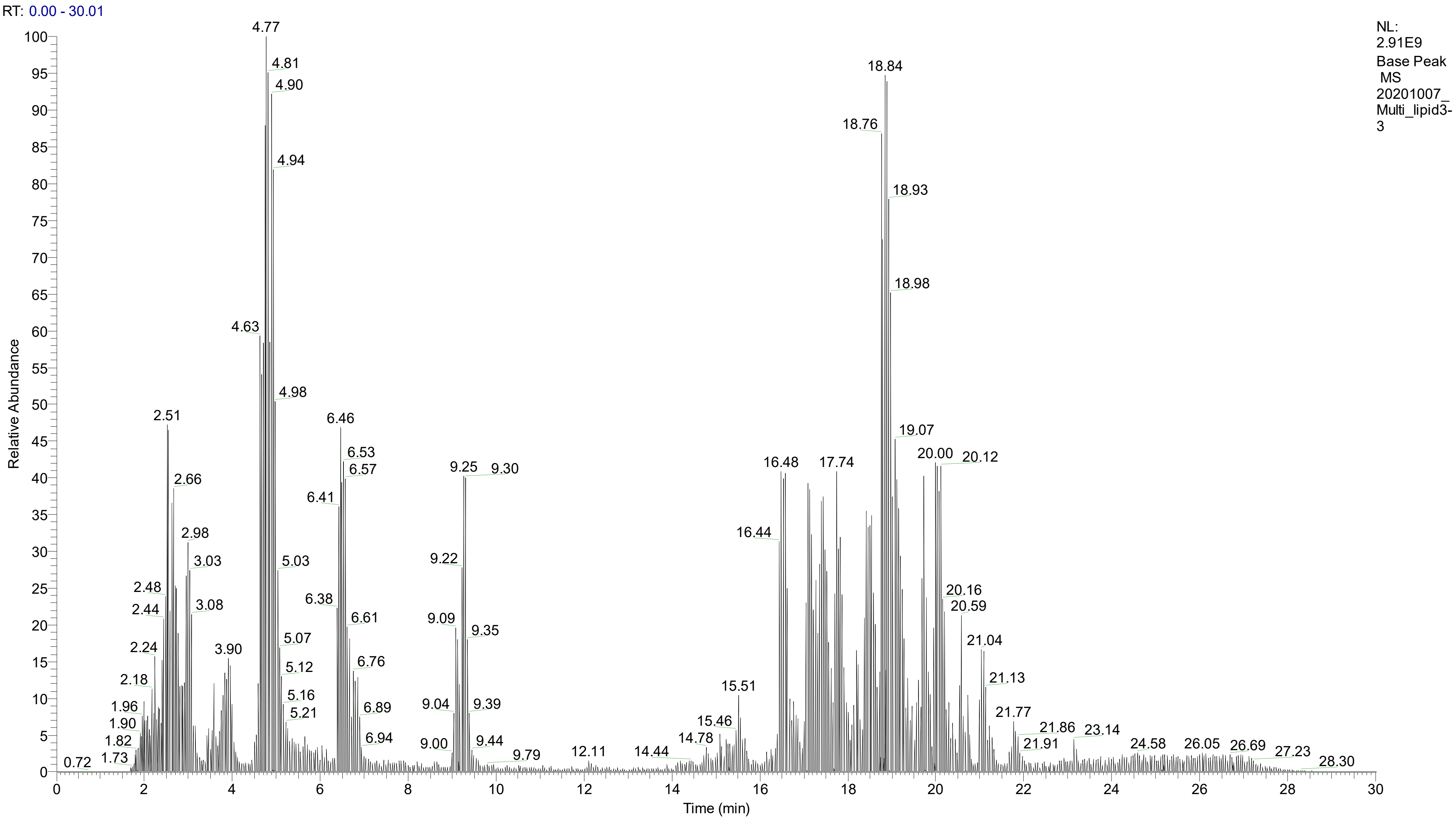

Figure S6 Chromatogram of lipidomics data of each method.

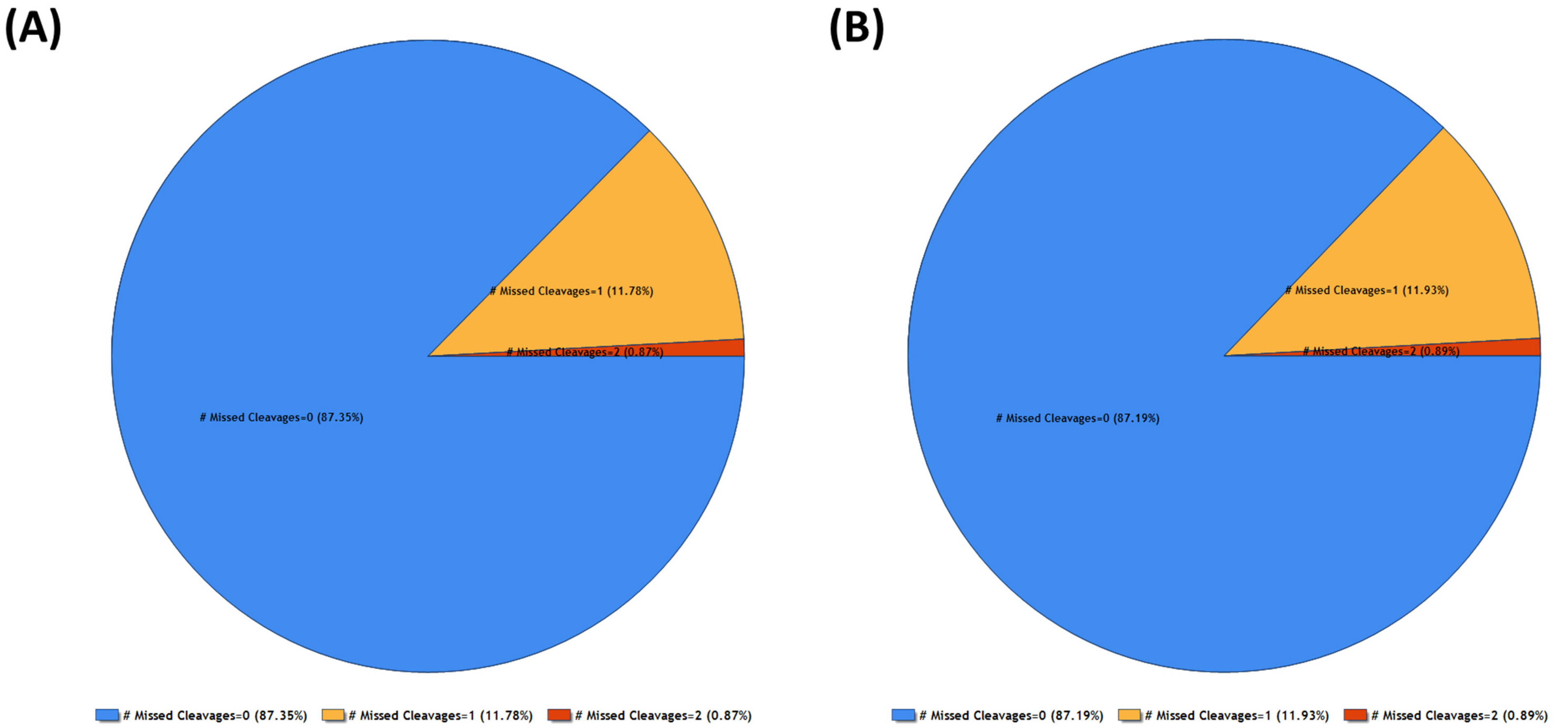
(A)
Missed Cleavages=1 (11.78%)
Missed Cleavages=2 (0.87%)
Missed Cleavages=0 (87.35%)
Missed Cleavages=0 (87.35%) # Missed Cleavages=1 (11.78%) # Missed Cleavages=2 (0.87%)
(B)
Missed Cleavages=1 (11.93%)
Missed Cleavages=2 (0.89%)
Missed Cleavages=0 (87.19%)
Missed Cleavages=0 (87.19%) # Missed Cleavages=1 (11.93%) # Missed Cleavages=2 (0.89%)

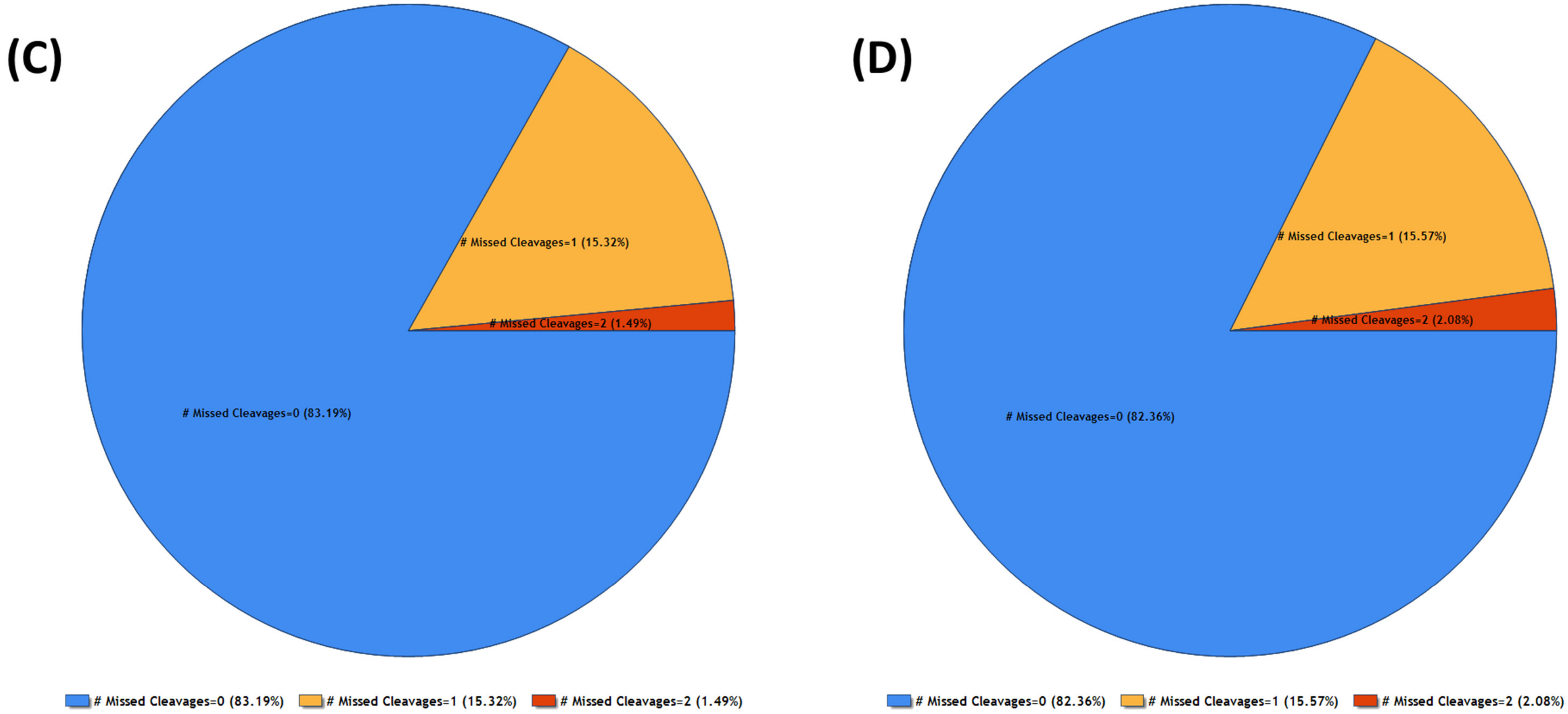
(C)
Missed Cleavages=1 (15.32%)
Missed Cleavages=2 (1.49%)
Missed Cleavages=0 (83.19%)
Missed Cleavages=0 (83.19%) # Missed Cleavages=1 (15.32%) # Missed Cleavages=2 (1.49%)
(D)
Missed Cleavages=1 (15.57%)
Missed Cleavages=2 (2.08%)
Missed Cleavages=0 (82.36%)
Missed Cleavages=0 (82.36%) # Missed Cleavages=1 (15.57%) # Missed Cleavages=2 (2.08%)

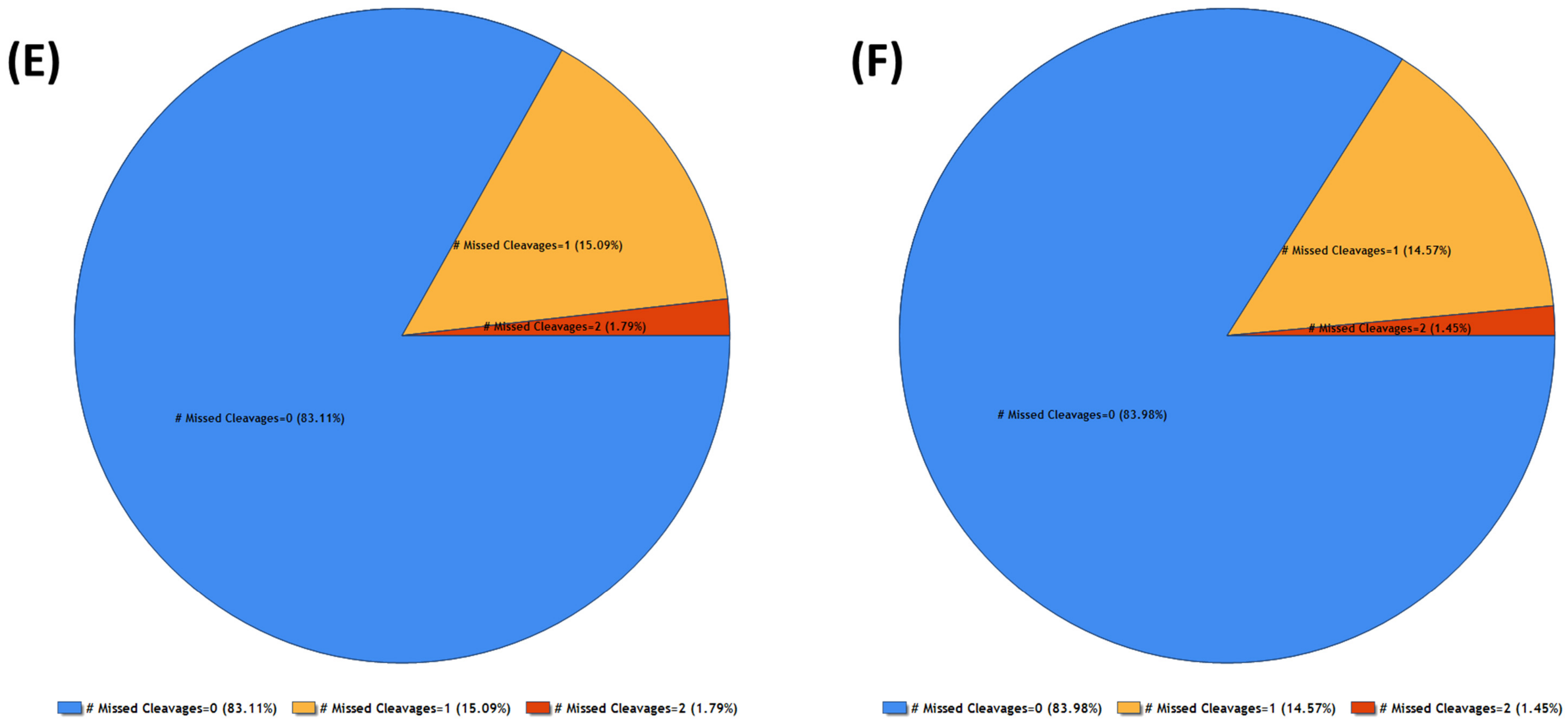

Figure S7 Number of missed cleavages in identified peptides. (A) FASP_80methanol, (B) FASP_Chloroform/methanol, (C) FASP_MTBE, (D) S-trap_80methanol, (E) S-trap_Chloroform/methanol , (F) S-trap_MTBE

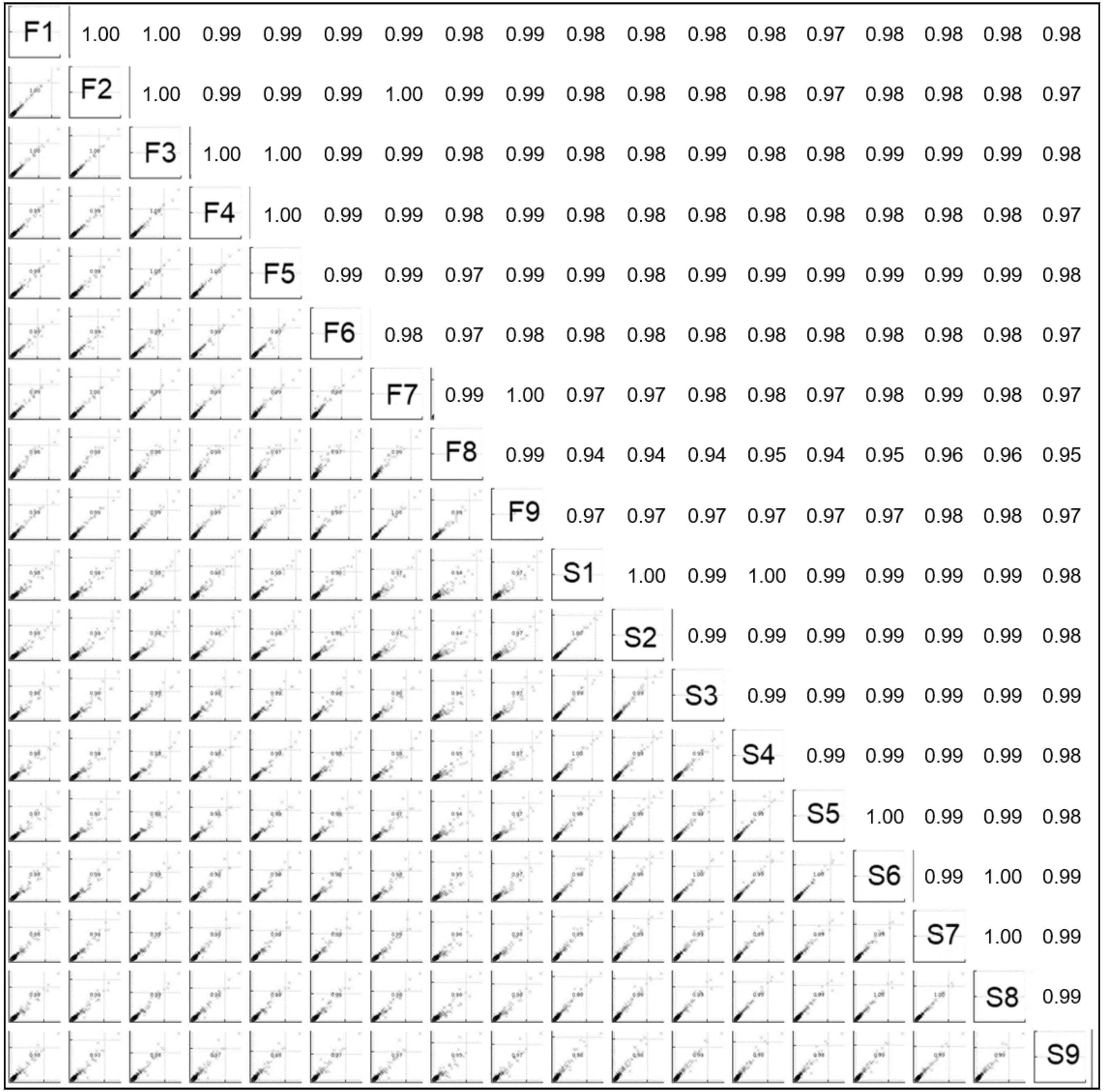

Figure S8 Correlation scatter plot in proteomics data

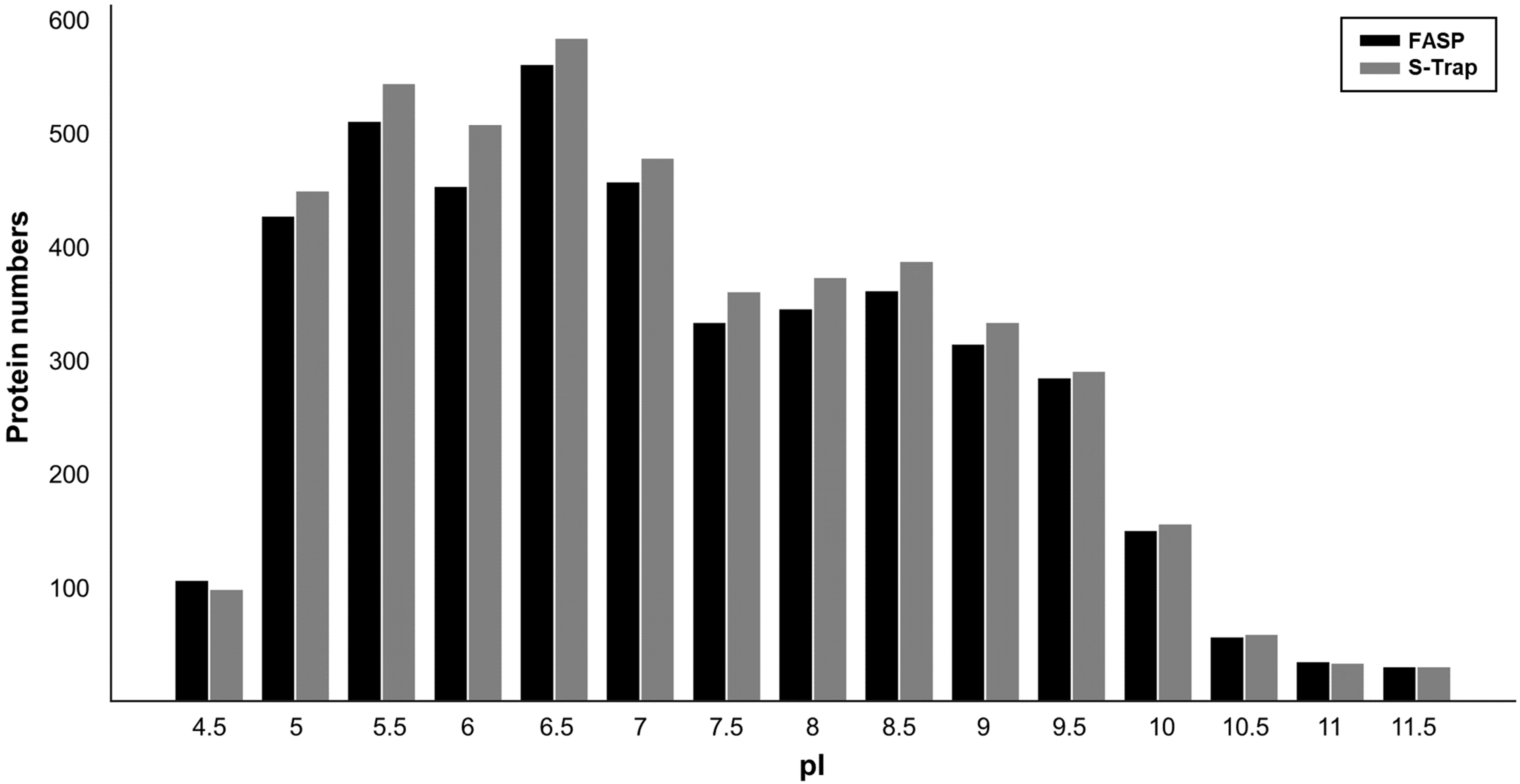

Figure S9. Histogram of pI(isoelectric points)

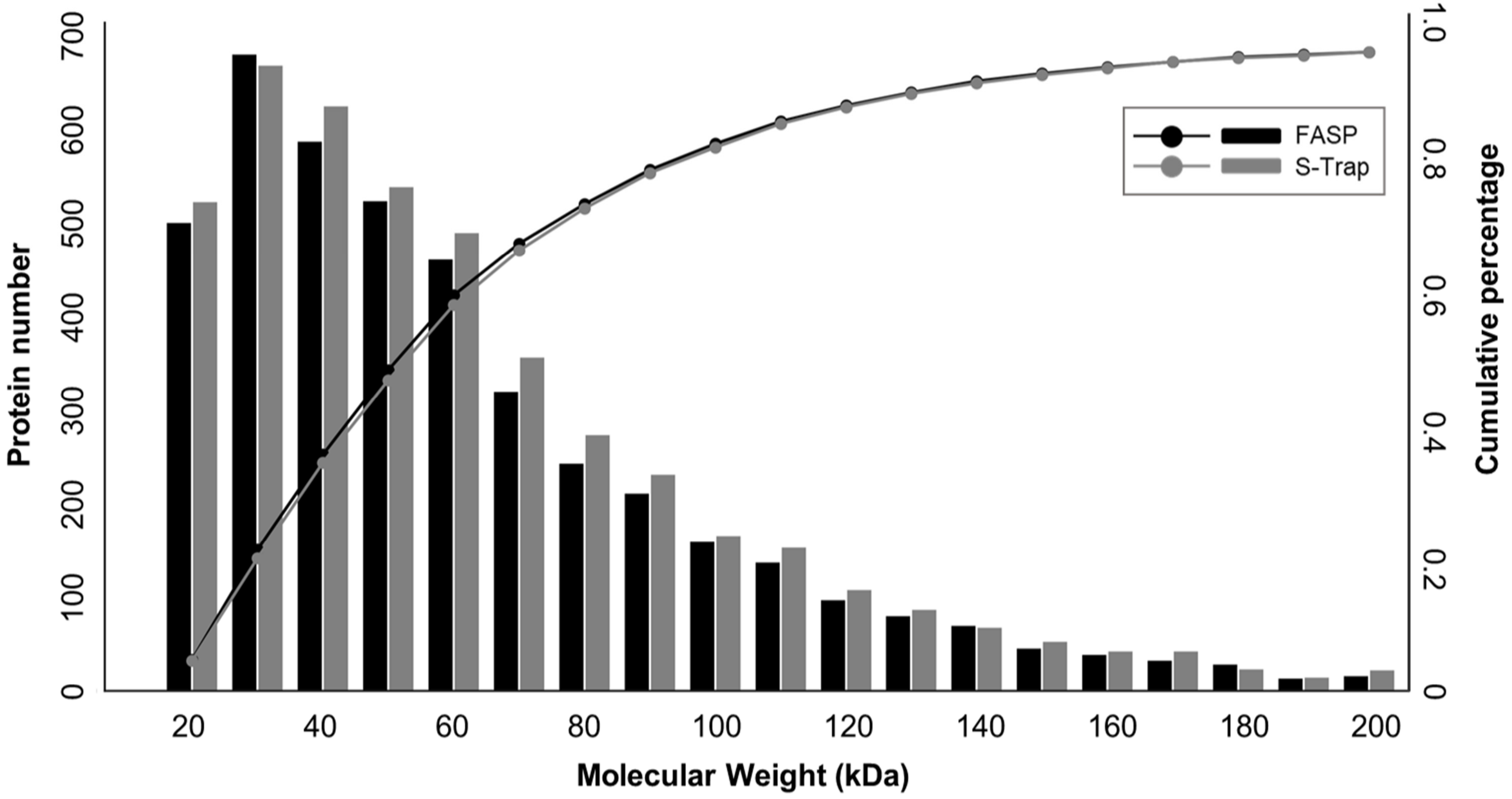

Figure S10. Histograms and cumulative distributions of molecular weight(MW)

FASP/S-Trap	log2(FC)	raw.pval	Gene name	Discription
Q71DI3	2.079	1.58E-07	H3C15	Histone H3.2
Q9BW91	-1.0016	0.00020524	NUDT9	ADP-ribose pyrophosphatase mitochondrial
Q7Z417	-1.075	0.0067655	NUDT9	FMR1-interacting protein NUFIP2

Table S2. Fold change and p-value of Q71DI3, Q9BW91, Q7Z417
